# Phytochemical, Nutritional and Physicochemical Characteristics of Fruits and Leaves from the *Myrica* L. Genus: A Systematic Review

**DOI:** 10.3390/ijms27188237

**Published:** 2026-09-16

**Authors:** Sofia G. Florença, Maria João Barroca, Maria João Lima, Cláudia Nunes

**Affiliations:** 1 CERNAS-IPV Research Centre, Polytechnic University of Viseu, 3504-510 Viseu, Portugal; sofiaflorenca@outlook.com (S.G.F.); mjoaolima@esav.ipv.pt (M.J.L.); 2 Department of Chemistry, University of Aveiro, 3810-193 Aveiro, Portugal; 3 CICECO—Aveiro Institute of Materials, Department of Materials and Ceramic Engineering, University of Aveiro, 3810-193 Aveiro, Portugal; 4 Unidade de I&D Química-Física Molecular, Department of Chemistry, University of Coimbra, 3004-535 Coimbra, Portugal; mjbarroca@esac.pt; 5 Polytechnic Institute of Coimbra, Coimbra Agriculture School, Bencata, 3045-601 Coimbra, Portugal

**Keywords:** *Myrica*, fruits, leaves, phytochemical composition, nutritional value

## Abstract

The *Myrica* L. genus represents a group of plants commonly known as actinorhizal plants, which have been used as food, medicine, and natural dyes. This article aims to systematically review the available recent scientific evidence identified through a defined search strategy on the phytochemical, physical, and nutritional characteristics of the fruits and leaves of the species from the *Myrica* L. genus. The search was conducted across three databases, following the Preferred Reporting of Items for Systematic Reviews and Meta-analysis statement guidelines. Studies published in English, as open-access articles, between 2014 and 2024, and addressing the aim of the review were included, resulting in 16 eligible articles. Concerning the physicochemical characteristics of the fruits, the research indicates variability among species and within the same species. The nutritional results are limited, with one study reporting carbohydrates and proteins as the main macronutrient components–79.84% and 25.02% respectively–in *Myrica esculenta* fruits, although with limited interpretation. Furthermore, numerous phytochemicals have been identified and quantified, some with reported biological activities. For example, myricetin concentrations in *Myrica rubra* fruits ranged from 0.002 to 1.27 mg/g. In conclusion, this review highlights the nutritional and phytochemical potential of *Myrica* L. species and the wide range of chemical compounds reported, while acknowledging the limited number of studies and the methodological heterogeneity, among other limitations.

## 1. Introduction

Plants, belonging to one of the biggest kingdoms of life, stand at the base of all ecosystems, supporting a pivotal role in all forms of life on Earth, including humans. Since the beginning of human history, plants have had a crucial part in humankind’s survival and thriving [1,2,3,4,5,6,7].

The *Myricaceae* family is represented by aromatic and resinous shrubs or small trees with nitrogen-fixing roots, discreet, unisexual, dicotyledonous flowers, and single-seeded fruits frequently covered with waxy granules. It is a group of plants that thrives in temperate to subtropical regions and encompasses three genera—*Myrica*, *Canacomyrica* and *Comptonia* [8,9,10].

The *Myrica* L. genus is the largest and most diverse genus in the *Myricaceae* family, comprising 50 species, with a wide geographical distribution in Africa, Europe, Asia, and North and South America, occurring in temperate, subtropical and tropical regions [8,10,11]. This genus is taxonomically defined as belonging to the Plantae kingdom, Streptophyta phylum, Equisetopsida class, Magnoliidae subclass, Fagales order, and *Myricaceae* family [12,13]. Plants from this genus are generally evergreen or deciduous shrubs or small trees, depending on the species and climate. By fixing substantial amounts of nitrogen in the soil, many species within the *Myrica* L. genus facilitate the development of other plant communities [14].

All parts of *Myrica* L. plants have potential economic value, being used for paper, rope, biomass production, wax, food and medicinal purposes. The fruits can be consumed fresh or processed into juices, wines and jams. Additionally, these plants produce a wide range of bioactive compounds with beneficial health properties, making them valuable in traditional and modern medicine. *Myrica* L. species have been used to treat various ailments, including diarrhea, skin disorders, gastroenteritis, headaches, burns, asthma, bronchitis, fever, respiratory infections, jaundice, sore throat, ulcers and abdominal cramps, due to their antioxidant, antimicrobial, anti-tumor, anti-diabetic, anti-obesity, and anti-inflammatory properties [15,16,17].

Plants synthesize a diverse range of chemical compounds, known as secondary metabolites, which do not directly contribute to the growth or development processes. Their production often increases in response to internal or external stressors or to attract pollinators [7,18,19,20].

Polyphenols, also known as phenolic compounds, are secondary metabolites naturally occurring in vegetables, fruits, and grains. These compounds are widely used in the pharmaceutical and food industries due to their benefits to human health, thanks to their antioxidant, antifungal, and antimicrobial properties, as well as their influence on the taste and texture of food items [18,20,21,22]. The primary categories of polyphenols include phenolic acids, lignans, stilbenes, and flavonoids [19,20].

Phenolic acids are involved in the plant’s development, reproduction, and defense against external factors. These compounds have health benefits against hypertension, depression, inflammation, cancer, hyperglycemia, type 2 diabetes, diarrhea and neurologic diseases [18,19,20].

Additionally, lignans underscore the diverse functional roles of phenolic compounds in both plant physiology and potential therapeutic applications [20,23].

Stilbenes, namely, resveratrol, possess a potential therapeutic effect in preventing cardiovascular disease, diabetes, cancer, neurological disorders, inflammation, and obesity [20,24].

Similarly, flavonoids, like stilbenes, represent a diverse group of secondary metabolites with various studies showing an important antioxidant capacity and ability to alleviate inflammation, improve Alzheimer’s symptoms, work as an anti-carcinogenic agent, and protect against cardiovascular diseases, diabetes, and bacterial, parasitical, and viral infections [18,20,25,26].

A nutritious and well-balanced diet is essential for health. Numerous food products that constitute a healthy diet, such as vegetables, fruits, herbs, spices, and grains are notably rich in polyphenols. Polyphenols are bioactive compounds that play vital roles in the biological functions of plants and possess the potential to mitigate and prevent chronic diseases, such as diabetes, cancer, dyslipidemia, cardiovascular disease, age-related disorders, and neurological conditions, and act as an exogenous antioxidant [18,19,20,22,25,27].

Existing scientific reviews regarding the *Myrica* L. genus assemble information on the secondary metabolites and their biological properties relevant to human health of the *Morella* and *Myrica* L. species [28], provide an overview of recent findings on the antioxidant and anti-inflammatory effects of the *Morella* and *Myrica* L. species [17], and address the phytochemical, pharmaceutical, nutritional, and health aspects of specific species from the *Myrica* L. genus [16,29,30,31,32,33,34,35,36,37,38].

To date, the available literature appears not to have provided a synthesis of the existing evidence on the phytochemical, physical and nutritional characteristics of species within the *Myrica* L. genus, with a specific focus on fruits and leaves. The scientific contribution of this study resides in a systematic comparison of the accessible reported data concerning fruits and leaves across different *Myrica* L. species, taking dispersed information and creating a unified perspective. Although previous studies have addressed secondary metabolites, biological activities and antioxidant properties in articles compiling different *Myrica* L. species, reviews focusing on phytochemical composition have been limited to individual species. Thus, the available literature appears to contain limited information that systematically compiles and compares the accessible data on chemical composition across multiple *Myrica* L. species, integrating it all into one article.

Furthermore, this study adopts a systematic review approach, ensuring methodological rigor through adherence to guidelines and the inclusion of an author-developed exploratory study-quality assessment, providing an analysis of the published scientific evidence identified through a defined search strategy on the phytochemical, physical, and nutritional characteristics of different *Myrica* L. species.

Therefore, this study seeks to elucidate the value of the *Myrica* L. genus as a source of nutrients and potential new chemical compounds, aiming to: (1) analyze the chemical composition and phytochemical content of different species from the genus; (2) gather and compare the nutritional profiles of the various species and (3) investigate the physicochemical parameters associated with the genus.

## 2. Materials and Methods

### 2.1. Study Design

This review used the Preferred Reporting of Items for Systematic Reviews and Meta-analysis (PRISMA) [39] guidelines designed for various scientific fields, which identify, select, assess, and synthesize studies to demonstrate transparency, accuracy, and fidelity in data reporting [40,41]. The review protocol was not registered in any publicly accessible registry before it was conducted.

### 2.2. Eligibility Criteria

As part of this systematic review, only English-language outputs from the last 10 years (2014 to 2024) with open access were considered. Furthermore, all book chapters, conference papers, opinion articles, editorial material, review papers, and meta-analyses were excluded. Apart from the date, article typology, language, and access, articles were also included if they focused on the nutritional profile, physicochemical parameters, and chemical composition of the fruits or leaves from different species from the *Myrica* L. genus. The included terms were exclusively related to the *Myrica* L. genus, and historical nomenclatural changes and taxonomic reclassifications were not considered in the study. The studies were excluded when they portrayed themes that were off-topic or related to the chemical composition of essential oils, clinical trials that analyzed health effects, the chemical composition of by-products, method optimization, genomics, and papers in which the chemical composition was referenced in previous works. Eligibility and exclusion criteria (Table 1) were established before the literature search to minimize potential bias during the search process.

### 2.3. Literature Search

A simple literature analysis was performed to retrieve the search terms. The search was conducted within three databases, with the research expression (*Myrica* AND (Berr* OR Fruit* OR Leaves OR Leaf) AND (Physicochemical OR Phytochemical OR Chemical OR Biochemical OR Nutritional) AND (Composition OR Compound* OR Profile OR Value)) in Web of Science and PubMed and (*Myrica* AND (Berry OR Berries OR Fruit OR Fruits OR Leaves OR Leaf) AND (Physicochemical OR Phytochemical OR Chemical OR Biochemical OR Nutritional) AND (Composition OR Compound OR Compounds OR Profile OR Value)) in Scopus, on October 10, 2024. The terms in the Web of Science and Scopus databases were only searched in the title, abstract, keywords, and author keywords. With the use of Boolean operators and the asterisk, it was possible to focus the search on a specific subject. The AND connector narrows the search, retrieving all records with both words, the OR makes it possible to retain articles that include any of the words, and the asterisk makes it possible to retrieve all the items with words that share the same prefix. After searching for the research expression in the databases, filters regarding language, date, document type, and open access were applied under the eligibility criteria. The PubMed database did not enable the selection of only articles. Therefore, the exclusion of book chapters, conference papers, opinion articles, editorial material, review papers, and meta-analysis was conducted during the data collection phase.

### 2.4. Data Collection and Extraction

**Figure 1:**
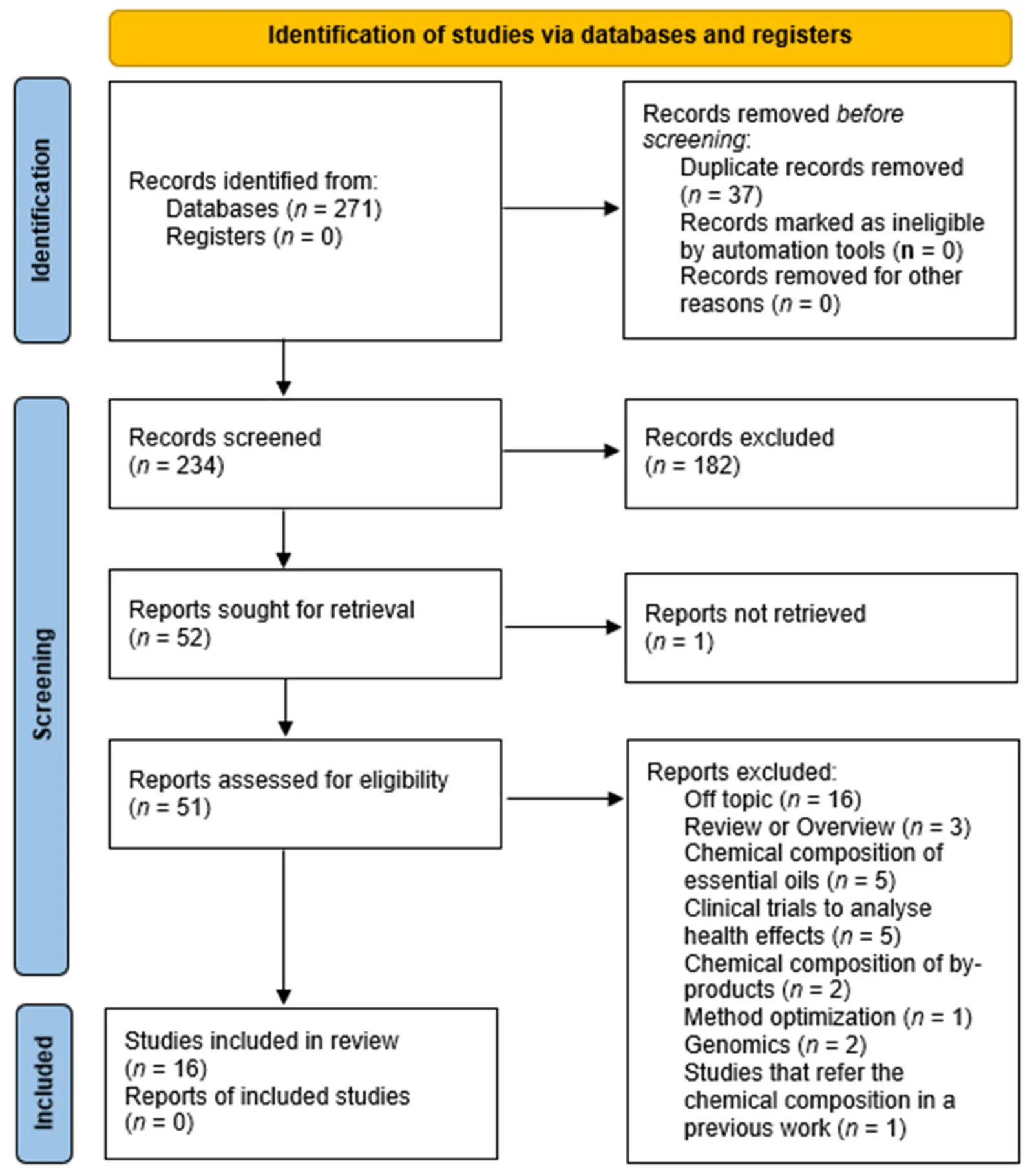
PRISMA flow chart for *Myrica* L. genus records found in the databases.

A total of two hundred and seventy-one records were identified through the database search, from which thirty-seven duplicated records were removed. After an initial screening, where all article titles and abstracts were read, one hundred and eighty-two records were excluded for not meeting the eligibility criteria. Of the remaining fifty-two records, fifty-one were assessed for eligibility; sixteen were included in the review [42,43,44,45,46,47,48,49,50,51,52,53,54,55,56,57] and thirty-five were judged ineligible when off-topic (16), a review/overview (3), or related to the themes of the chemical composition of essential oils (5), clinical trials that assessed health effects (5), the chemical composition of by-products (2), method optimization (1), genomics (2), and where the chemical composition was referenced in a previous work (1). This process was conducted in parallel by two of the reviewers, and any disagreements in the selection process were discussed and a consensus reached. The identification, screening, eligibility, and inclusion of the articles screened are reported in the PRISMA flow diagram (Figure 1). No additional search methods were conducted, such as reference-list screening or citation searching. The articles from the literature search were extracted to the EndNote^TM^ (Clarivate, Philadelphia, PA, USA) reference manager for screening. After the selection process was concluded, the full text of the included articles was assessed and screened. All the data that responded to the review’s aims were extracted by one author and reviewed by all three authors using a data collection spreadsheet. The information was then summarized into the tables presented in the review and in the Supplementary Materials. All the data retrieved were obtained exclusively from the published papers, ensuring impartiality and objectivity.

### 2.5. Data Analysis

The data were collected into a sheet, and some graphs and basic statistics—descriptive statistics as frequencies and percentages—were created using the Excel software (Microsoft Office Package, Microsoft Corporation, Redmond, DC, USA).

The VOSviewer 1.6.20 software (https://www.vosviewer.com/) (Centre for Science and Technology Studies, Leiden University, The Netherlands), a tool for building and visualizing co-authorships and co-occurrence networks, was used to conduct an exploratory bibliometric analysis, providing information regarding the relations among authors and between keywords. With the option “create a map based on bibliographic data”, a file was selected, which contained the metadata of the articles included in the current review. Two analyses were then conducted: a co-authorship analysis with links between authors and a co-occurrence analysis with the unit of analysis being the keywords. The analyses were conducted using the full counting method and the association strength normalization method was applied. No thesaurus file was applied during the analysis, as the data source consisted of the original metadata from the scientific databases.

### 2.6. Study-Quality Assessment

As there was no validated instrument for assessing the risk of bias of the included studies, the reviewers created an exploratory checklist tool to evaluate the methodological quality of the study. The tool was derived from questions obtained from the Meta-Analysis of Statistics Assessment and Review Instrument (MAStARI) [58], coupled with some questions from the work of Gadioli et al. (2018) [59] and some created specifically for this review by the authors. A total of nine exploratory questions resulted from the assessment tool created by the authors, namely, D1: Was the study based on a random sample?, D2: Was the sample identified and authenticated by a specialist?, D3: Was the general location of the sample disclosed?, D4: Was the exact location of the sample collection disclosed? D5: Was appropriate statistical analysis used?, D6: Does the article report the optimization of the extraction technique?, D7: Does the article explain the methodology for each of the experiments conducted?, D8: Is the article funded by an institution that would not influence the work? and D9: Is there no conflict of interest? Each question, for each study, was answered with either Yes, No, No information, or Unclear. Afterwards, the Yes answers were coded as High-quality while the No answers were coded as Low-quality.

The overall study quality was classified into exploratory categories, based on the “High-quality” answers as High if the answers were above or equal to 70%, moderate if they were between 50% and 69%, and Low if they were less or equal to 49%. The free web application, robvis [60] (University of Bristol, Bristol, United Kingdom), a visualization tool, was used to show the main results of the aforementioned assessment.

## 3. Results and Discussion

### 3.1. Bibliometric Analysis

An exploratory bibliometric analysis was performed for all the sixteen included articles using the VOSviewer software. This tool generates maps that visualize items or objects of interest, such as authors or keywords, as circles and the respective relations between them as lines. Apart from the items and the links connecting them, the software also groups the items into clusters depicted on the map with different colors. The size of the circles and corresponding labels are determined by the frequency with which the item appears, and the distance between two items indicates the relatedness between them.

**Figure 2:**
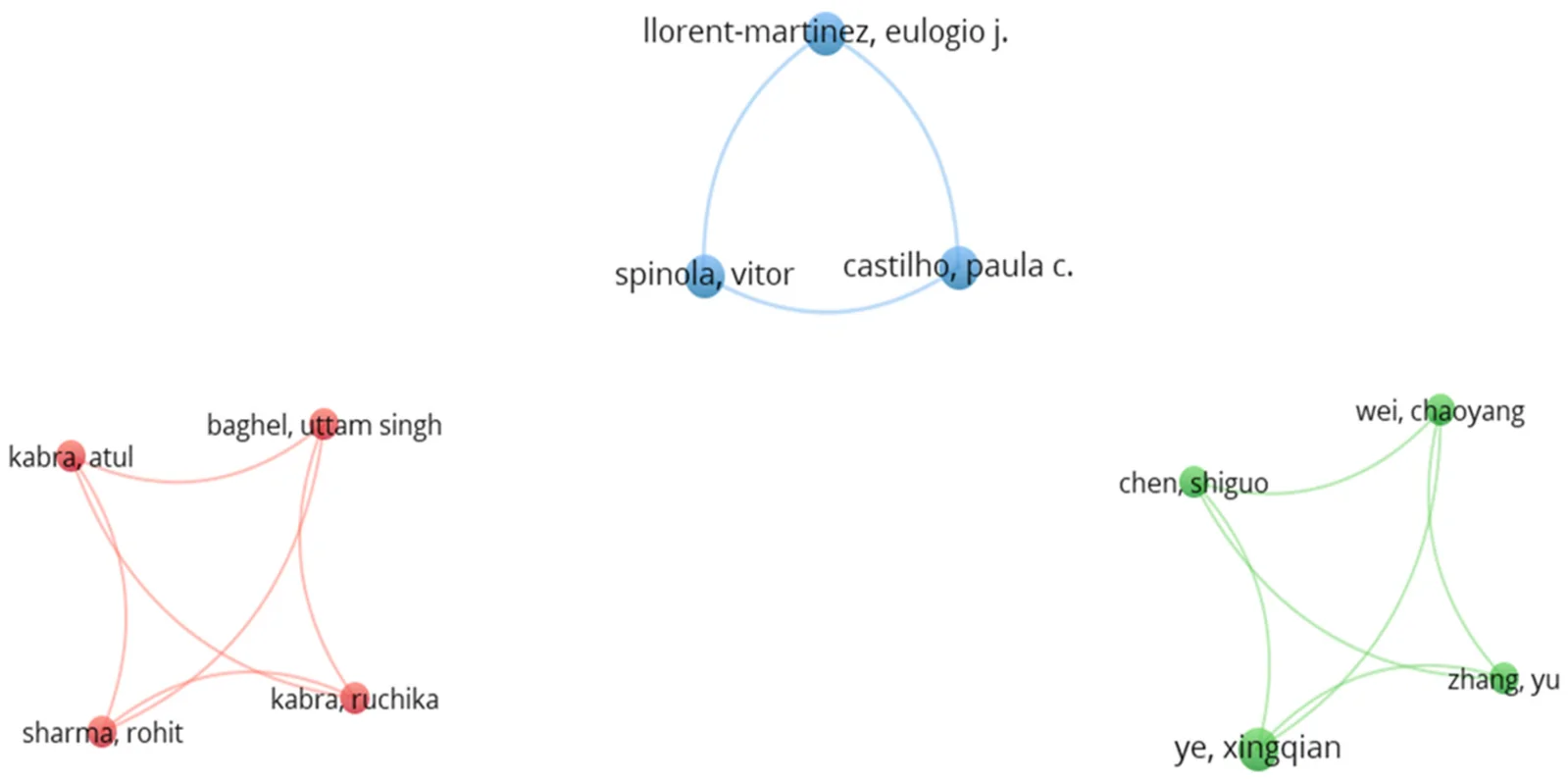
Analysis of co-authorship relations among authors, considering those that occurred a minimum of two times.

The co-authorship analysis of the relations among authors considered only those that occurred at least twice (Figure 2). Across the sixteen articles, a total of eighty-nine authors were identified, but only eleven met the threshold, creating a map with three clusters and fifteen relations between authors. The first and second clusters comprised four authors and the third cluster had three authors. The most frequently occurring authors were Castilho, Paula C., Lorrent-Martinez, Eulogio J., Spínola, Vitor and Ye, Xingqian. The map shows three clusters that highlight three distinct teams that work with different species of the *Myrica* L. genus. Whereas the authors in the blue cluster investigate the species *Myrica faya* Aiton, the authors from the red cluster focus on the *Myrica esculenta* Buch.-Ham. ex D. Don species, and the authors belonging to the green cluster research the species *Myrica rubra* Sieb. & Zucc.

**Figure 3:**
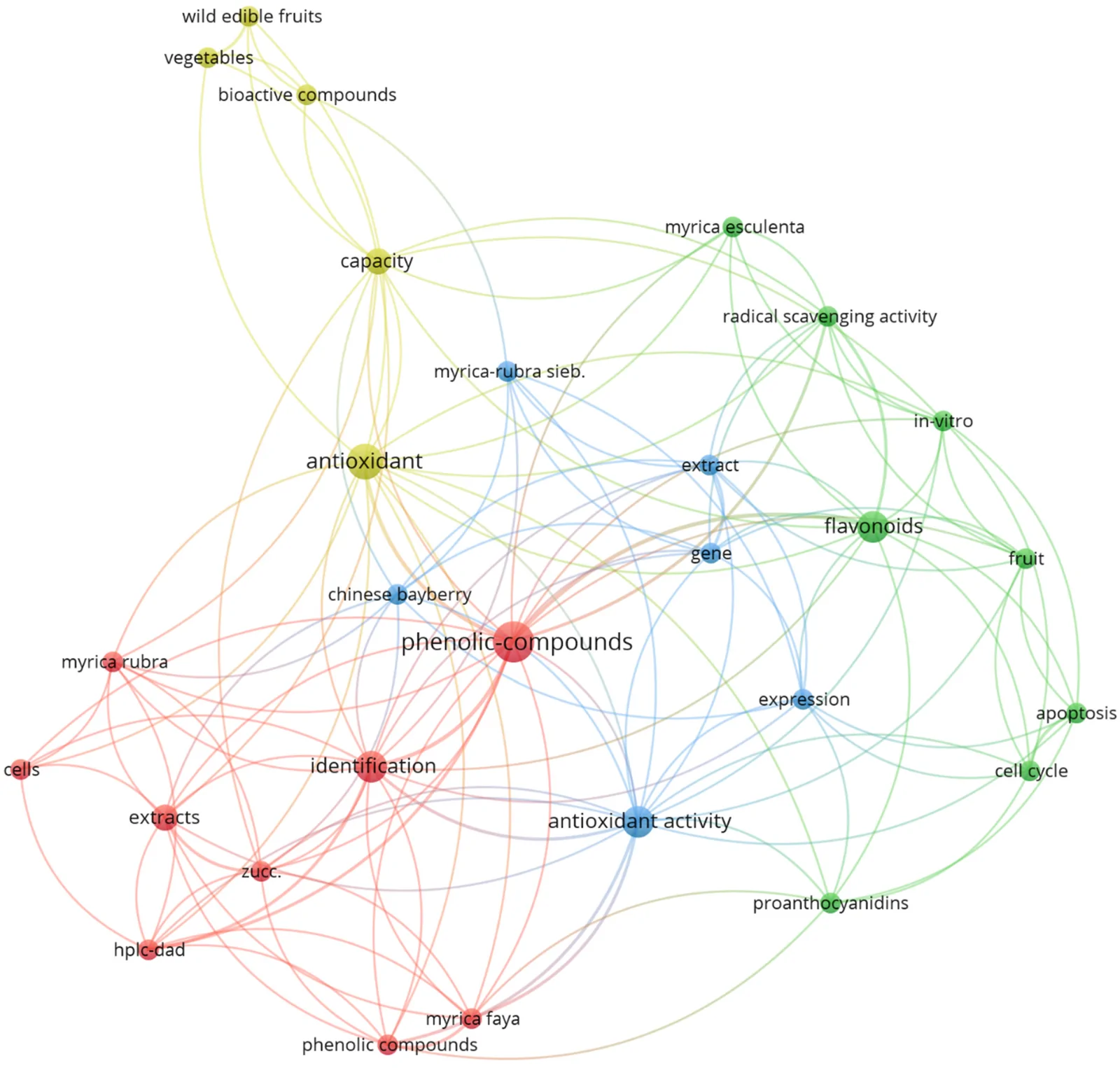
Analysis of co-occurrence associations between keywords, considering those that occurred a minimum of two times.

The analysis of co-occurrence associations between keywords only considering those that occurred a minimum of two times (Figure 3). Out of the one hundred and forty-eight keywords present in the bibliographic metadata, twenty-eight met the threshold, creating four clusters and a total of one hundred and thirty-seven links. The first cluster contained nine items, the second cluster eight items, the third six items, and the fourth cluster five items. The map created by the software (Figure 3) highlights two limitations that the researchers were unable to solve since the bibliographic data are run as a single unchangeable metadata file. The first limitation relates to the item phenolic compounds, which appears two separate times due to a difference in the spelling of the word: one form is hyphenated and the other is not. The second limitation concerns the term zucc., which appears in the map as a separate keyword rather than being grouped with the term *Myrica rubra* Sieb., as part of the correct terminology, *Myrica rubra* Sieb. & Zucc. Apart from the unchangeable limitations, the most frequent keywords shown in Figure 3 were phenolic compounds, antioxidant activity, identification, antioxidant, and flavonoids. Additionally, three species of *Myrica* L. also appeared, namely, *Myrica faya* Aiton, *Myrica esculenta* Buch.-Ham. ex D. Don and *Myrica rubra* Sieb. & Zucc, the latest with both the scientific name and the common name, Chinese bayberry.

This bibliometric analysis revealed several gaps in knowledge. For example, the author network analysis indicates that *Myrica nagi* Thunb. has been considerably less studied than the other species examined. In contrast, *Myrica rubra* Sieb. & Zucc and *Myrica esculenta* Buch.-Ham. ex D. Don display a higher number of connections within the network, indicating greater research attention, while *Myrica faya* Aiton has been less explored. The keyword analysis also highlights topics that have received less attention in the available literature, particularly in areas related to gene expression, genotype characterization, nutritional composition and physicochemical characterization.

### 3.2. Research Characteristics

**Figure 4:**
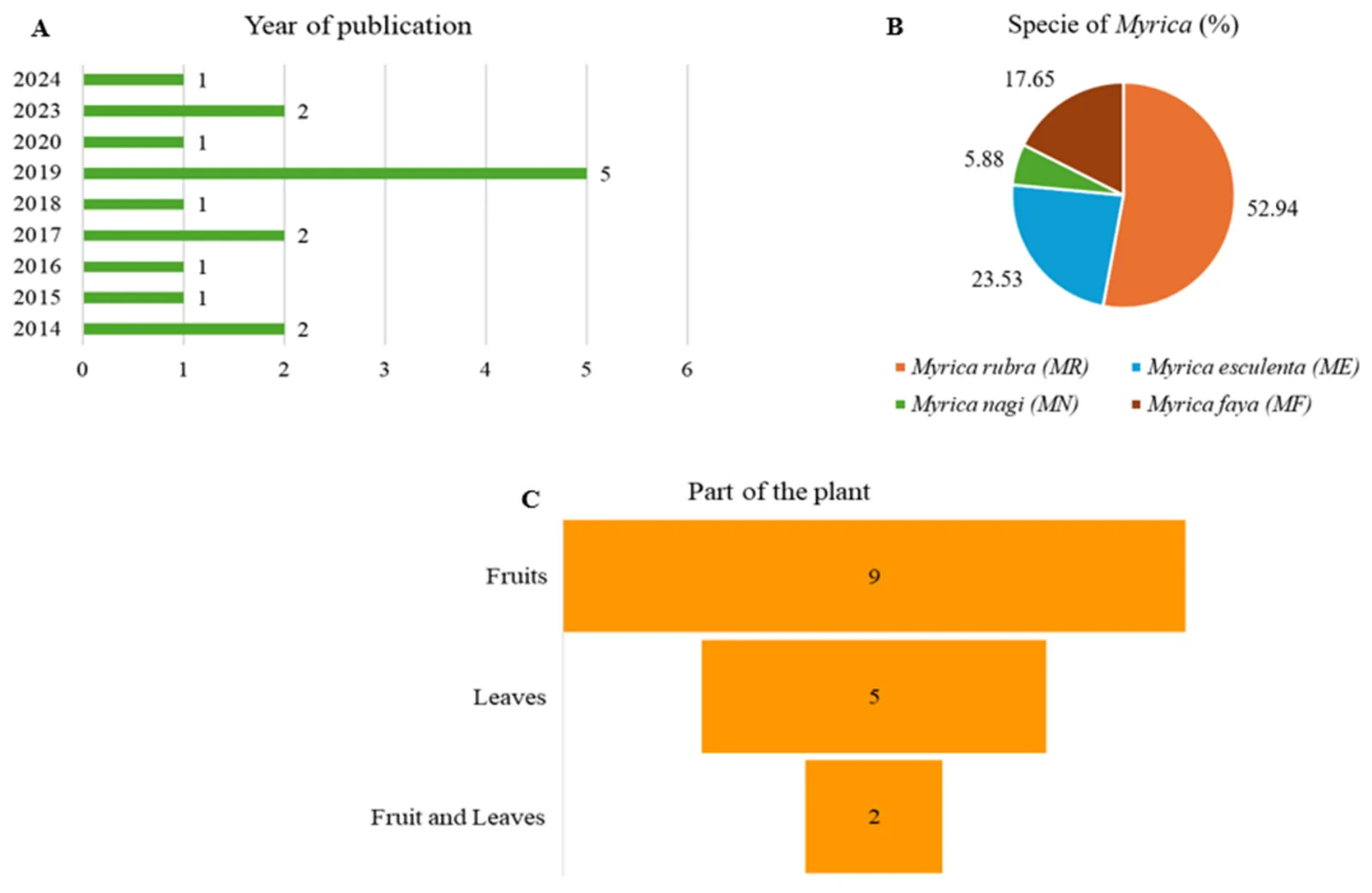
(**A**) Number of articles by year of publication; (**B**) percentage of articles according to species of *Myrica* L. and (**C**) number of articles according to the part of the plant analyzed.

Regarding the research characteristics, Figure 4A, Figure 4B and Figure 4C depict, respectively: the number of articles by year of publication; the percentage of articles according to species of *Myrica* L.; and the number of articles according to the part of the plant analyzed. These percentages were calculated based on the occurrence of each category within the included studies, considering that some articles reported more than one *Myrica* L. species or part of the plant.

In this review, sixteen articles were included from 2014 onwards, as described in the inclusion criteria. Of these papers, the year 2019 was the year with the most published papers, with a total of five, followed by 2014, 2017, and 2023, with two articles published, and 2015, 2016, 2018, 2020, and 2024, with only one research published in each of the respective years, as presented in Figure 4A. These results have show that at least one publication on the topic appeared each year between 2014 and 2024, except for the period 2021–2022, in which there were no publications. Regarding the species of *Myrica* L. portrayed in the included studies (Figure 4B), *Myrica rubra* Sieb. & Zucc (MR) appeared in 52.94% of the articles [42,45,46,47,49,52,55,56,57], followed by *Myrica esculenta* Buch.-Ham. ex D. Don (ME) (23.53%) [43,44,51,53], *Myrica faya* Aiton (MF) (17.65%) [48,50,54], and *Myrica nagi* Thunb. (MN) (5.88%) [44]. Therefore, the statistics indicate that MR is the most extensively studied species in the *Myrica* L.. The included articles focused on two parts of the plant: the fruit and leaves. The fruit was the most analyzed part, being examined in eleven of the studies [42,43,44,46,47,48,49,50,52,54,57], whereas leaves were investigated in seven of the articles [45,50,51,53,54,55,56] (Figure 4C). In two of the articles [50,54], both the fruits and the leaves were studied, while the other fourteen papers, by contrast, only studied one of the two parts.

With respect to the countries where the studies were conducted, eight of the studies were conducted in China [42,45,46,49,52,55,56,57]; four in India [43,44,51,53], three in Portugal [48,50,54], and one study did not report the country or sample location [47].

**Figure 5:**
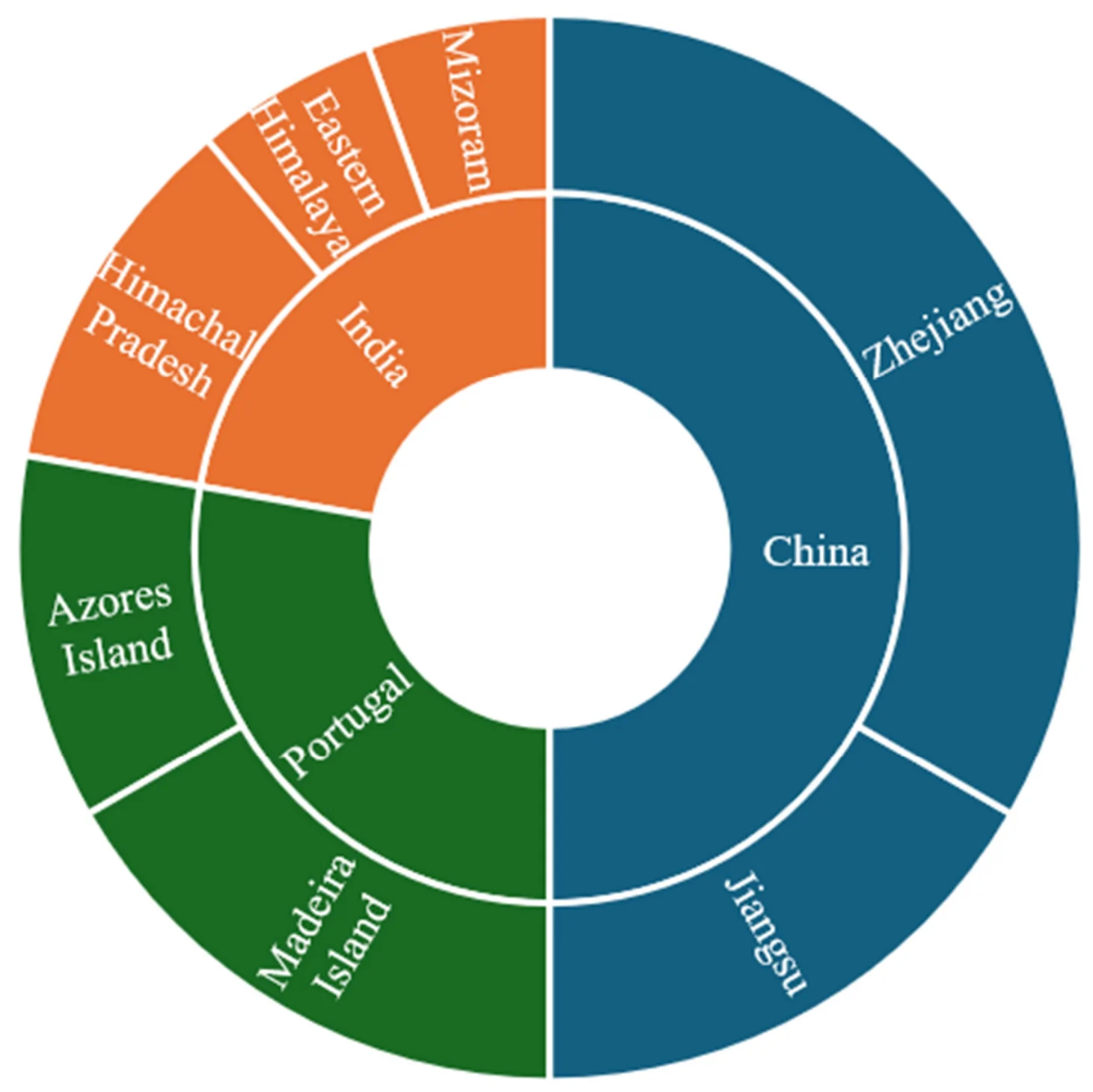
Geographical distribution by frequency of sample collection.

The various *Myrica* L. specimens were harvested from different locations, as depicted in Figure 5. In six cases, the samples were collected in Zhejiang Province, China [42,45,49,52,55,56]; in three cases from Jiangsu Province, China [46,47,57]; in three from Madeira Island, Portugal [48,50,54]; in two from Himachal Pradesh, India [51,53]; in two from the Azores Island, Portugal [48,54]; in one from Mizoram, India [43]; in one from Eastern Himalaya, India [44]; and in one of the studies [47], the place where the sample was collected is not mentioned.

### 3.3. Study-Quality

**Figure 6:**
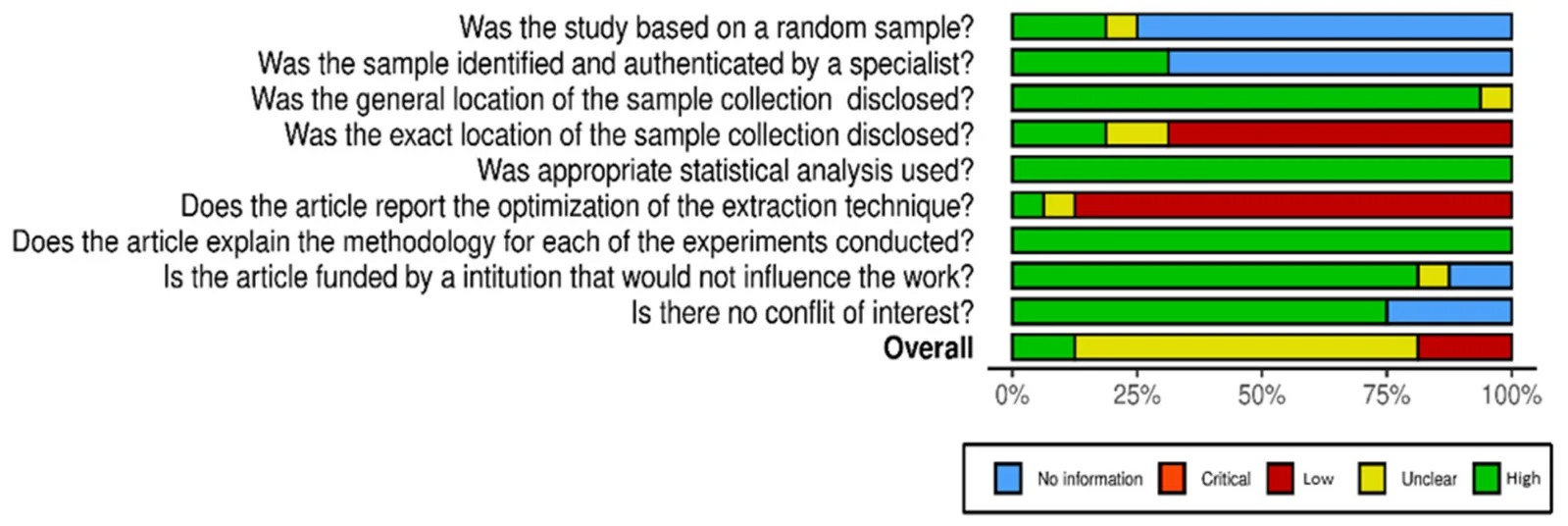
Study-quality assessment graph using the robvis tool.

**Figure 7:**
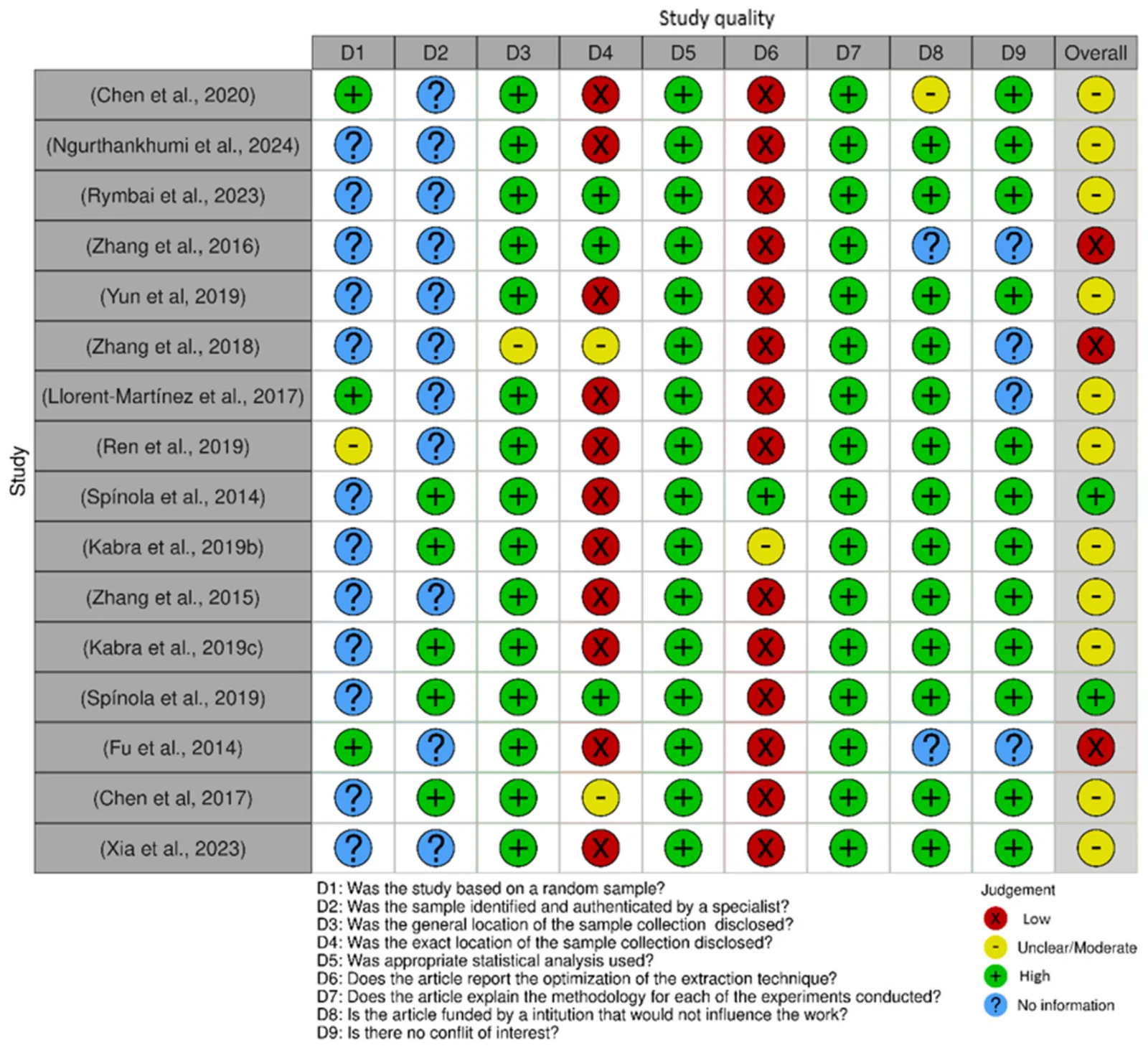
Study-quality assessment summary using the robvis tool [42,43,44,45,46,47,48,49,50,51,52,53,54,55,56,57].

The study-quality assessment was performed for the sixteen included studies; the main results are presented in Figure 6 and Figure 7. Among the assessed articles, two of the included studies presented a high-study quality [50,54], eleven of the articles presented a moderate-study quality [42,43,44,46,48,49,51,52,53,56,57], and three presented a low methodological quality [45,47,55].

A total of nine exploratory questions (see Figure 7) were asked to assess the quality of the studies, of which four raised concerns regarding information not provided or the low study quality, namely, D1: Was the study based on a random sample?, D2: Was the sample identified and authenticated by a specialist?, D4: Was the exact location of the sample collection disclosed? and D6: Does the article report the optimization of the extraction technique? These findings suggest that future studies should include clear, precise, and detailed information regarding the randomization, detailed location, identification, and authentication of the sample, as well as the optimization of the extraction technique.

### 3.4. Physicochemical, Nutritional, and Phytochemical Composition of the Fruits

The reported data should be interpreted in the context of the differences among studies and should not necessarily reflect inherent species-level differences.

#### 3.4.1. Physicochemical Parameters

In this review, the included articles analyzed physicochemical parameters such as moisture, weight, edible rate, titratable acidity, pH, color, fruit length, diameter, circumference, volume, and seed weight, length, breadth, and number (Table 2, Table 3, Table 4 and Table 5). The Supplementary Materials include further information, specified by species, sub-species or variety for moisture, titratable acidity, weight, pH, edible rate (Supplementary Table S1), as well as information regarding other parameters assessed, such as dry matter, juice and pulp content, total ash (Supplementary Table S2), and color (Supplementary Table S3).

The moisture content, as shown in Table 2, for the *Myrica* L. species, is 83.62 ± 1.52% for MN and ranges between 86.75% and 89.00% for ME [43,44]. These values are in the same range as other red fruits, such as sweet cherries, whose moisture values range between 75.08 ± 1.34% and 88.56 ± 1.72% [61,62]; blackberries, with a moisture content of 87.1% [63]; and raspberries, with a figure of 88.2% [63].

Regarding fruit mass (Table 2), the studies indicated that the weight varies between 8.30 and 28.17 g. More specifically, for ME and MN, the mean weights are 13.26 ± 1.83 g and 8.30 ± 0.56 g, respectively [44], and for MR the weight ranges from 8.8 to 28.17 g. The reported values highlight the greater mass of these fruits in comparison with, for example, raspberries, whose weight ranges from 1.47 to 2.32 g [64], or goldenberries, ranging from 2.734 to 3.707 g [65].

The edible portion, meaning the part of the fruit that it is eaten, was one of the parameters assessed to characterize MR (Table 2). The study by Zhang et al. (2015) [52] revealed that the edible rate—weight percentage of pulp to the fruit—of MR ranges from 94.30% to 97.39%. The edible portions of other fruits, such as cherries, redcurrants, and plums, have similar values: 82%, 99%, and 97%, respectively [63].

Regarding acidity (Table 2), two articles [43,44] measured the titratable acidity of ME and MN. While for ME, the titratable acidity ranges from 1.38% to 3.32%, the mean titratable acidity of MN is 2.45 ± 0.04%. These values indicate a higher titratable acidity when compared to raspberries [64], with acidity ranging from 1.26% to 1.82%. In contrast, ME and MN have a lower acidity percentage than the sea buckthorn species, which has a titratable acidity ranging from 3.14% to 4.73% [66].

Furthermore, pH (Table 2) was also evaluated for MR [42] and MF [50]. While the pH of MR varies from 2.63 to 2.92, which is lower than that of goldenberries 6.07 [65] or cherries (3.49 to 4.11) [61], the pH of MF is 4.02, which is higher than that of sea buckthorn (2.85 to 3.01) [67].

Regarding fruit length, diameter, circumference, and volume (Table 3), results have shown that ME has a bigger fruit compared with MN. Whereas the length, diameter, circumference, and volume of ME are 3.14 ± 0.15 cm, 2.73 ± 0.11 cm, 8.13 ± 0.31 cm and 14.64 ± 1.76 cm^3^, respectively [44], for MN they are 1.52 ± 0.05 cm, 1.23 ± 0.08 cm, 3.64 ± 0.24 cm and 9.07 ± 0.70 cm^3^, correspondingly [44]. MR fruit has a greater length, diameter, and volume than goldenberries [65], which, on average, have a length of 1.75 cm, diameter of 1.731 cm, and volume of 2.72 cm^3^. Apart from the fruit, measures were also taken for the seeds (Table 4). In correlation to the measurements performed for the fruit, it was also verified that the seeds, only one per fruit, from ME are greater in length and breadth than the seed from MN. Whereas the seed length and breadth of ME [44] are 19.62 ± 0.18 mm and 13.74 ± 0.03 mm, respectively, the seed length of MN [44] is 10.03 ± 0.05 mm, and the seed breath is 0.82 ± 0.04 mm, as reported in the original reference. Regarding the weight of the seed, the same trend remains, where the seed of ME weighs more (2.03 ± 0.06 g) than the one from MN (1.32 ± 0.06). The seed weights of both ME and MN are considerably greater than the weight of the cherry pit, which is 0.27 g to 0.39 g [62].

Color is not only a crucial indicator of fruit maturity, quality, and ripeness but also an important visual parameter influencing consumers’ choice and acceptance [68,69]. This review analyzed color for three species of *Myrica* L., MR, ME, and MN, all harvested at a mature stage (Table 5). The color of MR fruits at commercial maturity [52] was measured using the color index for red grapes (CIRG) [70], with values ranging from 1.96 to 12.39, which, as reported by Fernández-López et al. (1998) [71], represent external colors between green-yellow to blue-black. For the color measurement of the ME [44] and MN [44] species, the CIELAB system [72] was used. The L* represents brightness, which varies between 0 and 100, representing the colors from black to white, respectively. The variable a* characterizes redness, with values that range from negative to positive, representing green and red, respectively. Furthermore, yellowness is described by the b* variable, which represents blue if the values are negative and yellow for positive values [73]. The L* values were 54.2 ± 6.36 for ME and 25.6 ± 1.57 for MN; the a* values were 15.7 ± 4.29 for ME and 28.5 ± 1.51 for MN; and the b* values were 29.6 ± 2.05 for ME and 9.3 ± 0.67 for MN. These values translate into a brownish color for ME fruits and a reddish-burgundy color for MN fruits.

Several physicochemical parameters were evaluated to characterize the four species of *Myrica* L. portrayed in this review, namely, MR, ME, MN, and MF. The main research findings indicate that, overall, MR fruits have a larger weight range than ME and MN fruits and a lower pH than MF. Of the four species analyzed, MR was also the only one for which the edible rate was calculated. Regarding ME, when compared with MN, this species fruit is larger and has a higher moisture content.

#### 3.4.2. Nutritional Composition

The nutritional and overall quality of fruit is influenced by numerous factors, some related to pre-harvest conditions, such as light exposition, radiation, temperature, season, climatic environment, genotype, agronomic techniques air composition, irrigation, harvesting and soil quality, and others relating to the post-harvest stage, for example storage and processing [74,75,76].

Concerning the nutritional and basic chemical composition of the included articles, five [42,43,44,50,52] presented results relating to energy, carbohydrates, starch, protein, lipids, crude fiber, total sugar, sucrose, fructose, glucose, total soluble solids, ascorbic acid, citric acid and malic acid content (Table 6, Table 7 and Table 8). The fiber content, namely, lignin, cellulose and hemicellulose, as well as reducing sugar, non-reducing sugar, L-ascorbic acid, vitamin E, and mineral content, are expressed in Tables S4–S7 (Supplementary Materials).

Only one of the studies describes the nutritional composition of ME, based on a dried powder sample (Table 6). The values were reported directly from the original source and are presented for reference. As the described macronutrients, collectively, exceed 100%, these data should be interpreted with caution. Concerning the macronutrients, ME fruits have a lower percentage of carbohydrates (79.84%) compared with raspberries, *Rubus idaeus* [77], which have 86.6% dry matter. The fruits from ME also have 25.02% of protein, which is a considerably higher value than the protein content of raspberries (*Rubus idaeus*) (8.8% dry matter) [77]. As for lipid content, ME has similar values to those of other types of berries, with values of 1.64% for ME and 2.4% of dry matter for raspberries (*Rubus idaeus*) [77]. According to the data, ME berries [43] contain 434 kcal, which is associated with their high reported carbohydrate and protein content, as well as their lipid content.

Regarding sugar content (Table 7 and Supplementary Table S5), the sucrose content varies from 40.00 to 65.85 mg/g fresh weight (FW) for MR [42,52]; the fructose content ranges between 5.00 and 14.66 mg/g FW; and the glucose values are between 5.00 and 12.33 mg/g FW. In comparison, the sucrose content of MR is considerably higher than that of raspberries (*Rubus idaeus*) (6.9 to 9.1 mg/g FW) [77], whereas for fructose and glucose, the MR content is lower than the values presented for raspberries (*Rubus idaeus*) (31.5 to 32.2 mg/g FW for fructose and 24.3 to 38.2 mg/g FW for glucose) [77]. As for total soluble solids (Table 7), of the different *Myrica* L. species, MF [50] has the highest content, at 14.87 ºBrix, approximately double the value reported by Basak et al. (2022) [78] for strawberries (7.43 ºBrix).

The organic acid content (Table 8 and Supplementary Table S6) was one of the parameters assessed in four [42,43,44,52] of the included articles. The results show that MR [42,52] has the lowest ascorbic acid content, ranging between 1.79 and 4.20 mg/100 g, and ME [43,44] has the highest, ranging from 22.50 to 60.68 mg/100 g. The raspberry (*Rubus idaeus*) has a higher ascorbic acid content (92.2 mg/100 g dry matter) [77] than any of the *Myrica* L. species analyzed in the included studies.

Regarding MR citric acid, the content varies between 7.84 and 22.06 mg/g FW [42,52], a wider range than that found for blueberries (1.862 and 13.424 mg/g FW) [79]. Moreover, while malic acid values from MR range from 0.12 to 1.16 mg/g FW [42,52], the content in blueberries varies between 0.157 and 0.477 mg/g FW [79].

In the case of the fruit’s mineral content (Table S7 from the Supplementary Materials), a total of seventeen minerals were identified and quantified for MF, with potassium and sodium being the minerals with the highest content, 4400 ± 500 µg/g and 2000 ± 400 µg/g, respectively. ME’s mineral content was also assessed, with a total of eleven minerals quantified.

In summary, ME berries are a source of macronutrients, particularly carbohydrates and proteins, as well as micronutrients. Additionally, ME has the highest ascorbic acid content, followed by MN and MR. As for MR, the results have shown that this species has a higher sucrose content and less fructose and glucose than raspberries.

#### 3.4.3. Chemical Composition

The chemical composition of fruits can vary according to species, sub-species, variety, and growing conditions, such as location, soil, water, and air quality, as well as fruit maturity, harvesting, and storage conditions [80].

In this review, nine [42,43,44,46,49,50,52,54,57] of the studies assessed polyphenols and anti-nutrient content for four species of *Myrica* L., MR, ME, MN, and MF (Table 9, Table 10, Table 11, Table 12 and Table 13 and Supplementary Tables S8–S29). The contents of total phenolics, flavonoids, flavonols, individual phenolics, phenolic acids, flavanols, flavones, anthocyanins, ellagitannins, hydroxycinnamic acids, hydroxybenzoic acids, and carotenoids content are shown in Supplementary Tables S8–S11, with information regarding sub-species, variety, location, or maturation stage. Moreover, a total of forty-one polyphenols were identified and seventy-five quantified. The compounds’ identification and contents are expressed in Supplementary Tables S12–S29.

Regarding the total phenolic content (Table 9 and Supplementary Table S8), ME and MN have been shown to have a higher content, ranging between 10.00 and 15.00 mg gallic acid equivalent (GAE)/g [44], than MR, which has a content between 1.31 and 4.90 mg GAE/g [52,57]. These ME and MN values are also considerably higher than those presented for other fruits, for example, blackberries, raspberries [81], goldenberries [65], and cherries [63], which have values of 1.81 mg AE/g, 1.52 mg GAE/g, 1.45 mg GAE/g and 0.70 mg GAE/g, respectively.

The total flavonoid content (Table 9 and Supplementary Table S8) varies both between *Myrica* L. species and within the same species. In the MR case, one of the articles [52] has shown that the total flavonoid content ranges from 0.85 to 1.91 mg rutin equivalent (RE)/g. These values are considerably lower compared to the ones presented in the article by Orsavová et al. (2023) [82], which describes values between 15.04 and 26.85 mg RE/g for sweet rowanberry cultivars. In contrast, the flavonoid content of ME and MN [44], which ranges from 4.50 to 6.00 mg quercetin equivalents (QE)/g and 3.00 to 4.50 mg QE/g, respectively, are higher than the values reported by Subbiah et al. (2020) [81] for blackberries (0.03 mg QE/g), raspberries (0.02 mg QE/g), and strawberries (0.01 mg QE/g).

The total flavonol content is only presented in one of the studies [44] for both ME and MN, with values between 1.00 and 2.00 mg QE/g (Table 9 and Supplementary Table S8). In total, twenty-seven flavonols were quantified (myricetin, myricetin-*O*-hexoside, myricetin-*O*-deoxyhexoside, myricetin-*O*-(*O*-galloyl)deoxyhexoside, myricetin-*O*-(galloyl)hexoside, dimethyl-myricetin-*O*-pentoside, myricetin-*O*-(galloyl)deoxyhexoside, myricitrin, myricetin-*O*-pentoside, myricetin-3-*O*-rhamnoside, myricetin deoxyhexoside-gallate, kaempferol-*O*-hexoside, kaempferol-3-*O*-galactoside, kaempferol-3-*O*-glucoside, kaempferol, dihydro-kaempferol-*O*-hexoside, kaempferol-*O*-(galloyl)hexoside, ellagic acid-*O*-pentoside, quercetin-3-*O*-galactoside, quercetin-3-*O*-glucoside, quercetin-3-*O*-rhamnoside, quercetin-*O*-hexoside, quercetin, quercetin-*O*-deoxyhexoside, quercetin-*O*-(galloyl)hexoside, quercetin-*O*-(galloyl)deoxyhexoside and galloylquercetin-*O*-hexoside) (Supplementary Tables S16–S20). Two, myricetin-*O*-(*O*-galloyl) hexoside and kaempferol-*O*-rhamnoside, were analyzed but not detected in the samples (Supplementary Tables S16 and S18).

**Figure 8:**
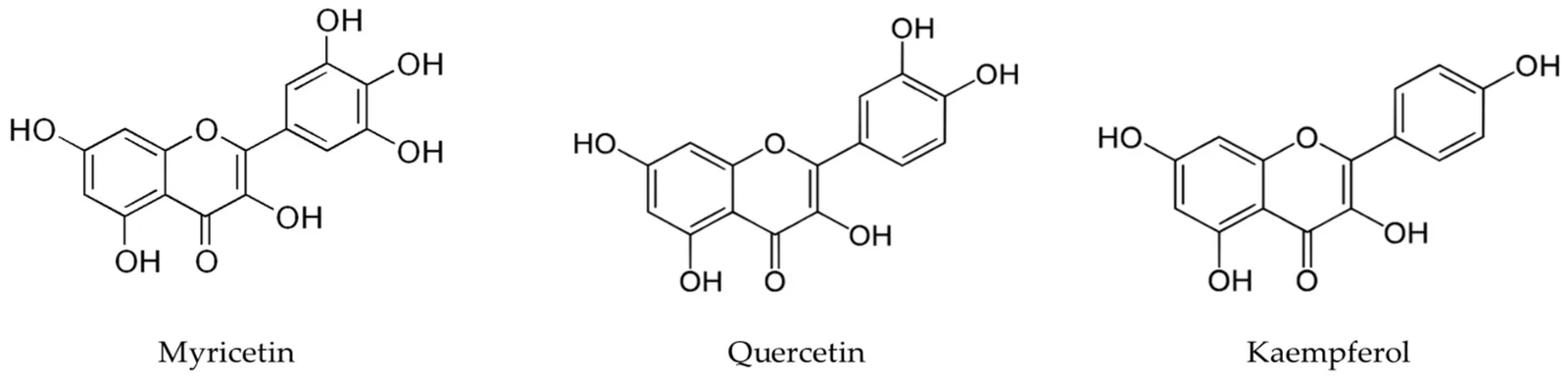
Myricetin, quercetin and kaempferol molecular structure.

Myricetin (Figure 8) is a flavonol present in numerous plant species from the *Myricaceae*, *Polygonaceae*, *Primulaceae*, *Pinaceae*, and *Anacardiaceae* families, being prevalent in fruits, vegetables, teas, and wine. This compound was first extracted from the MN plant. Since then, its use has been found to have multiple health benefits, such as inhibition of hyperglycemia, antioxidant properties, cholesterol reduction and liver protection [83,84]. In this review, myricetin and several derivates have been quantified for MR and MF species (Supplementary Tables S16 and S17). The myricetin content of MR [42,57] ranges from 0.002 to 1.27 mg/g, which is considerably lower than the content of blackberries (7 mg/g), blueberries (13 mg/g), and cranberries, which is 66 mg/g [83]. Table S16 from the Supplementary Materials shows that the maturation stage of three MR varieties influences the myricetin content. For the DongKui variety, there is a slight decrease from veraison (0.07 mg/g) to maturity (0.06 mg/g). In contrast, the Muye variety shows an increase from veraison (0.26 mg/g) to maturity (1.27 mg/g). Similarly, the myricetin content of the ZaoJia variety increases from veraison (0.10 mg/g) to maturity (0.88 mg/g) [42].

Quercetin and kaempferol, along with myricetin (Figure 8), are flavonols present in fruit and vegetables that differ in terms of their chemical structures by a single hydroxyl group; specifically, myricetin has an extra OH group at the fifth position of the B ring compared with quercetin, and quercetin has an extra OH group at the third position of the B ring compared with kaempferol [85]. The MR quercetin content [42,57] varies between 0.00079 mg/g and 13.89 mg/g, and the kaempferol content [57] is 0.87 µg/g FW (Supplementary Tables S18 and S20). The study by Dabeek et al. (2019) [85] showed that the quercetin content of cherries and cranberries is 0.17 mg/g FW and 0.25 mg/g FW, respectively, whereas for kaempferol these values are 51.4 µg/g FW for cherries and 2.1 µg/g FW for cranberries.

The total content of individual phenolics, total phenolic acids, total flavonols, total flavanols and total flavones have been assessed in two studies by Spínola et al. (2014) [50] and (2019) [54] (Table 10 and Table S9 in the Supplementary Materials). Although the same species is the same, the values cannot be compared since they are expressed in different units. Regarding total phenolic acids content, the MF value, 45.73 ± 0.97 mg/100 g dry weight (DW) [50], is higher than that for Prunus spinosa [86], with a content of 29.78 mg/100 g.

Regarding flavanols, in total, seven compounds (gallo(epi)catechin, gallocatechin dimer, digalloyl(epi)gallocatechin dimer, (+)catechin, (epi)catechin-*O*-gallate, gallo(epi)catechin-*O*-gallate and digallocatechin derivative) were quantified, and one, (epi)catechin, was analyzed but not detected (Supplementary Tables S21 and S22).

The flavones tricin-*O*-hexoside, a tricin-*O*-hexoside derivative, and a luteolin-*O*-hexoside derivative were also analyzed and quantified for MF (Supplementary Table S23).

**Figure 9:**
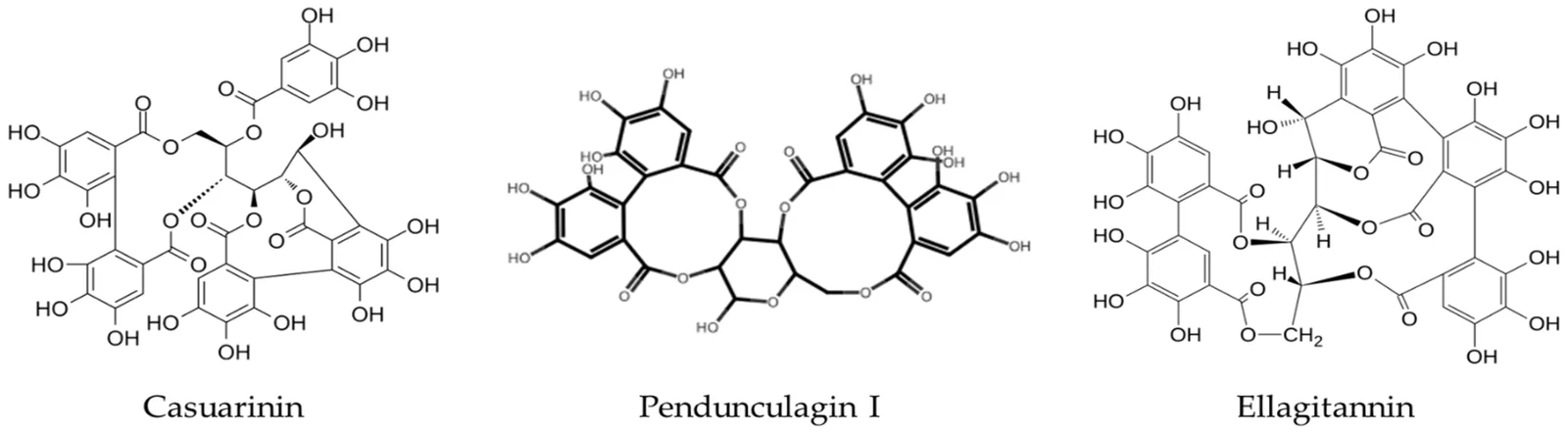
Casuarinin, pendunculagin I and ellagitannin molecular structure.

Supplementary Table S24 depicts the five ellagitannins quantified (pedunculagin I, casuarinin, ellagitannin, hexahydroxydiphenoyl-*O*-hexoside and ellagic acid-O-pentoside) (Figure 9).

Phenolic acids are polyphenols that consist of two classes, hydroxycinnamic acid and hydroxybenzoic acid [87]. In this review, eight hydroxycinnamic acids (caffeoylisocitrate, 5-*O*-caffeoylquininc acid, caffeic acid-*O*-hexoside, dihydro-Co-*O*-hexoside, benzoyl-p-tri-coumaroyl-2,7-anhydro-3-deoxy-2-octulopyranosonic acid, coumaric acid-*O*-hexoside, p-coumaric acid and ferulic acid) and eight hydroxybenzoic acids (galloyl-*O*-hexoside, galloylquinic acid, digalloyl-*O*-hexoside, trigalloyl glucose, methyl gallate derivative, gallic acid, p-hydroxybenzoic acid and protocatechuic acid) were quantified (Supplementary Tables S12–S15).

**Figure 10:**
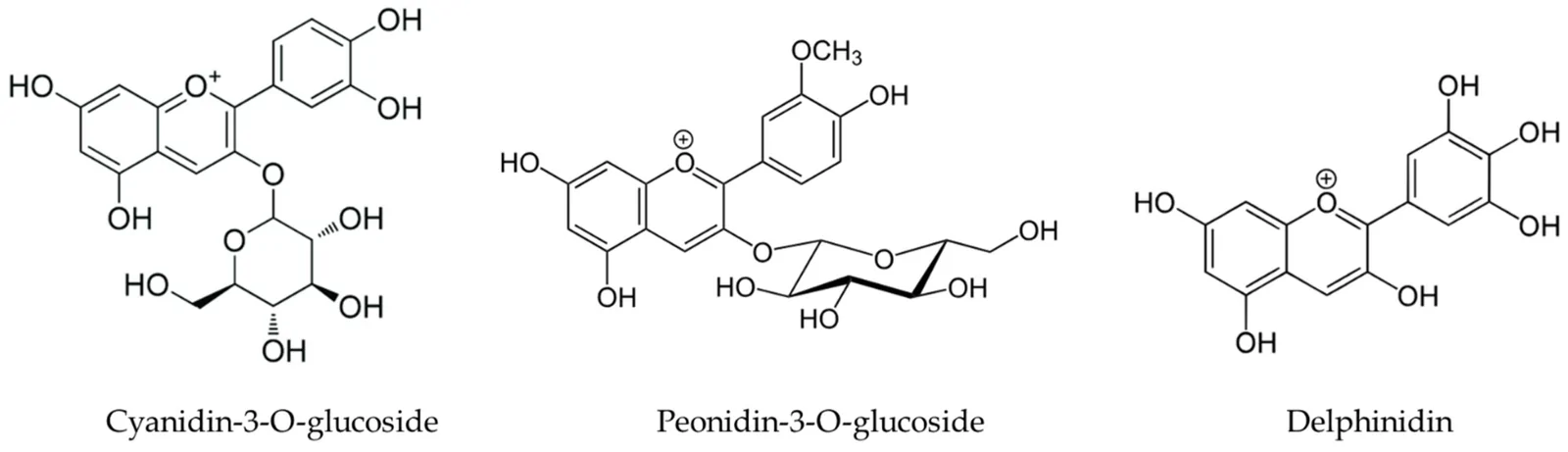
Cyanidin-3-*O*-glucoside, peonidin-3-*O*-glucoside and delphinidin molecular structure.

Table 11 and Supplementary Table S10 describe the total content of anthocyanins and carotenoids for MR, ME and MN. The data obtained from the table demonstrate a large variation in the anthocyanins content [42,43,44,46], ranging between 0.17 mg/g and 273 mg/g, for the three species analyzed. Other berries have presented values between 22 mg/g for redcurrant and 300 mg/g for bilberry [88]. In total, thirteen anthocyanins (cyanidin-3-*O*-glucoside, cyanidin-3-*O*-galactoside, cyanidin-*O*-hexoside, cyanidin, cyanidin-*O*-pentoside, cyanidin-*O*-(acetyl)hexoside, peonidin-3-*O*-glucoside, peonidin, delphinidin-*O*-hexoside, delphinidin-hexoside, delphinidin, pelargonidin and pelargonidin-3-*O*-glucoside) were quantified (Supplementary Tables S25–S27) (Figure 10).

Cyanidin-3-*O*-glucoside is an anthocyanin that confers a red color on fruits. For MR [52], the content of this anthocyanin varies between 9.34 and 912.24 µg/g FW (Supplementary Table S25). This content falls within the same range of values as the values reported for raspberry (148.9 µg/g FW) and redcurrants (33.7 µg/g FW) [89]. Apart from cyanidin-3-*O*-glucoside, other anthocyanins were also quantified, for example, delphinidin (Supplementary Table S27), a compound responsible for conferring a purple-to-magenta color on fruits and vegetables, as well as being a promising treatment for some types of cancer [90]. When comparing delphinidin content, MR values [56] range from 6.00 to 10.00 ng/g and are significantly lower than the ones for more purply fruits, such as blueberries (14,530 ng/g to 1,013,490 ng/g) [91].

Regarding total carotenoid content (Table 11 and Supplementary Table S10), the values vary between 5.00 and 12.00 mg/g for MR [42], between 0.01 and 0.12 mg/g for ME [43,44], and between 0.12 and 0.14 mg/g for MN [44]. These values, especially the ones from MR, are notably higher than the total carotenoid content described by Toledo-Martín et al. (2018) [92] for blackberries (0.003 mg/g to 0.01 mg/g). Table S10, from the Supplementary Materials, shows that the maturation stage decreased the total carotenoid content in the DongKui and *Muye varieties*. For the DongKui variety, there is a decrease from veraison (7.00–8.00 mg/g) to maturity (5.00–7.00 mg/g). Similarly, the Muye variety also shows a decrease from veraison (8.00–10.00 mg/g) to maturity (5.00–7.00 mg/g). In contrast, the total carotenoid content of the ZaoJia variety remained constant from veraison (10.00–12.00 mg/g) to maturity (10.00–12.00 mg/g) [42] Carotenoids are components found in fruits and vegetables that confer a yellow color. Carotenes are a type of carotenoids that occur in various isomeric forms, among which β-carotenes, which are broken down and reduced to vitamin A in the human body [93]. Of the analyzed carotenoids (Supplementary Table S28), β-carotene was the only one that was detected and quantified in MR [42], with values ranging between 0.0954 µg/g and 0.3423 µg/g, which are in the same range as the ones described by Khoo et al. (2011) [93] for raspberries (0.08 µg/g) and blueberries (0.35 µg/g).

The total anthocyanin, ellagitannin, hydroxycinnamic acid, and hydroxybenzoic acid content, determined by HPLC, was assessed for MF (Table 12 and Supplementary Table S11). Regarding MF, for one of the articles, the total anthocyanins content is 385.09 ± 6.61 mg/100 g dry weight (DW) [50], whereas the anthocyanins content varies between 25.64 mg/g dried extract (DE) and 36.12 mg/g DE in the article by Spínola et. al. (2019) [54]. Table S11 shows that fruits from Arco de São Jorge (32°49′38″ N, 16°57′18″ O, Madeira Island, Portugal) have the highest anthocyanin content, whereas those from Ribeira da Janela (32°50′49″ N, 17°09′29″ O, Madeira Island, Portugal) have the lowest [54].

The content of the total hydroxybenzoic acids, with values ranging from 1.28 mg/g DE to 2.01 mg/g DE, is higher than MF’s total hydroxycinnamic acids (0.40 mg/g DE to 1.12 mg/g DE) and total ellagitannins content (0.77 mg/g DE to 1.35 mg/g DE) (Table 12 and Supplementary Table S11).

The contents of phytic acid, total oxalates, saponins, alkaloids and tannins, which are also known as anti-nutrients, are depicted in Table 13. These compounds are part of the plants’ defense mechanism; however, when consumed by humans, they can reduce the bioavailability of important nutrients [94]. The ME phytic acid content (3.67 ± 0.13 mg/100 g) is in the same range of values as the ones determined in the study by Onyenweaku et al. (2024) [95] for mango (4.51 ± 0.01 mg/100 g) and guava peels (3.85 ± 0.02 mg/100 g), whereas the oxalate content (6.60 ± 2.20 mg/100 g) is higher than reported in mango (0.46 ± 0.01 mg/100 g) and guava (2.87 ± 0.02 mg/100 g) peels. Regarding the tannins content, the values reported by Ngurthankhumi et al. (2024) [43] for ME (138.44 ± 1.02 mg/100 g) are considerably higher than the mango (0.01 ± 0.00 mg/100 g) and guava (1.92 ± 0.01 mg/100 g) peel values [95].

Concerning compound identification (Supplementary Table S29), a total of forty-one components were described, fifteen in MR [46,52] and twenty-seven in MF [50]. Of all the compounds, only cyanidin-3-*O*-glucoside was present in both MR and MF samples. The article by Spínola et al. (2014) [50] highlights compounds identified for the first time in the *Myrica* L. genus, namely, caffeic acid-*O*-hexoside, galloyl-*O*-hexoside, delphinidin-*O*-hexoside, luteolin-*O*-hexoside derivative, roseoside, (epi)catechin, gallo(epi)catechin-*O*-gallate, myricetin-*O*-hexoside, glucaric acid derivative, (epi)catechin-*O*-gallate, galloylquercetin-*O*- hexoside, benzoyl-p-dicoumaryl-2,7-anhydro-3-deoxy-2-octulopyranosonic acid, kaempferol-*O*-rhamnoside, tricin-*O*-hexoside, conidendrin-*O*-hexoside, 5,7-dihydroxy-6,8-dimethoxyflavone-7-*O*-glucuronide, benzoyl-p-tricoumaryl-2,7-anhydro-3-deoxy-2-octulopyranosonic acid and oxo-dihydroxy-octadecenoic acid.

In sum, with forty-one polyphenols identified and seventy-five quantified, the fruits of the *Myrica* L. genus have been shown to be rich in polyphenols, some of which have important health benefits and influence organoleptic characteristics. Furthermore, the species ME and MN exhibit a higher total phenolic and flavonoid content compared to other fruits such as blackberries, raspberries, goldenberries, cherries and strawberries [63,65,81]. Additionally, it was observed that maturation stage, location and variety influenced the chemical content.

### 3.5. Phytochemical, Physicochemical, and Nutritional Composition of the Leaves

#### 3.5.1. Physicochemical Parameters

Regarding the leaves, only one [51] of the sixteen articles collected information about physicochemical parameters. This study assessed the petiole length, leaf length and breath, color, moisture, total, acid-insoluble, water-soluble and sulphated ash. In addition, this study provides information on the yields of methanol, ethyl acetate and aqueous extracts, and on the foreign organic matter, foaming index and swelling index of ME leaves.

One of the studies, through an observational analysis, describes ME leaves as having a yellowish-green coloration when younger, becoming progressively darker when mature. Furthermore, the lower surface of the leaf presents a lighter shade of green in comparison to the upper surface [51].

The petiole length of ME leaves (8.72 cm) is considerably higher than that of the leaves of olive trees [96], which have a petiole length ranging from 0.31 to 0.65 cm. RThe length of ME leaves [51] varies between 5.00 cm and 10.20 cm; these values are in the same range as the ones for the olive tree (3.9 cm to 7.9 cm) and bay laurel (7.99 cm). The breadth of ME leaves [51] ranges from 2.00 cm to 3.50 cm, which is higher than the width of the leaves from olive trees (0.83 cm to 1.56 cm).

Moisture was one of the parameters evaluated, showing that the fresh ME leaf content (8.72%) [51] is significantly lower than the moisture content of fresh dandelion leaves (86.20%) [97]. The total, acid-insoluble, water-soluble and sulfated ash contents [51] are respectively, 2.83%, 0.52%, 0.38%, and 2.41%. The total ash content of *Sesbania grandiflora* leaves [98] varies between 7.50% and 7.75%, which is much higher than the ME content. The same is true for water-soluble ash, with *Sesbania grandiflora* [98] having higher values (3.50% to 3.65%) than ME. For both acid-insoluble ash and sulfated ash, the ME leaf values were lower than the ones found for papaya leaves [99], with 1.3% for acid-insoluble ash and 9.2% for sulfated ash.

Regarding the extractive value of ME leaves [51] using different solvents, the results show that methanol has a higher percentage (38.51%), followed by ethyl acetate (21.20%) and water (15.70%). These results differ from the ones presented in the study by Arawande et al. (2021) [100] for the moringa plant, in which the yield was 11.095 ± 0.805% in ethyl acetate, 8.497 ± 0.71% in water, and 8.108 ± 2.219% in methanol.

Foreign organic matter, the foaming index, and the swelling index were also evaluated for ME leaves. Whereas the foreign organic matter was less than 1%, the foaming and swelling indices were found to be nil [51]. These values differed from those of papaya leaves [99], in which foreign organic matter was nil and the foaming index was less than 100.

In summary, the only species of *Myrica* L. for which physicochemical parameters were evaluated was ME. These findings indicate that ME leaves have a low moisture and total ash content in comparison to other plant leaves and that the solvent with the highest percentage yield was methanol. Furthermore, regarding physical characteristics, the studies reported that the leaves’ colors range from a yellowish to a dark green, depending on maturity, and present a length similar to that of olive trees and bay laurel leaves.

#### 3.5.2. Nutritional Composition

The leaves’ nutritional composition was only assessed in one article and for one species of *Myrica* L. The results do not show the nutritional content but rather the presence or absence of carbohydrates, proteins, amino acids, fixed oils, and fats (Supplementary Table S30). In the case of ME leaves [51], carbohydrates were the only nutrient present in the sample, whereas proteins, amino acids, fixed oils, and fats were absent. In comparison, guava leaves have been shown to have all these macronutrients present [101], with the percentage of protein being the highest (18.53%), followed by carbohydrates (12.74%) and fats (0.62%).

In short, the evidence shows that ME leaves only have carbohydrates present; however, the nutritional composition of the leaves from various *Myrica* L. species should be further explored, evaluating the content of macro and micronutrients to determine if this part of the plant may have nutritional significance.

#### 3.5.3. Chemical Composition

Plant leaves are not only nutritious but also a source of bioactive and pharmacological compounds with important therapeutic properties [102,103].

The chemical composition of the leaves of three of the *Myrica* L. species, MR, ME and MF, was assessed in eight [45,47,50,51,53,54,55,56] of the sixteen articles, with the results for total phenolics, flavonoids, individual phenolics, phenolic acids, flavanols, flavonols, flavones, hydroxycinnamic acids, hydroxybenzoic acids and ellagitannins presented in Table 14, Table 15 and Table 16 and in the Supplementary Materials (Tables S31–S33). The content of specific polyphenols was also analyzed [45,50,54], as shown in Supplementary Tables S34–S45. In addition to a polyphenol content assessment, several articles [45,50,53,55,56] identified compounds in the leaves of MR, ME and MF (Supplementary Table S46). Furthermore, the presence or absence of anti-nutrients was also evaluated for ME [51].

The total phenolic content (Table 14 and Supplementary Table S31) has been evaluated for MR, ME and MF, with values ranging between 212.43 and 257.76 mg GAE/g DE for MF [50,54], while ME had values varying between 62.38 and 88.94 mg GAE/g [53] and MR had a content of 378.28 mg GAE/g DW [47]. The ME content is similar to the total phenolic content of *Adenanthera pavonina* leaves [104], with a value of 81.379 mg GAE/g. Table S31 shows that the leaves from Terceira (Azores Island, Portugal) have the highest total phenolic content, whereas those from Machico (Madeira Island, Portugal) have the lowest [54].

The results from Table 14 and Supplementary Table S31 indicate that the total flavonoid content was assessed for MR, ME and MF leaves. The MR leaf [45] content was 920.78 ± 18.88 mg RE/g DW and 669.18 ± 40.95 mg RE/g DW after in vitro digestion. These values are significantly higher than the ones presented for kenaf leaves [103], which have a total flavonoid content ranging from 1.55 to 9.24 mg RE/g DW. Regarding other *Myrica* L. species, the flavonoid content of ME leaves [53] ranges from 35.77 to 67.44 mg QE/g, and for MF [50,54] the values vary between 54.89 mg RE/g DE for leaves of Ribeira da Janela and 127.20 mg RE/g DE for leaves of Machico. In the article by Kabra et al. (2019) [51], the authors refer to the presence of phenolic and flavonoid compounds in ME leaves. Nevertheless, the total content is not assessed (Supplementary Table S31).

**Figure 11:**
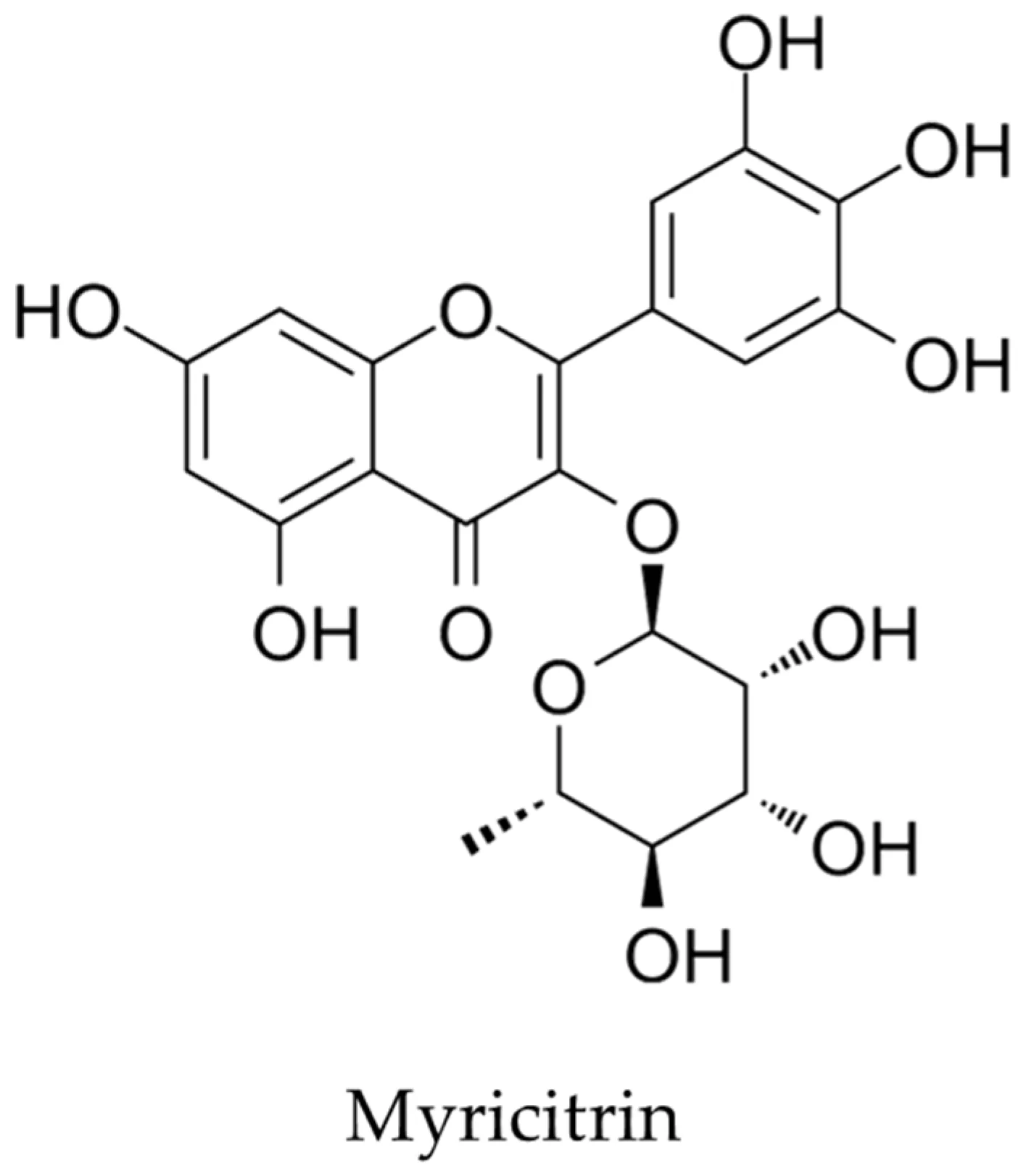
Myricitrin molecular structure.

The total individual phenolic, phenolic acid, flavanol, flavonol and flavone content is depicted in Table 15 and Supplementary Table S32. The results show that these parameters were only analyzed for MF leaves in two articles [50,54]. In the article by Spínola et al. (2014) [50], the total individual phenolic, phenolic acid, flavanol, flavonol and flavone contents are, respectively, 1540.38 ± 87.76 mg/100 g DW, 7.53 ± 0.9 mg/100 g DW, 178.04 ± 4.33 mg/100 g DW, 1241.97 ± 10.79 mg/100 g DW, and 26.38 ± 1.48 mg/100 g DW. The MF total phenolic acid leaf content [50] is lower than the value presented for *Urtica dioica* [105], at 1839.90 mg/100 g DW. In contrast, the total flavonol [50] content for MF leaves is considerably higher than in chokeberry leaves (284.5 mg/100 g DW in one sample and 288.1 mg/100 g DW in a second sample) [106]. Concerning total flavones, the leaves from *Annona cherimola* [107] have a similar content (28.70 ± 0.96 mg/100 g DW) to MF leaves [50], and both have a higher content than *Colocasia esculenta* (10.56 ± 0.15 mg/100 g DW) and *Smallanthus sonchifolius* (1.91 ± 0.01 mg/100 g DW) leaves [107]. The study by Spínola et al. (2019) [54] has shown that the total individual phenolic content ranges from 83.45 to 102.35 mg/g DE, the total flavanols content ranges from 21.99 to 30.78 mg/g DE, the total flavonols content ranges from 26.79 to 46.04 mg/g DE and the total flavone content ranges from 0.41 to 1.06 mg/g DE. A total of twenty-four individual flavonols (myricitrin, myricetin-*O*-hexoside, myricetin-*O*-(*O*-galloyl)hexoside, myricetin-*O*-deoxyhexoside, dimethyl-myricetin-*O*-pentoside, myricetin-*O*-(*O*-galloyl)deoxyhexoside, myricetin-*O*-(galloyl)hexoside, myricetin-*O*-(galloyl)deoxyhexoside, myricetin-*O*-pentoside, kaempferol-*O*-rutinoside, kaempferol-*O*-hexoside, kaempferol-*O*-(*O*-galloyl)hexoside, kaempferol-*O*-rhamnoside, kaempferol-*O*-(galloyl)hexoside, kaempferol-*O*-deoxyhexoside, quercetin 3-rhamnoside, quercetin-*O*-deoxyhexoside, quercetin-*O*-rutinoside, galloylquercetin-*O*-hexoside, quercetin-*O*-hexoside, quercetin-*O*-(galloyl)hexoside, quercetin-*O*-(galloyl)deoxyhexoside, quercetin-*O*-(acetyl)deoxyhexoside and quercetin) of MR [45] and MF [50,54] leaves were quantified (Supplementary Tables S36–S40) (Figure 11). In MF leaves, the quercetin-*O*-hexoside content (Supplementary Table S40) varies between 0.12 ± 0.01 mg/g DE and 0.70 ± 0.01 mg/g DE [54]. This content is considerably lower than the one present for *Macaranga hurifolia* and *Zanthoxylum gilletii* leaves, with 3.7 ± 0.2 mg/g DE and 1.47 ± 0.08 mg/g DE, respectively [108]. For quercetin-*O*-deoxyhexoside (Supplementary Table S39), MF leaf content is 16.86 ± 0.86 mg/100 g DW [50]. However, in the study conducted by Spínola et al. (2019) [54], it ranges between 0.56 ± 0.01 and 1.15 ± 0.01 mg/g DE. The latter values are higher than the ones for *Zanthoxylum gilletii* leaves (0.46 ± 0.03 mg/g DE) [108]. Kaempferol-*O*-hexoside was also one of the flavonoids whose content was assessed in MF leaves (Supplementary Table S38). In one of the two articles that conducted this analysis, the content was 16.65 ± 1.94 mg/100 g DW [50], and for the other article, the values ranged from 0.44 ± 0.01 to 0.86 ± 0.02 mg/g DE [54]. The leaves of *Macaranga hurifolia* [108] have a much higher content of this specific flavonoid (3.7 ± 0.2 mg/g DE) than MF leaves.

The content of individual flavanols is depicted in Supplementary Tables S41 and S42. In total, eight flavanols (galloyl-di(epi)-gallocatechin, (epi)catechin, gallo(epi)catechin-*O*-gallate dimer, gallocatechin, gallo(epi)catechin-gallo(epi)catechin-*O*-gallate, digalloyl(epi)gallocatechin dimer, gallo(epi)catechin-*O*-gallate and digallo(epi)catechin derivative) were quantified in MF leaves [50,54].

Regarding flavones in MF leaves [50,54], the contents of tricin-*O*-hexoside and tricin-*O*-hexoside derivative were analyzed and the results expressed in Supplementary Table S43.

Concerning hydroxycinnamic and hydroxybenzoic acids (Table 16 and Supplementary Table S33), the total hydroxycinnamic acid content in MF leaves varies between 0.03 mg/g DE and 0.24 mg/g DE, whereas the total hydroxybenzoic content ranges from 4.49 mg/g DE to 8.36 mg/g DE [54].

The content of two hydroxycinnamic acids present in MF leaves was determined, namely, coumaroyl-*O*-hexoside, with 0.21 ± 0.01 mg/g DE and benzoyl-p-tri-coumaroyl-2,7-anhydro-3-deoxy-2-octulopyranosonic acid, with values ranging from 0.03 ± 0.01 to 0.07 ± 0.01 mg/g DE [54] (Supplementary Table S34).

**Figure 12:**
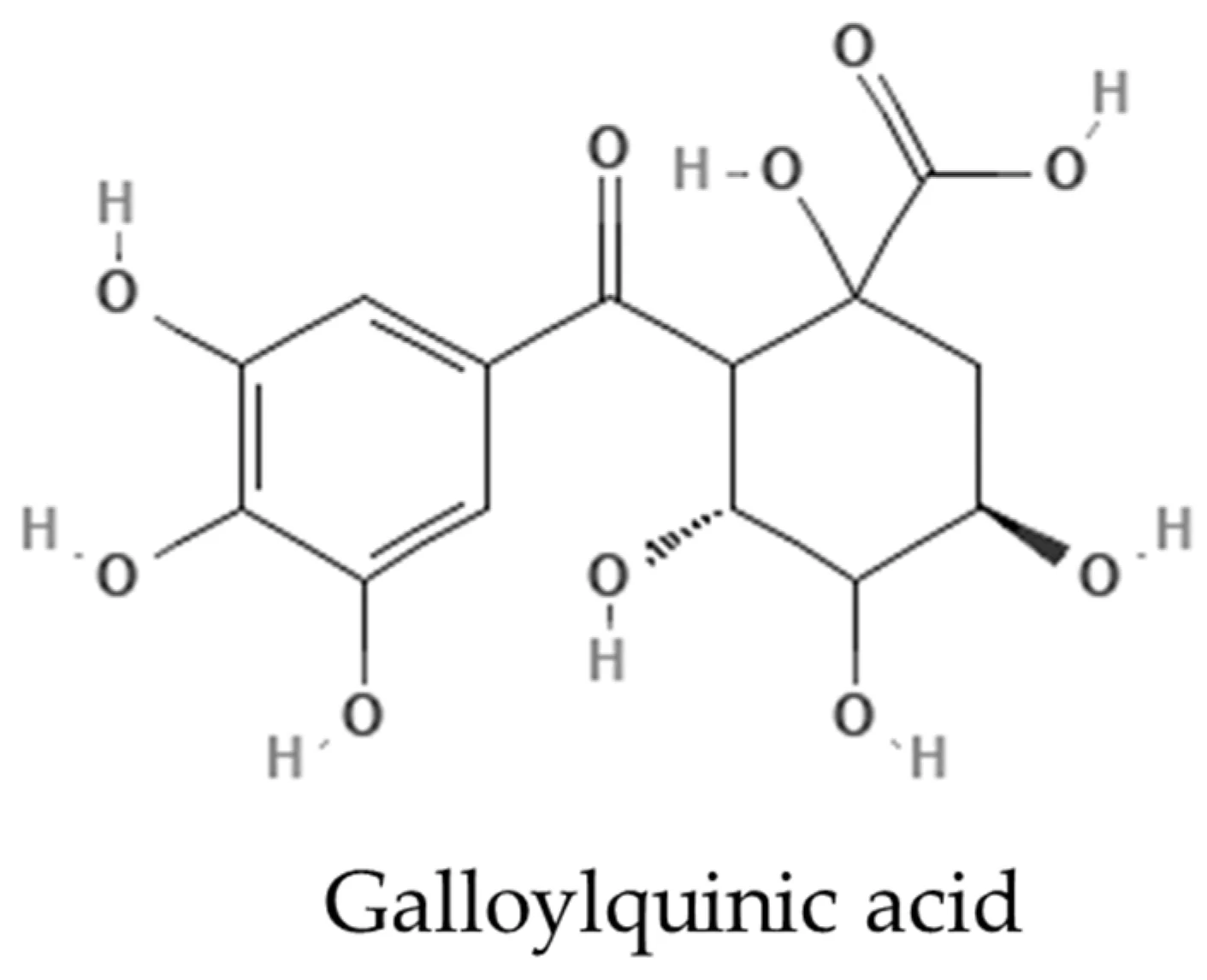
Galloylquinic acid molecular structure.

Pertaining to hydroxybenzoic acids, a total of six individual acids (galloyl-*O*-hexoside, galloylquinic acid, gallic acid, trigalloyl-*O*-hexoside, tetragalloyl-*O*-hexoside and protocatechuic acid-*O*-pentoside) were quantified in MF leaves [50,54] (Figure 12).

In relation to the total ellagitannins content (Table 16 and Supplementary Table S33), the results show that MF leaves present, in one of the articles [54], values between 18.96 and 33.79 mg/g DE, and in another article [50], 86.46 ± 6.75 mg/100 g DW. The strawberry leaves showed a total ellagitannins content of 8200 mg/100 g DW [109], which is significantly higher than for MF leaves. The content of individual ellagitannins, namely, galloyl-bis-hexahydroxydiphenoyl-*O*-hexoside, hexahydroxydiphenoyl-*O*-hexoside, casuarinin, ellagic acid-*O*-pentoside, pedunculagin I derivative, pedunculagin I, pedunculagin II, ellagic acid derivative and ellagitannin, is shown in Supplementary Tables S44 and S45.

In this review, one of the articles [51] analyses the presence of known anti-nutrient compounds, namely, alkaloids, glycosides, saponins, steroids, and tannins. The analysis revealed that glycosides and tannins are present in aqueous, ethyl acetate and methanolic extracts of ME leaves [51], whereas saponins and steroids are absent. For alkaloids, they are absent in aqueous extracts, but present in ethyl acetate and methanolic extracts. In *Adenanthera pavonina* leaves [104], some anti-nutrients, such as alkaloids, saponins, steroids and tannins, were detected in the conducted analysis. In comparison, the leaves have been shown to contain lower levels of anti-nutrients than the leaves of *Adenanthera pavonina*.

**Figure 13:**
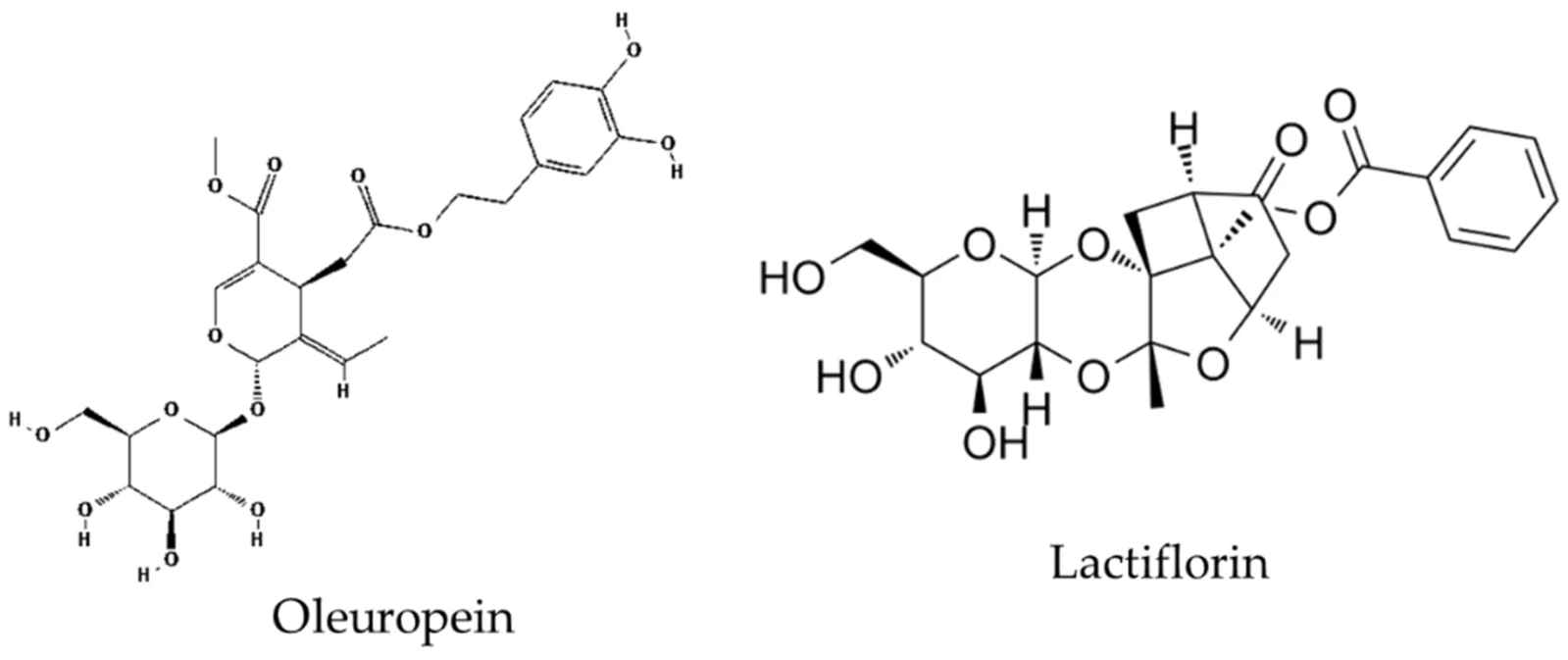
Oleuropein and lactiflorin molecular structures.

In addition to quantifying polyphenols, five [45,50,53,55,56] of the sixteen articles identify multiple components present in MR, ME, and MF leaves (Supplementary Table S46). A sum of two hundred and twenty-four compounds were described, one hundred and sixty-seven in MR, eighteen in ME and forty-two in MF. Of the identified components, gallic acid and myricanol were compounds that existed in both MR and ME leaves, and quinic acid was present in both MR and MF leaves. None of the compounds were present in all three analyzed species. One of the articles [50] showed several compounds that have been identified for the first time in the *Myrica* L. genus, namely, gallo(epi)catechin, benzyl alcohol hexose pentose, galloylquercetin-*O*-hexoside, kaempferol-*O*-rutinoside, kaempferol-*O*-hexoside, phylligenin-*O*-hexoside, kaempferol-*O*-(*O*-galloyl)hexoside, oleuropein (Figure 13), benzoyl-p-dicoumaryl-2,7-anhydro-3-deoxy-2-octulopyranosonic acid, benzoyl-p-tricoumaryl-2,7-anhydro-3-deoxy-2-octulopyranosonic acid, sinapic acid-*O*-hexoside derivative, ferulic acid-*O*-hexoside derivative, kaempferol-*O*-rhamnoside, dichotomitin-*O*-hexoside, lactiflorin (Figure 13), tricin-*O*-hexoside, baicalein derivative, conidendrin-*O*-hexoside and 5,7-dihydroxy-6,8-dimethoxyflavone-7-*O*-glucuronide.

Overall, a total of two hundred and twenty-four compounds were identified, and fifty-one were quantified in MR, ME and MF leaves. These components can potentially confer numerous bioactive properties to the leaves, highlighting prospects of utilizing the leaves from the species of the *Myrica* L. genus in pharmaceutic and food industries. The main results show that MR leaves have a higher total flavonoid content than kenaf leaves and MF has a higher total flavonol and flavone content in comparison to other species, such as chokeberry and *Colocasia esculenta* leaves. Additionally, it was observed that maturation stage, location and variety influenced the chemical content.

### 3.6. The Potential for Valorization of Fruits and Leaves from the Myrica L. Genus

Medicinal plants were traditionally used to treat various diseases using only empirical knowledge as a guide. Today, this ethnopharmacological knowledge forms the foundation for the discovery of hundreds of bioactive components incorporated into drugs to treat and prevent numerous diseases. The scientific evidence highlights that 25% to 28% of all medicines originated directly or indirectly from plants. Only about 6% of all plants have been pharmacologically analyzed and 15% phytochemically evaluated, which leaves many compounds with therapeutic potential to be discovered [110,111,112,113,114].

Plant-derived compounds have been recognized as high-valuable resources for the cosmetic, food, pharmaceutical, and nutraceutical industries, with either plant extracts, enriched fractions, or isolated compounds being incorporated into drugs, foods, dietary supplements, and cosmetics [111,114,115,116,117,118].

Plants are rich in phytonutrients and dietary bioactive compounds that are beneficial to human health. The fruits and leaves of the species from the *Myrica* L. genus are both composed of numerous phytochemicals with biological and pharmacological activity, namely, polyphenols. In comparison to many synthetic substances, polyphenols are biocompatible and naturally available products, making them a high-value nutra-pharmaceutical raw material. Furthermore, these compounds are known to prevent or treat chronic, degenerative and non-communicable diseases due to their antioxidant, antimicrobial, anti-inflammatory, anticancer and antimutagenic properties, as well as their protective effect in neuro, cardiovascular and liver health [19,27,113,119,120]. The review findings indicate that the fruits from ME and MF have a considerably higher content of total phenolics compared to their respective leaves. This suggests that the fruits may offer a greater potential health benefit.

Dietary polyphenols include, among others, two types of compounds: phenolic acids and flavonoids. The first can be classified into two categories, hydroxybenzoic and hydroxycinnamic acids, and evidence has shown numerous health benefits, namely, antioxidant, anti-inflammatory, anticancer, and antimicrobial activity [113,121,122]. This study revealed that MF fruits have a higher total phenolic acids content than leaves, with the same trend for the hydroxycinnamic acid content. For hydroxybenzoic acids, the opposite occurs, where the MF leaves have a greater content than the fruits.

The flavonoids class, similarly to phenolic acids, also presents various health benefits, such as anti-inflammatory, antitumor, and antioxidant properties, and consists of several subclasses, specifically, flavanols (flavan-3-ols), flavonols, isoflavones, flavones, flavanones, and anthocyanins [113,121,123]. The data from this research have shown that both fruits and leaves from species of the *Myrica* L. genus are rich in these components. The evidence indicates that MF leaves have a lower total flavonoid content than MF fruits; however, concerning total flavonols and flavanols, the leaves have a considerably higher content than the fruits. Anthocyanins are flavonoids consumed mainly in fruits and dark-colored vegetables, with important antioxidant activity, and thus offer multiple beneficial health effects [124,125]. As expected, this subclass of flavonoids has only been quantified for fruits of the *Myrica* L. genus, presenting similar values to other red fruits.

In terms of the analyzed individual flavonoids, some were found and quantified in both the fruits and the leaves, for example, myricitrin, and casuarinin, with the results showing that, concerning these compounds, leaves have a higher content than fruits for MF. Myricitrin has been reported to have health benefits, namely, antiviral, antitumor, antimicrobial and antinociception properties [126,127], while casuarinin has also been shown to have anticarcinogenic properties and potential antileishmanial activity [128,129].

Although phytochemical compounds are highly relevant, nutrients are also essential for humans, providing energy, acting in bone and tissue development and restoration, cell signaling, hormonal balance, and metabolic pathways, among other bodily functions [130]. The nutritional composition of the fruits and leaves was also assessed in this review for ME. Regarding nutrients, the leaves only presented carbohydrates, whereas the fruits contain all three macronutrients, with carbohydrates being the macronutrients present in the greatest quantity, followed by proteins and fats. The results have shown a high protein content in comparison to other fruits, such raspberries. The data show the presence of micronutrients, such as vitamins and minerals. Ascorbic acid is reported in MR, ME, and MN and vitamin E in ME. A total of seventeen minerals were identified in MF and eleven in ME, including calcium, copper, iron, magnesium, phosphorus, potassium, sodium and zinc.

The fruits and leaves of the species belonging to the *Myrica* L. genus have different nutritional and chemical compositions. Nevertheless, these species offer promising potential for industrial applications across several sectors. In the food industry, they may be used as a functional ingredient to incorporate into food products to enhance their nutritional and bioactive properties, and in the pharmaceutic and cosmetic industries, the extracts and phytonutrients obtained from these species may be formulated into pharmaceuticals, cosmetics or supplements. Both the fruits and leaves have shown a large variety of phytonutrients, which have or may potentially have pharmacological and industrial relevance.

## 4. Conclusions and Future Prospects

For millennia, plants have been used for a wide variety of purposes, from food to shelter, raw materials, and medicines, among others. Nowadays, due to the empirical knowledge that humans have about plants and the extensive research that has been conducted, it is widely recognized that plants are a valuable resource to humans, from plant-derived drugs, dyes, oils, and rubbers to other notable products. This study provides a systematic examination of the published scientific literature on the physical, phytochemical, and nutritional characteristics of fruits and leaves from the species of the *Myrica* L. genus.

This review summarizes the nutritional composition reported for one of the *Myrica* L. species, ME, showing a promising composition of multiple minerals and vitamins, which suggests that these fruits may be a good source of energy and nutrients, although further investigation should be conducted to clarify the nutritional significance.

Furthermore, several phytochemicals have been identified and quantified in both the leaves and the fruits. Some of these compounds have known health properties recognized and evidenced by the scientific literature, whereas others have yet to be phytochemically and pharmacologically investigated.

Although this systematic review has gathered relevant information surrounding the phytochemical, nutritional, and physical composition of different species from the *Myrica* L. genus, it still has some limitations. In particular, the use of exclusion criteria indicates that some scientific papers on the topic of this review were not included. The restriction to English-language, open-access publications and to articles from 2014 onwards may have resulted in the exclusion of relevant literature. Given the wide geographical distribution of the *Myrica* L. genus across Asia, as identified in this review, the restriction to English-language articles may have introduced a potential risk of bias. Consequently, the findings of this review reflect recent, English-language and open-access research and may not capture the entirety of the existing scientific knowledge on the *Myrica* L. genus. Moreover, the review included a limited number of studies, with considerable methodological heterogeneity. Furthermore, the included search terms were exclusively related to the *Myrica* L. genus, and historical nomenclatural changes and taxonomic reclassifications were not considered in the study, thus limiting the retrieval of manuscripts under alterative names. The total number of records retrieved was reported in the PRISMA flow chart; however, the number of records retrieved from each individual database was not recorded separately. In addition, the fact that the units used to describe the same parameters in the results section are different makes comparisons and subsequent interpretation difficult or even impossible. One other limitation concerns the evaluation of study quality, which has shown articles of moderate and low quality. Moreover, comparisons of nutritional and phytochemical characteristics are directly influenced by factors that may affect the reported values, including differences in cultivar, maturity stage, geographic origin, and analytical methodologies, which also limit comparability among studies. Additionally, the bibliometric revealed a skewed research focus toward MR and toward a specific geographical region, namely, China, which may indicate the presence of research bias.

The species of the *Myrica* L. genus show an array of chemical compounds, some with previously reported biological activities and uses in industry, others still to be explored. The findings from this study can contribute to the valorization of biologically relevant compounds, enhancing the untapped prospects of these species.

Future research should further investigate the phytochemical and nutritional compositions of the different species from the *Myrica* L. genus, as there is a lack of data on many other species in the genus. Additionally, future research should focus on identifying unknown compounds reported in these articles and exploring their potential use, applications and health benefits. Furthermore, more studies are needed on the bioavailability and metabolism of key compounds, as well as on agronomic practices, post-harvest handling and the effects of processing on chemical composition.

## Figures and Tables

**Table 1:** Inclusion and exclusion criteria.

**Inclusion Criteria**
English
Last 10 years (2014–2024)
Open-access
Article
Studies on the nutritional profile, physicochemical parameters and chemical composition of different species from the *Myrica* L. genus
Studies focusing on the fruits and/or leaves of different species from the *Myrica* L. genus
**Exclusion criteria**
Book chapters, conference papers, opinion articles, editorial material, review papers and meta-analysis
Off-topic
Chemical composition of essential oils
Clinical trials to analyze health effects
Chemical composition of by-products
Method optimization
Genomics
Studies that refer to the chemical composition in a previous works

**Table 2:** Physicochemical parameters of fruits from *Myrica* L. species.

Species of *Myrica* L.	Ref.	Moisture (%)	Weight (g)	Edible Rate (%)	Titratable Acidity (%)	pH
MR	(Chen et al. 2020; Zhang, 2015) [42,52]		8.81–28.17	94.30–97.39		2.63–2.92
ME	(Ngurthankhumi et al. 2024; Rymbai et al. 2023) [43,44]	86.75–89.00	13.26 ± 1.83 *		1.38–3.32	
MN	(Rymbai et al. 2023) [44]	83.62 ± 1.52 *	8.30 ± 0.56 *		2.45 ± 0.04 *	
MF	(Spínola et al. 2014) [50]					4.02

MR = *Myrica rubra*; ME = *Myrica esculenta*; MN = *Myrica nagi*; MF = *Myrica faya*. * Values expressed as mean ± standard deviation as reported in the original reference.

**Table 3:** Morphological and physical characteristics of fruits from *Myrica* L.

Species of *Myrica* L.	Ref.	Fruit Length (cm)	Fruit Diameter (cm)	Fruit Circumference (cm)	Fruit Volume (cm^3^)
ME	(Rymbai et al. 2023) [44]	3.14 ± 0.15 *	2.73 ± 0.11 *	8.13 ± 0.31 *	14.64 ± 1.76 *
MN	(Rymbai et al. 2023) [44]	1.52 ± 0.05 *	1.23 ± 0.08 *	3.64 ± 0.24 *	9.07 ± 0.70 *

ME = *Myrica esculenta*; MN = *Myrica nagi*. * Values expressed as mean ± standard deviation as reported in the original reference.

**Table 4:** Physicochemical parameters of seeds from *Myrica* L.

Species of *Myrica* L.	Ref.	Seed Weight (g)	Seed Length (mm)	Seed Breadth (mm)	Seed Number per Fruit
ME	(Rymbai et al. 2023) [44]	2.03 ± 0.06 *	19.62 ± 0.18 *	13.74 ± 0.03 *	1.00
MN	(Rymbai et al. 2023) [44]	1.32 ± 0.06 *	10.03 ± 0.05 *	0.82 ± 0.04 *	1.00

ME = *Myrica esculenta*; MN = *Myrica nagi*. * Values expressed as mean ± standard deviation as reported in the original reference.

**Table 5:** Color parameters (L*, a*, b* values, CIRG) of fruits from *Myrica* L. species.

Species of *Myrica* L.	Ref.	L* Value	a* Value	b* Value	CIRG
MR	(Zhang et al. 2015) [52]				1.96–12.39
ME	(Rymbai et al. 2023) [44]	54.2 ± 6.36 *	15.7 ± 4.29 *	29.6 ± 2.05 *	
MN	(Rymbai et al. 2023) [44]	25.6 ± 1.57 *	28.5 ± 1.51 *	9.3 ± 0.67 *	

MR = *Myrica rubra*; ME = *Myrica esculenta*; MN = *Myrica nagi*. * Values expressed as mean ± standard deviation as reported in the original reference.

**Table 6:** Nutritional composition of fruits from *Myrica* L. species.

Species of *Myrica* L.	Ref.	Energy (kcal)	Carbohydrates (%)	Starch (mg/100 g)	Protein (%)	Lipids (%)	Crude Fiber (%)
ME	(Ngurthankhumi et al. 2024) [43]	434.17 ± 13.36 *	79.84 ± 3.74 *	7.62 ± 0.02 *	25.02 ± 0.43 *	1.64 ± 0.03 *	4.72 ± 0.44 *

ME = *Myrica esculenta*. * Values expressed as mean ± standard deviation as reported in the original reference.

**Table 7:** Sugar content and total soluble solids (TSS) of fruits from *Myrica* L. species.

Species of *Myrica* L.	Ref.	Total Sugar (%)	Sucrose (mg/g FW)	Fructose (mg/g FW)	Glucose (mg/g FW)	TSS (ºBrix)
MR	(Chen et al. 2020; Zhang et al. 2015) [42,52]		40.00–65.85	5.00–14.66	5.00–12.33	8.89–11.67
ME	(Ngurthankhumi et al. 2024; Rymbai et al. 2023) [43,44]	4.27–17.59				5.83–12.03
MN	(Rymbai et al. 2023) [44]	6.83				6.76 ± 0.43 *
MF	(Spínola et al. 2014) [50]					14.87

MR = *Myrica rubra*; ME = *Myrica esculenta*; MN = *Myrica nagi*; MF = *Myrica faya*. * Values expressed as mean ± standard deviation as reported in the original reference.

**Table 8:** Organic acids content of fruits from *Myrica* L. species.

Species of *Myrica* L.	Ref.	Ascorbic Acid (mg/100 g)	Citric Acid (mg/g FW)	Malic Acid (mg/g FW)
MR	(Chen et al. 2020; Zhang et al. 2015) [42,52]	1.79–4.20	7.84–22.06	0.12–1.16
ME	(Ngurthankhumi et al. 2024; Rymbai et al. 2023) [43,44]	22.50–60.68		
MN	(Rymbai et al. 2023) [44]	7.50–15.00		

MR = *Myrica rubra*; ME = *Myrica esculenta*; MN = *Myrica nagi*.

**Table 9:** Total phenolic, flavonoid, and flavonol content determined by spectrophotometry.

Species of *Myrica* L.	Ref.	Total Phenolics	Total Flavonoids	Total Flavonols (mg QE/g)
MR	(Xia et al. 2023; Zhang et al. 2015) [52,57]	1.31–4.90 ^a^		
	(Zhang et al. 2015) [52]		0.85–1.91 ^c^	
	(Ren et al. 2019) [49]		20.00–60.00 ^d^	
ME	(Rymbai et al. 2023) [44]	10.00–15.00 ^a^	4.50–6.00 ^e^	1.00–2.00
MN	(Rymbai et al. 2023) [44]	10.00–15.00 ^a^	3.00–4.50 ^e^	1.00–2.00
MF	(Spínola et al. 2014) [50]	52.30 ^b,^*	6.68–42.10 ^f^	
	(Spínola et al. 2019) [54]	43.04–62.94 ^b^		

MR = *Myrica rubra*; ME = *Myrica esculenta*; MN = *Myrica nagi*; MF = *Myrica faya*. ^a^ mg GAE/g|^b^ mg GAE/g dried extract (DE)|^c^ mg RE/g fresh weight (FW)|^d^ mg/g dry weight (DW)|^e^ mg QE/g FW|^f^ mg RE/g DE. * Corrected TPC value (subtracted L-ascorbic acid L-AA contribution).

**Table 10:** Total individual phenolic, phenolic acid, flavonol, flavanol, and flavone content determined by HPLC.

Species of *Myrica* L.	Ref.	Total Individual Phenolics	Total Phenolic Acids (mg/100 g DW)	Total Flavonols	Total Flavanols	Total Flavones
MF	(Spínola et al. 2014) [50]	820.11 ± 46.71 ^a,^*	45.73 ± 0.97 *	268.81 ± 9.43 ^a,^*	37.80 ± 1.22 ^a,^*	82.69 ± 4.79 ^a,^*
	(Spínola et al. 2019) [54]	34.52–43.63 ^b^		2.93–6.26 ^b^	0.39–2.29 ^b^	0.11–0.25 ^b^

MF = *Myrica faya*. ^a^ mg/100 g dry weight (DW)|^b^ mg/g dried extract (DE). * Values expressed as mean ± standard deviation as reported in the original reference.

**Table 11:** Total anthocyanin and carotenoid content determined by spectrophotometry.

Species of *Myrica* L.	Ref.	Total Anthocyanins (mg/g)	Total Carotenoids (mg/g)
MR	(Chen et al. 2020; Yun et al. 2019) [42,46]	0.17–273.00	
	(Chen et al. 2020) [42]		5.00–12.00
ME	(Ngurthankhumi et al. 2024; Rymbai et al. 2023) [43,44]	0.70–3.10	0.01–0.12
MN	(Rymbai et al. 2023) [44]	1.50–1.70	0.13–0.15

MR = *Myrica rubra*; ME = *Myrica esculenta*; MN = *Myrica nagi*.

**Table 12:** Total anthocyanin, ellagitannin, hydroxycinnamic acid, and hydroxybenzoic acid content determined by HPLC.

Species of *Myrica* L.	Ref.	Total Anthocyanins	Total Ellagitannins (mg/g DE)	Total Hydroxycinnamic Acids (mg/g DE)	Total Hydroxybenzoic Acids (mg/g DE)
MF	(Spínola et al. 2014) [50]	385.09 ± 6.61 ^a,^*			
	(Spínola et al. 2019) [54]	25.64–36.13 ^b^	0.77–1.35	0.40–1.12	1.28–2.01

MF = *Myrica faya*. ^a^ mg/100 g dry weight (DW)|^b^ mg/g dried extract (DE). * Values expressed as mean ± standard deviation as reported in the original reference.

**Table 13:** Anti-nutrient content.

Species of *Myrica* L.	Ref.	Phytic Acid (mg/100 g)	Total Oxalate (mg/100 g)	Saponin (mg/100 g DGE)	Alkaloid (mg/100 g AE)	Tannin (mg/100 g)
ME	(Ngurthankhumi et al. 2024) [43]	3.67 ± 0.13 *	6.60 ± 2.20 *	6.94 ± 0.48 *	153.47 ± 3.53 *	138.44 ± 1.02 *

ME = *Myrica esculenta*. * Values expressed as mean ± standard deviation as reported in the original reference.

**Table 14:** Total phenolic and flavonoid content determined by spectrophotometry.

Species of *Myrica* L.	Ref.	Total Phenolics	Total Flavonoids
MR	(Zhang et al. 2016) [45]		920.78 ± 18.88 ^b,^*
	(Zhang et al. 2016) [45]		669.18 ± 40.95 ^b,^*^,§^
	(Zhang et al. 2018) [47]	378.28 ± 0.97 ^a,^*	
ME	(Kabra et al. 2019b) [53]	62.38–88.94 ^d^	35.77–67.44 ^c^
MF	(Spínola et al. 2019; Spínola et al. 2014) [50,54]	212.43–257.76 ^e^	54.89–127.20 ^f^

MR = *Myrica rubra*; ME = *Myrica esculenta*; MF = *Myrica faya*. ^a^ mg GAE/g DW|^b^ mg RE/g DW|^c^ mg/g QE|^d^ mg GAE/g|^e^ mg GAE/g DE|^f^ mg RE/g DE. * Values expressed as mean ± standard deviation as reported in the original reference. ^§^ in vitro digestion.

**Table 15:** Total individual phenolic, phenolic acid, flavanol, flavonol and flavone content determined by HPLC.

Species of *Myrica* L.	Ref.	Total Individual Phenolics	Total Phenolic Acids	Total Flavonols	Total Flavanols	Total Flavones
MF	(Spínola et al. 2014) [50]	1540.38 ± 87.76 ^a,^*	7.53 ± 0.9 ^a,^*	1241.97 ± 10.79 ^a,^*	178.04 ± 4.33 ^a,^*	26.38 ± 1.48 ^a,^*
	(Spínola et al. 2019) [54]	83.45–102.35 ^b^		26.79–46.04 ^b^	21.99–30.78 ^b^	0.41–1.06 ^b^

MF = *Myrica faya*. ^a^ mg/100 g DW|^b^ mg/g DE. * Values expressed as mean ± standard deviation as reported in the original reference.

**Table 16:** Total hydroxycinnamic acid, hydroxybenzoic acid and ellagitannin content determined by HPLC.

Species of *Myrica* L.	Ref.	Total Hydroxycinnamic Acids (mg/g DE)	Total Hydroxybenzoic Acids (mg/g DE)	Total Ellagitannins
MF	(Spínola et al. 2014) [50]			86.46 ± 6.75 ^a,^*
	(Spínola et al. 2019) [54]	0.03–0.24	4.49–8.36	18.96–33.79 ^b^

MF = *Myrica faya*. ^a^ mg/100 g DW|^b^ mg/g DE. * Values expressed as mean ± standard deviation as reported in the original reference.

## Data Availability

The original contributions presented in this study are included in the article/Supplementary Materials. Further inquiries can be directed to the corresponding author.

## Supplementary Materials

The following supporting information can be downloaded at: https://www.mdpi.com/article/10.3390/ijms27188237/s1.

## Abbreviations

The following abbreviations are used in this manuscript:

AE	Atropine equivalent
CIRG	Colour Index for Red Grapes
DE	Dried extract
DGE	Diosgenin equivalent
DW	Dry weight
FW	Fresh weight
GAE	Gallic acid equivalent
ME	*Myrica esculenta*
MF	*Myrica faya*
MN	*Myrica nagi*
MR	*Myrica rubra*
QE	Quercetin equivalent
RE	Rutin equivalent
TSS	Total soluble solids

## References

[1] Usman A.B., Abubakar S., Alaku C., Nnadi O. (2014). Plant: A Necessity of Life. Int. Lett. Nat. Sci..

[2] Knapp S. (2018). People and plants: The unbreakable bond. Plants People Planet.

[3] Schaal B. (2018). Plants and people: Our shared history and future. Plants People Planet.

[4] Moses T., Goossens A. (2017). Plants for human health: Greening biotechnology and synthetic biology. J. Exp. Bot..

[5] Raven P.H. (2020). Plants make our existence possible. Plants People Planet.

[6] Azizi A., Mahboob M., Monib A.W., Hassand M.H., Sediqi S., Niazi P. (2023). The Role of Plants in Human Health. Br. J. Biol. Stud..

[7] Ghorbanpour M., Hadian J., Nikabadi S., Varma A. (2017). Importance of Medicinal and Aromatic Plants in Human Life. Medicinal Plants and Environmental Challenges.

[8] (2024). The Editors of Encyclopaedia Britannica “*Myricaceae*”. *Encyclopedia Britannica*. https://www.britannica.com/plant/Myricaceae.

[9] Kubitzki K., Kubitzki K., Rohwer J.G., Bittrich V. (1993). Myricaceae. Flowering Plants · Dicotyledons: Magnoliid, Hamamelid and Caryophyllid Families.

[10] Sandall E.L., Maureaud A.A., Guralnick R., McGeoch M.A., Sica Y.V., Rogan M.S., Booher D.B., Edwards R., Franz N., Ingenloff K. (2023). A globally integrated structure of taxonomy to support biodiversity science and conservation. Trends Ecol. Evol..

[11] Bhatt I.D., Rawat S., Rawal R.S., Jaiswal A.K. (2020). Chapter 28—Himalayan bayberries. Nutritional Composition and Antioxidant Properties of Fruits and Vegetables.

[12] Board of Trustees of the Royal Botanic Gardens *Myrica* L.. https://powo.science.kew.org/taxon/urn:lsid:ipni.org:names:30023028-2.

[13] International Plant Names Index. http://www.ipni.org.

[14] Gtari M., Dawson J.O. (2011). An overview of actinorhizal plants in Africa. Funct. Plant Biol..

[15] Silva P.T.M., Silva M.A.F., Silva L., Seca A.M.L. (2019). Ethnobotanical Knowledge in Sete Cidades, Azores Archipelago: First Ethnomedicinal Report. Plants.

[16] Agrawal K., Vibha, Singh N. (2024). Pharmacognostical, phytochemical and pharmacological Aspects of *Myrica rubra* (Chinese Bayberry): An update. Pharmacol. Res. Mod. Chin. Med..

[17] Silva B.J.C., Seca A.M.L., Barreto M.D., Pinto D. (2015). Recent Breakthroughs in the Antioxidant and Anti-Inflammatory Effects of *Morella* and *Myrica* Species. Int. J. Mol. Sci..

[18] Ali G. (2011). Flavonoids and phenolic acids: Role and biochemical activity in plants and human. J. Med. Plants Res..

[19] Lin D.R., Xiao M.S., Zhao J.J., Li Z.H., Xing B.S., Li X.D., Kong M.Z., Li L.Y., Zhang Q., Liu Y.W. (2016). An Overview of Plant Phenolic Compounds and Their Importance in Human Nutrition and Management of Type 2 Diabetes. Molecules.

[20] Rana A., Samtiya M., Dhewa T., Mishra V., Aluko R.E. (2022). Health benefits of polyphenols: A concise review. J. Food Biochem..

[21] Ameer K., Shahbaz H.M., Kwon J.H. (2017). Green Extraction Methods for Polyphenols from Plant Matrices and Their Byproducts: A Review. Compr. Rev. Food Sci. Food Saf..

[22] Lourenço S.C., Moldao-Martins M., Alves V.D. (2019). Antioxidants of Natural Plant Origins: From Sources to Food Industry Applications. Molecules.

[23] Singh P., Konar A., Thakur M.K., Pandey K.B., Suttajit M. (2023). Chapter 11—An insight into plant polyphenols in prevention of brain aging. Plant Bioactives as Natural Panacea Against Age-Induced Diseases.

[24] Su X., Zhou D., Li N., Rahman A.-U. (2022). Chapter 8—Bioactive stilbenes from plants. Studies in Natural Products Chemistry.

[25] Altemimi A., Lakhssassi N., Baharlouei A., Watson D.G., Lightfoot D.A. (2017). Phytochemicals: Extraction, Isolation, and Identification of Bioactive Compounds from Plant Extracts. Plants.

[26] Dias M.C., Pinto D., Silva A.M.S. (2021). Plant Flavonoids: Chemical Characteristics and Biological Activity. Molecules.

[27] Samtiya M., Aluko R.E., Dhewa T., Moreno-Rojas J.M. (2021). Potential Health Benefits of Plant Food-Derived Bioactive Components: An Overview. Foods.

[28] Rosa G.P., Silva B.J.C., Seca A.M.L., Moujir L.M., Barreto M.C. (2020). Phytochemicals with Added Value from *Morella* and *Myrica* Species. Molecules.

[29] Ahmad G., Khan S.U., Mir S.A., Iqbal M.J., Pottoo F.H., Dhiman N., Malik F., Ali A. (2022). *Myrica esculenta Buch.-Ham. (ex D. Don)*: A Review on its Phytochemistry, Pharmacology and Nutritional Potential. Comb. Chem. High Throughput Screen.

[30] Bhatia D., Goyal P. (2021). A review of pharmacological and pharmacognostic profile of *Myrica nagi*. Res. J. Pharm. Technol..

[31] Bhatt S.C., Kumar V., Gupta A.K., Mishra S., Naik B., Rustagi S., Preet M.S. (2023). Insights on bio-functional properties of *Myrica esculenta* plant for nutritional and livelihood security. Food Chem. Adv..

[32] He X.H., Chen L.G., Asghar S., Chen Y. (2004). Red Bayberry (*Myrica rubra*), a promising fruit and forest tree in China. J. Am. Pomol. Soc..

[33] Kabra A., Martins N., Sharma R., Kabra R., Baghel U.S. (2019). *Myrica esculenta* Buch.-Ham. ex D. Don: A Natural Source for Health Promotion and Disease Prevention. Plants.

[34] Mo J.L., Rashwan A.K., Osman A.I., Eletmany M.R., Chen W. (2024). Potential of Chinese Bayberry (*Myrica rubra* Sieb. et Zucc.) Fruit, Kernel, and Pomace as Promising Functional Ingredients for the Development of Food Products: A Comprehensive Review. Food Bioprocess Technol..

[35] Skene K.R., Sprent J.I., Raven J.A., Herdman L. (2000). *Myrica gale* L.. J. Ecol..

[36] Sood P., Shri R. (2018). A Review on Ethnomedicinal, Phytochemical and Pharmacological Aspects of *Myrica esculenta*. Indian J. Pharm. Sci..

[37] Sun C.D., Huang H.Z., Xu C.J., Li X., Chen K.S. (2013). Biological Activities of Extracts from Chinese Bayberry (*Myrica rubra* Sieb. et Zucc.): A Review. Plant Foods Hum. Nutr..

[38] Zhang S., Yu Z., Sun L., Ren H., Zheng X., Liang S., Qi X. (2022). An overview of the nutritional value, health properties, and future challenges of Chinese bayberry. PeerJ.

[39] Moher D., Liberati A., Tetzlaff J., Altman D.G., Group P. (2010). Preferred reporting items for systematic reviews and meta-analyses: The PRISMA statement. Int. J. Surg..

[40] Knobloch K., Yoon U., Vogt P.M. (2011). Preferred reporting items for systematic reviews and meta-analyses (PRISMA) statement and publication bias. J. Craniomaxillofac. Surg..

[41] Page M.J., McKenzie J.E., Bossuyt P.M., Boutron I., Hoffmann T.C., Mulrow C.D., Shamseer L., Tetzlaff J.M., Akl E.A., Brennan S.E. (2021). The PRISMA 2020 statement: An updated guideline for reporting systematic reviews. Res. Methods Rep..

[42] Chen F., Wang Y., Ni H., Yan B. (2020). The 3at gene determines fruit color of *Myrica rubra* (*Myricaceae*). Gen. Mol. Res..

[43] Ngurthankhumi R., Hazarika T.K., Lalruatsangi E., Lalnunsangi T., Lalhmangaihzuali H.P. (2024). Bioactive constituents and health promoting compounds of few wild edible fruits of North-East India. Int. J. Food Prop..

[44] Rymbai H., Verma V.K., Talang H., Assumi S.R., Devi M.B., Sangma R.H.C.H., Biam K.P., Chanu L.J., Makdoh B., Singh A.R. (2023). Biochemical and antioxidant activity of wild edible fruits of the eastern Himalaya, India. Front. Nutr..

[45] Zhang Y., Chen S., Wei C., Gong H., Li L., Ye X. (2016). Chemical and Cellular Assays Combined with In Vitro Digestion to Determine the Antioxidant Activity of Flavonoids from Chinese Bayberry (*Myrica rubra* Sieb. et Zucc.) Leaves. PLoS ONE.

[46] Yun D.W., Cai H.H., Liu Y.P., Xiao L.X., Song J.F., Liu J. (2019). Development of active and intelligent films based on cassava starch and Chinese bayberry (*Myrica rubra* Sieb. et Zucc.) anthocyanins. RSC Adv..

[47] Zhang Y., Chen S., Wei C., Rankin G.O., Rojanasakul Y., Ren N., Ye X., Chen Y.C. (2018). Dietary Compound Proanthocyanidins from Chinese bayberry (*Myrica rubra* Sieb. et Zucc.) leaves inhibit angiogenesis and regulate cell cycle of cisplatin-resistant ovarian cancer cells via targeting Akt pathway. J. Funct. Foods.

[48] Llorent-Martinez E.J., Spinola V., Castilho P.C. (2017). Evaluation of the inorganic content of six underused wild berries from Portugal: Potential new sources of essential minerals. J. Food Compost. Anal..

[49] Ren H., Yu H., Zhang S., Liang S., Zheng X., Zhang S., Yao P., Zheng H., Qi X. (2019). Genome sequencing provides insights into the evolution and antioxidant activity of Chinese bayberry. BMC Genom..

[50] Spínola V., Llorent-Martínez E.J., Gouveia S., Castilho P.C. (2014). *Myrica faya*: A new source of antioxidant phytochemicals. J. Agric. Food Chem..

[51] Kabra A., Sharma R., Singla S., Kabra R., Baghel U.S. (2019). Pharmacognostic characterization of *Myrica esculenta* leaves. J. Ayurveda Integr. Med..

[52] Zhang X., Huang H., Zhang Q., Fan F., Xu C., Sun C., Li X., Chen K. (2015). Phytochemical Characterization of Chinese Bayberry (*Myrica rubra* Sieb. et Zucc.) of 17 Cultivars and Their Antioxidant Properties. Int. J. Mol. Sci..

[53] Kabra A., Sharma R., Hano C., Kabra R., Martins N., Baghel U.S. (2019). Phytochemical Composition, Antioxidant, and Antimicrobial Attributes of Different Solvent Extracts from *Myrica esculenta* Buch.-Ham. ex. D. Don Leaves. Biomolecules.

[54] Spínola V., Llorent-Martínez E.J., Castilho P.C. (2019). Polyphenols of *Myrica faya* inhibit key enzymes linked to type II diabetes and obesity and formation of advanced glycation end-products (in vitro): Potential role in the prevention of diabetic complications. Food Res. Int..

[55] Fu Y., Qiao L., Cao Y., Zhou X., Liu Y., Ye X. (2014). Structural elucidation and antioxidant activities of proanthocyanidins from Chinese bayberry (*Myrica rubra* Sieb. et Zucc.) leaves. PLoS ONE.

[56] Chen P., Lin X., Yang C.H., Tang X., Chang Y.W., Zheng W., Luo L., Xu C., Chen Y.H. (2017). Study on Chemical Profile and Neuroprotective Activity of *Myrica rubra* Leaf Extract. Molecules.

[57] Xia W., Gong E.R., Lin Y.Y., Zheng B.S., Yang W.H., Li T., Zhang S., Li P., Liu R.H. (2023). Wild pink bayberry free phenolic extract induces mitochondria-dependent apoptosis and G0/G1 cell cycle arrest through p38/MAPK and PI3K/Akt pathway in MDA-MB-231 cancer cells. Food Sci. Hum. Wellness.

[58] Joanna Briggs Institute (2014). MAStARI: Joanna Briggs Institute Reviewer’s Manual 2014.

[59] Gadioli I.L., da Cunha M.S.B., de Carvalho M.V.O., Costa A.M., Pineli L.L.O. (2018). A systematic review on phenolic compounds in Passiflora plants: Exploring biodiversity for food, nutrition, and popular medicine. Crit. Rev. Food Sci. Nutr..

[60] McGuinness L.A., Higgins J.P.T. (2021). Risk-of-bias VISualization (robvis): An R package and Shiny web app for visualizing risk-of-bias assessments. Res. Synth. Methods.

[61] Gonçalves A.C., Campos G., Alves G., Garcia-Viguera C., Moreno D.A., Silva L.R. (2021). Physical and phytochemical composition of 23 Portuguese sweet cherries as conditioned by variety (or genotype). Food Chem..

[62] Vursavuş K., Kelebek H., Selli S. (2006). A study on some chemical and physico-mechanic properties of three sweet cherry varieties (*Prunus avium* L.) in Turkey. J. Food Eng..

[63] Ruiz-Torralba A., Guerra-Hernández E., Garcia-Villanova B. (2018). Antioxidant capacity, polyphenol content and contribution to dietary intake of 52 fruits sold in Spain. CyTA-J. Food.

[64] Tosun M., Ercisli S., Karlidag H., Sengul M. (2009). Characterization of Red Raspberry (*Rubus idaeus* L.) Genotypes for Their Physicochemical Properties. J. Food Sci..

[65] Yıldız G., İzli N., Ünal H., Uylaşer V. (2015). Physical and chemical characteristics of goldenberry fruit (*Physalis peruviana* L.). J. Food Sci. Technol..

[66] Ilhan G., Gundogdu M., Karlović K., Židovec V., Vokurka A., Ercişli S. (2021). Main Agro-Morphological and Biochemical Berry Characteristics of Wild-Grown Sea Buckthorn (*Hippophae rhamnoides* L. ssp. caucasica Rousi) Genotypes in Turkey. Sustainability.

[67] Tang X., Tigerstedt P.M.A. (2001). Variation of physical and chemical characters within an elite sea buckthorn (*Hippophae rhamnoides* L.) breeding population. Sci. Hortic..

[68] Scalisi A., O’Connell M.G., Islam M.S., Goodwin I. (2022). A Fruit Colour Development Index (CDI) to Support Harvest Time Decisions in Peach and Nectarine Orchards. Horticulturae.

[69] Sharma S., Katoch V., Kumar S., Chatterjee S. (2021). Functional relationship of vegetable colors and bioactive compounds: Implications in human health. J. Nutr. Biochem..

[70] Carreño J., Martínez A., Almela L., Fernández-López J.A. (1995). Proposal of an index for the objective evaluation of the colour of red table grapes. Food Res. Int..

[71] Fernández-López J., Almela L., Muñoz J.A., Hidalgo V., Carreño J. (1998). Dependence between colour and individual anthocyanin content in ripening grapes. Food Res. Int..

[72] Ly B., Dyer E., Feig J., Chien A., Bino S. (2020). Research Techniques Made Simple: Cutaneous Colorimetry: A Reliable Technique for Objective Skin Color Measurement. J. Investig. Dermatol..

[73] Guiné R., Almeida C., Correia P., Mendes M. (2015). Modelling the Influence of Origin, Packing and Storage on Water Activity, Colour and Texture of Almonds, Hazelnuts and Walnuts Using Artificial Neural Networks. Food Bioprocess Technol..

[74] Di Vittori L., Mazzoni L., Battino M., Mezzetti B. (2018). Pre-harvest factors influencing the quality of berries. Sci. Hortic..

[75] Tyagi S., Sahay S., Imran M., Rashmi K., Mahesh S. (2017). Pre-harvest factors influencing the postharvest quality of fruits: A review. Curr. J. Appl. Sci. Technol..

[76] Tiwari U., Cummins E. (2013). Factors influencing levels of phytochemicals in selected fruit and vegetables during pre- and post-harvest food processing operations. Food Res. Int..

[77] Schulz M., Chim J.F. (2019). Nutritional and bioactive value of *Rubus* berries. Food Biosci..

[78] Basak J.K., Madhavi B.G., Paudel B., Kim N.E., Kim H.T. (2022). Prediction of Total Soluble Solids and pH of Strawberry Fruits Using RGB, HSV and HSL Colour Spaces and Machine Learning Models. Foods.

[79] Zhang J., Nie J.-y., Li J., Zhang H., Li Y., Farooq S., Bacha S.A.S., Wang J. (2020). Evaluation of sugar and organic acid composition and their levels in highbush blueberries from two regions of China. J. Integr. Agric..

[80] Zhao Y. (2007). Berry Fruit: Value-Added Products for Health Promotion.

[81] Subbiah V., Zhong B., Nawaz M.A., Barrow C.J., Dunshea F.R., Suleria H.A.R. (2020). Screening of Phenolic Compounds in Australian Grown Berries by LC-ESI-QTOF-MS/MS and Determination of Their Antioxidant Potential. Antioxidants.

[82] Orsavová J., Juríková T., Bednaříková R., Mlček J. (2023). Total Phenolic and Total Flavonoid Content, Individual Phenolic Compounds and Antioxidant Activity in Sweet Rowanberry Cultivars. Antioxidants.

[83] Imran M., Saeed F., Hussain G., Imran A., Mehmood Z., Gondal T.A., El-Ghorab A., Ahmad I., Pezzani R., Arshad M.U. (2021). Myricetin: A comprehensive review on its biological potentials. Food Sci. Nutr..

[84] Semwal D.K., Semwal R.B., Combrinck S., Viljoen A. (2016). Myricetin: A Dietary Molecule with Diverse Biological Activities. Nutrients.

[85] Dabeek W.M., Marra M.V. (2019). Dietary Quercetin and Kaempferol: Bioavailability and Potential Cardiovascular-Related Bioactivity in Humans. Nutrients.

[86] Guimarães R., Barros L., Dueñas M., Carvalho A.M., Queiroz M.J.R.P., Santos-Buelga C., Ferreira I.C.F.R. (2013). Characterisation of phenolic compounds in wild fruits from Northeastern Portugal. Food Chem..

[87] Salau V.F., Erukainure O.L., Islam M.S., Preedy V.R. (2020). Chapter 29—Phenolics: Therapeutic applications against oxidative injury in obesity and type 2 diabetes pathology. Pathology.

[88] Jimenez-Garcia S.N., Guevara-Gonzalez R.G., Miranda-Lopez R., Feregrino-Perez A.A., Torres-Pacheco I., Vazquez-Cruz M.A. (2013). Functional properties and quality characteristics of bioactive compounds in berries: Biochemistry, biotechnology, and genomics. Food Res. Int..

[89] Olivas-Aguirre F.J., Rodrigo-García J., Martínez-Ruiz N.D.R., Cárdenas-Robles A.I., Mendoza-Díaz S.O., Álvarez-Parrilla E., González-Aguilar G.A., De la Rosa L.A., Ramos-Jiménez A., Wall-Medrano A. (2016). Cyanidin-3-O-glucoside: Physical-Chemistry, Foodomics and Health Effects. Molecules.

[90] Husain A., Chanana H., Khan S.A., Dhanalekshmi U.M., Ali M., Alghamdi A.A., Ahmad A. (2022). Chemistry and Pharmacological Actions of Delphinidin, a Dietary Purple Pigment in Anthocyanidin and Anthocyanin Forms. Front. Nutr..

[91] Chai Z., Herrera-Balandrano D.D., Yu H., Beta T., Zeng Q., Zhang X., Tian L., Niu L., Huang W. (2021). A comparative analysis on the anthocyanin composition of 74 blueberry cultivars from China. J. Food Compost. Anal..

[92] Toledo-Martín E.M., García-García M.D.C., Font R., Moreno-Rojas J.M., Salinas-Navarro M., Gómez P., Del Río-Celestino M. (2018). Quantification of Total Phenolic and Carotenoid Content in Blackberries (*Rubus fructicosus* L.) Using Near Infrared Spectroscopy (NIRS) and Multivariate Analysis. Molecules.

[93] Khoo H.-E., Prasad K., Kong K.-W., Jiang Y., Ismail A. (2011). Carotenoids and Their Isomers: Color Pigments in Fruits and Vegetables. Molecules.

[94] López-Moreno M., Garcés-Rimón M., Miguel M. (2022). Antinutrients: Lectins, goitrogens, phytates and oxalates, friends or foe?. J. Funct. Foods.

[95] Onyenweaku E.O., Kesa H. (2024). Micronutrient and antinutrient content of semi-processed fruit peels: Towards boosting immunity. Health SA Gesondheid.

[96] Koubouris G., Bouranis D., Vogiatzis E., Nejad A.R., Giday H., Tsaniklidis G., Ligoxigakis E.K., Blazakis K., Kalaitzis P., Fanourakis D. (2018). Leaf area estimation by considering leaf dimensions in olive tree. Sci. Hortic..

[97] Laryea D., Barimah J., Yanney P., Quarcoo C. (2017). Effect of Drying Methods on Phytochemicals, Antioxidant Activity and Total Phenolic Content of Dandelion Leaves. Am. J. Food Nutr..

[98] Momin R.K., Kadam V.B. (2011). Determination of Ash Values of Some Medicinal Plants of Genus Sesbania of Marathwada Region in Maharashtra. J. Phytol..

[99] Mahadevi M., Azhagu Madhavan S. (2021). Natural movement and therapeutic different bioactive constituents and biochemical composition of (*Carica papaya* l.). Int. J. Bot. Stud..

[100] Arawande J., Adeleke A., Orimoloye O., Adebisi S., Amuho E., Olugbenga Olufemi I. (2021). Extractive Values and Antioxidant Properties of Leaves, Seeds, Pods and Coats of Moringa Plant. Biomed. J. Sci. Tech. Res..

[101] Shabbir H., Kausar T., Noreen S., Rehman H.u., Hussain A., Huang Q., Gani A., Su S., Nawaz A. (2020). In Vivo Screening and Antidiabetic Potential of Polyphenol Extracts from Guava Pulp, Seeds and Leaves. Animals.

[102] Sabbatini G., Mari E., Ortore M.G., Di Gregorio A., Fattorini D., Di Carlo M., Galeazzi R., Vignaroli C., Simoni S., Giorgini G. (2024). Hop leaves: From waste to a valuable source of bioactive compounds—A multidisciplinary approach to investigating potential applications. Heliyon.

[103] Birhanie Z.M., Xiao A., Yang D., Huang S., Zhang C., Zhao L., Liu L., Li J., Chen A., Tang H. (2021). Polysaccharides, Total Phenolic, and Flavonoid Content from Different Kenaf (*Hibiscus cannabinus* L.) Genotypes and Their Antioxidants and Antibacterial Properties. Plants.

[104] Agustin Y., Endang H., Anis A., Feni D., Kadek Niti P., Sya’idah Nur F. (2023). Total Phenolic Content and Antioxidant Activities of Leaves and Bark Extract of *Adenanthera pavonina* L.. Nat. Prod. Sci..

[105] Styczyńska S., Przybył J., Kosakowska O., Węglarz Z., Baczek K. (2023). Intraspecific Variability of Stinging Nettle (*Urtica dioica* L.). Molecules.

[106] Szopa A., Kokotkiewicz A., Kubica P., Banaszczak P., Wojtanowska-Krośniak A., Krosniak M., Marzec-Wróblewska U., Badura A., Zagrodzki P., Bucinski A. (2017). Comparative analysis of different groups of phenolic compounds in fruit and leaf extracts of Aronia sp.: A. melanocarpa, *A. arbutifolia*, and *A. ×prunifolia* and their antioxidant activities. Eur. Food Res. Technol..

[107] Barros J.G.L., Fernandes R., Abraão A., Costa R.D., Aires A., Gouvinhas I., Granato D., Barros A.N. (2024). Characterization of Azorean Plant Leaves for Sustainable Valorization and Future Advanced Applications in the Food, Cosmetic, and Pharmaceutical Industries. Antioxidants.

[108] Sadeer N.B., Llorent-Martínez E.J., Bene K., Fawzi Mahomoodally M., Mollica A., Ibrahime Sinan K., Stefanucci A., Ruiz-Riaguas A., Fernández-de Córdova M.L., Zengin G. (2019). Chemical profiling, antioxidant, enzyme inhibitory and molecular modelling studies on the leaves and stem bark extracts of three African medicinal plants. J. Pharm. Biomed. Anal..

[109] Karlińska E., Masny A., Cieślak M., Macierzyński J., Pecio Ł., Stochmal A., Kosmala M. (2021). Ellagitannins in roots, leaves, and fruits of strawberry (*Fragaria × ananassa* Duch.) vary with developmental stage and cultivar. Sci. Hortic..

[110] Atanasov A.G., Waltenberger B., Pferschy-Wenzig E.M., Linder T., Wawrosch C., Uhrin P., Temml V., Wang L.M., Schwaiger S., Heiss E.H. (2015). Discovery and resupply of pharmacologically active plant-derived natural products: A review. Biotechnol. Adv..

[111] Batiha G.E., Beshbishy A.M., Ikram M., Mulla Z.S., Abd El-Hack M.E., Taha A.E., Algammal A.M., Elewa Y.H.A. (2020). The Pharmacological Activity, Biochemical Properties, and Pharmacokinetics of the Major Natural Polyphenolic Flavonoid: Quercetin. Foods.

[112] Chopra B., Dhingra A.K. (2021). Natural products: A lead for drug discovery and development. Phytother. Res..

[113] Fraga C.G., Croft K.D., Kennedy D.O., Tomás-Barberán F.A. (2019). The effects of polyphenols and other bioactives on human health. Food Funct..

[114] Fridlender M., Kapulnik Y., Koltai H. (2015). Plant derived substances with anti-cancer activity: From folklore to practice. Front. Plant Sci..

[115] Kumar M., Tomar M., Amarowicz R., Saurabh V., Nair M.S., Maheshwari C., Sasi M., Prajapati U., Hasan M., Singh S. (2021). Guava (*Psidium guajava* L.) Leaves: Nutritional Composition, Phytochemical Profile, and Health-Promoting Bioactivities. Foods.

[116] Ma X.Y., Yang W., Kallio H., Yang B.R. (2022). Health promoting properties and sensory characteristics of phytochemicals in berries and leaves of sea buckthorn (*Hippophae rhamnoides*). Crit. Rev. Food Sci. Nutr..

[117] Thomford N.E., Senthebane D.A., Rowe A., Munro D., Seele P., Maroyi A., Dzobo K. (2018). Natural Products for Drug Discovery in the 21st Century: Innovations for Novel Drug Discovery. Int. J. Mol. Sci..

[118] Zhang Z.H., Li X.J., Sang S.Y., McClements D.J., Chen L., Long J., Jiao A.Q., Jin Z.Y., Qiu C. (2022). Polyphenols as Plant-Based Nutraceuticals: Health Effects, Encapsulation, Nano-Delivery, and Application. Foods.

[119] Joseph S.V., Edirisinghe I., Burton-Freeman B.M. (2016). Fruit Polyphenols: A Review of Anti-inflammatory Effects in Humans. Crit. Rev. Food Sci. Nutr..

[120] Talhaoui N., Taamalli A., Gómez-Caravaca A.M., Fernández-Gutiérrez A., Segura-Carretero A. (2015). Phenolic compounds in olive leaves: Analytical determination, biotic and abiotic influence, and health benefits. Food Res. Int..

[121] Li W., Chen H., Xu B., Wang Y., Zhang C., Cao Y., Xing X. (2023). Research progress on classification, sources and functions of dietary polyphenols for prevention and treatment of chronic diseases. J. Future Foods.

[122] Fernández de Córdova M.L., Medina A.R., Preedy V. (2014). Chapter 29—Analytical Methods for Determination of Polyphenols in Beer. Processing and Impact on Antioxidants in Beverages.

[123] Papoutsis K., Zhang J., Bowyer M.C., Brunton N., Gibney E.R., Lyng J. (2021). Fruit, vegetables, and mushrooms for the preparation of extracts with α-amylase and α-glucosidase inhibition properties: A review. Food Chem..

[124] Krga I., Milenkovic D. (2019). Anthocyanins: From Sources and Bioavailability to Cardiovascular-Health Benefits and Molecular Mechanisms of Action. J. Agric. Food Chem..

[125] Nile S.H., Park S.W. (2014). Edible berries: Bioactive components and their effect on human health. Nutrition.

[126] Qi S., Feng Z., Li Q., Qi Z., Zhang Y. (2017). Myricitrin Modulates NADPH Oxidase-Dependent ROS Production to Inhibit Endotoxin-Mediated Inflammation by Blocking the JAK/STAT1 and NOX2/p47phox Pathways. Oxid. Med. Cell. Longev..

[127] Domitrović R., Rashed K., Cvijanović O., Vladimir-Knežević S., Škoda M., Višnić A. (2015). Myricitrin exhibits antioxidant, anti-inflammatory and antifibrotic activity in carbon tetrachloride-intoxicated mice. Chem.-Biol. Interact..

[128] Rajasekar N., Sivanantham A., Ravikumar V., Rajasekaran S. (2021). An overview on the role of plant-derived tannins for the treatment of lung cancer. Phytochemistry.

[129] Tiwari N., Kumar A., Singh A.K., Bajpai S., Agrahari A.K., Kishore D., Tiwari V.K., Singh R.K., Brahmachari G. (2019). Leishmaniasis control: Limitations of current drugs and prospects of natural products. Discovery and Development of Therapeutics from Natural Products Against Neglected Tropical Diseases.

[130] Gush L., Shah S., Gilani F., Short E. (2021). Chapter 23—Macronutrients and micronutrients. A Prescription for Healthy Living.

