# Supplementary material for: Phytochemical, Nutritional and Physicochemical Characteristics of Fruits and Leaves from the Myrica L. Genus: A Systematic Review
Source: Int J Mol Sci. 2026 Sep 16;27(18):8237. doi: 10.3390/ijms27188237 (PMC13607124; doi:10.3390/ijms27188237)
Supplement: Supplementary file 1 [file ijms-27-08237-s001.zip › ijms-4484945-supplementary.pdf]

Review

Phytochemical, nutritional and physicochemical characteristics of fruits and leaves from the *Myrica* L. genus: A systematic review

Sofia G. Florença [1,2,5], Maria João Barroca [3,4], Maria João Lima [1], Cláudia Nunes [5*]

[1]CERNAS-IPV Research Centre, Polytechnic University of Viseu, 3504-510, Viseu, Portugal, mjoaolima@esav.ipv.pt (M.J.L), sofiaflorenca@outlook.com (S.G.F.)
[2] University of Aveiro, 3810-193 Aveiro, Portugal
[3] Unidade de I&D Química-Física Molecular, Department of Chemistry, University of Coimbra, 3004-535 Coimbra, Portugal, mjbarroca@esac.pt (M.J.B)
[4] Polytechnic Institute of Coimbra, Coimbra Agriculture School, Bencata, 3045-601 Coimbra, Portugal
[5] CICECO—Aveiro Institute of Materials, Department of Materials and Ceramic Engineering, University of Aveiro, 3810-193 Aveiro, Portugal, * claudianunes@ua.pt (C.N.)

Abstract

The *Myrica* L. genus represents a group of plants commonly known as actinorhizal plants, that have been used as food, medicine, and natural dyes. This article aims to systematically review the available recent scientific evidence identified through a defined search strategy on the phytochemical, physical, and nutritional characteristics of the fruits and leaves of the species from the *Myrica* L. genus. The search was conducted in three databases, following the Preferred Reporting of Items for Systematic Reviews and Meta-analysis statement guidelines. Studies published in English, as open-access articles, between 2014 and 2024, and addressing the aim of the review were included, resulting in 16 eligible articles. Concerning the physicochemical characteristics of the fruits, the research indicates variability among species and within the same species. The nutritional results are limited, with one study reporting carbohydrates and proteins as the main macronutrient components- 79.84% and 25.02% respectively, in *Myrica esculenta* fruits, although with limited interpretation. Furthermore, numerous phytochemicals have been identified and quantified, some with reported biological activities. For example, myricetin concentrations in *Myrica rubra* fruits ranged from 0.002 to 1.27 mg/g. In conclusion, this review highlights the nutritional and phytochemical potential of *Myrica* L. species and the wide range of chemical compounds reported, while acknowledging the limited number of studies and the methodological heterogeneity, among other limitations.

Keywords: *Myrica*; Fruits; Leaves; Phytochemical composition; Nutritional value

Abbreviations

CIRG	Colour Index for Red Grapes
DE	Dried extract
DW	Dry weight
FW	Fresh weight
GAE	Gallic acid equivalent
ME	*Myrica esculenta*
MF	*Myrica faya*
MN	*Myrica nagi*
MR	*Myrica rubra*
MYE	Myricitrin equivalent
ND	Not detected
QE	Quercetin equivalent
Q3E	Quercetin 3-rhamnoside equivalent
RE	Rutin equivalent

Fruits' phytochemical, physicochemical and nutritional composition

Table S1. Moisture, weight, edible rate, titratable acidity and pH in fruits.

Species of *Myrica* L.	Sub-species ǀ Variety	Reference	Moisture (%)	Weight (g)	Edible rate (%)	Titratable acidity (%)	pH
MR	Biqi	(Zhang et al., 2015) [1]		11.5 ± 0.53	95.39		
	Ciji	(Zhang et al., 2015) [1]		14.02 ± 0.34	96.50		
	Ding'ao	(Zhang et al., 2015) [1]		13.00 ± 0.54	96.46		
	Dongkui	(Zhang et al., 2015) [1]		27.62 ± 0.86	97.39		
		(Chen et al., 2020) [2]					2.92
	Dayexidi	(Zhang et al., 2015) [1]		13.68 ± 0.25	97.08		
	Fenhong	(Zhang et al., 2015) [1]		12.79 ± 0.52	95.78		
	Muye	(Chen et al., 2020) [2]					2.63
	Muyezhong	(Zhang et al., 2015) [1]		10.65 ± 0.22	94.84		
	Shenhongzh-ong	(Zhang et al., 2015) [1]		13.74 ± 0.51	96.07		
	Shuijing	(Zhang et al., 2015) [1]		9.04 ± 0.37	95.02		
	Shuimei	(Zhang et al., 2015) [1]		16.12 ± 0.4	95.72		
	Tanmei	(Zhang et al., 2015) [1]		8.81 ± 0.18	96.37		
	Wandao	(Zhang et al., 2015) [1]		12.39 ± 0.25	96.37		
	Wumei	(Zhang et al., 2015) [1]		11.58 ± 0.91	94.30		
	Wuzi	(Zhang et al., 2015) [1]		28.17 ± 0.97	95.67		
	Xiaoyexidi	(Zhang et al., 2015) [1]		11.82 ± 0.47	96.19		

	Zaodamei	(Zhang et al., 2015) [1]		17.95 ± 0.42	96.16		
	ZaoJia	(Chen et al., 2020) [2]					2.85
	Zaose	(Zhang et al., 2015) [1]		12.39 ± 0.29	96.69		
ME	–	(Ngurthankhumi et al., 2024) [3]	89.00 ± 1.00			1.38 ± 0.31	
	–	(Rymbai et al., 2023) [4]	86.75 ± 1.33	13.26 ± 1.83		3.32 ± 0.06	
MN	–	(Rymbai et al., 2023) [4]	83.62 ± 1.52	8.30 ± 0.56		2.45 ± 0.04	
MF	–	(Spínola et al., 2014) [5]					4.02

Table S2. Dry matter, total ash, juice and pulp content.

**Species of *Myrica* L.**	**Sub-species ǀ Variety**	**Reference**	**Dry matter (%)**	**Total ash (%)**	**Juice content (%)**	**Pulp content (%)**
ME	–	(Ngurthankhumi et al., 2024) [3]	11.00 ± 1.00	2.75 ± 0.08		
	–	(Rymbai et al., 2023) [4]			40.36 ± 1.47	84.67 ± 0.53
MN	–	(Rymbai et al., 2023) [4]			21.22 ± 0.92	84.12 ± 1.97

Table S3. Colour of fruits.

**Species of *Myrica* L.**	**Sub-species ǀ Variety**	**Reference**	**Colour**			
			**L* value**	**a* value**	**b* value**	**CIRG**
**MR**	Biqi	(Zhang et al., 2015) [1]				8.83 ± 0.24
	Ciji	(Zhang et al., 2015) [1]				8.58 ± 0.15

	Ding'ao	(Zhang et al., 2015) [1]				6.78 ± 0.18
	Dongkui	(Zhang et al., 2015) [1]				7.51 ± 0.18
	Dayexidi	(Zhang et al., 2015) [1]				7.65 ± 0.10
	Fenhong	(Zhang et al., 2015) [1]				4.41 ± 0.11
	Muyezhong	(Zhang et al., 2015) [1]				6.06 ± 0.13
	Shenhongzhong	(Zhang et al., 2015) [1]				4.24 ± 0.11
	Shuijing	(Zhang et al., 2015) [1]				1.96 ± 0.02
	Shuimei	(Zhang et al., 2015) [1]				5.92 ± 0.06
	Tanmei	(Zhang et al., 2015) [1]				7.71 ± 0.67
	Wandao	(Zhang et al., 2015) [1]				12.39 ± 0.24
	Wumei	(Zhang et al., 2015) [1]				6.87 ± 0.17
	Wuzi	(Zhang et al., 2015) [1]				6.32 ± 0.11
	Xiaoyexidi	(Zhang et al., 2015) [1]				7.75 ± 0.12
	Zaodamei	(Zhang et al., 2015) [1]				6.40 ± 0.16
	Zaose	(Zhang et al., 2015) [1]				5.39 ± 0.10
**ME**	–	(Rymbai et al., 2023) [4]	54.2 ± 6.36	15.7 ± 4.29	29.6 ± 2.05	
**MN**	–	(Rymbai et al., 2023) [4]	25.6 ± 1.57	28.5 ± 1.51	9.3 ± 0.67	

Table S4. Fibre content.

Species of *Myrica* L.	Sub-species I Variety	Reference	Crude fibre (%)	Lignin (%)	Cellulose (mg/100 g)	Hemicellulose (%)
ME	–	(Ngurthankhumi et al., 2024) [3]	4.72 ± 0.44	2.76 ± 0.01	67.39 ± 0.65	4.84 ± 0.01

Table S5. Sugar content and total soluble solids (TSS).

Species of *Myrica* L.	Sub-species I Variety	Reference	Total sugar (%)	Reducing sugar (%)	Non-reducing sugar (%)	Sucrose (mg/g)	Fructose (mg/g)	Glucose (mg/g)	TSS (ºB)
MR	Biqi	(Zhang et al., 2015) [1]				54.36 ± 0.93	11.55 ± 0.78	9.64 ± 0.57	9.51 ± 0.19
	Ciji	(Zhang et al., 2015) [1]				61.9 ± 1.24	13.51 ± 1.22	11.26 ± 1.02	11.41 ± 0.21
	Ding'ao	(Zhang et al., 2015) [1]				43.87 ± 2.59	11.16 ± 0.54	9.17 ± 0.9	8.89 ± 0.12
	Dongkui	(Zhang et al., 2015) [1]				65.44 ± 0.71	13.96 ± 0.54	11.96 ± 0.66	11.67 ± 0.17
		(Chen et al., 2020) [2]				50.00 – 60.00	5.00 – 10.00	5.00 – 10.00	
	Dayexidi	(Zhang et al., 2015) [1]				65.85 ± 1.38	14.64 ± 1.07	11.85 ± 0.72	11.55 ± 0.21
	Fenhong	(Zhang et al., 2015) [1]				54.18 ± 2.32	10.84 ± 0.92	9.33 ± 0.45	9.28 ± 0.1
	Muye	(Chen et al., 2020) [2]				40.00 – 50.00	5.00 – 10.00	5.00 – 10.00	
	Muyezhong	(Zhang et al., 2015) [1]				56.72 ± 2.42	14.66 ± 0.32	12.27 ± 0.97	10.74 ± 0.2
	Shenhongzh-ong	(Zhang et al., 2015) [1]				57.72 ± 1.34	12.49 ± 1.5	10.66 ± 0.31	9.68 ± 0.2

	Shuijing	(Zhang et al., 2015) [1]				40.41 ± 0.27	11.27 ± 0.91	9.98 ± 0.65	8.74 ± 0.22
	Shuimei	(Zhang et al., 2015) [1]				56.15 ± 1.08	13.07 ± 1.5	11.22 ± 1.32	11.1 ± 0.21
	Tanmei	(Zhang et al., 2015) [1]				65.6 ± 2.44	13.34 ± 0.80	12.28 ± 1.33	10.48 ± 0.15
	Wandao	(Zhang et al., 2015) [1]				52.2 ± 3.34	10.64 ± 0.93	9.12 ± 0.58	10.48 ± 0.15
	Wumei	(Zhang et al., 2015) [1]				54.54 ± 2.23	14.65 ± 0.17	11.90 ± 0.52	11.24 ± 0.12
	Wuzi	(Zhang et al., 2015) [1]				55.52 ± 2.14	12.64 ± 0.96	11.43 ± 0.82	9.52 ± 0.21
	Xiaoyexidi	(Zhang et al., 2015) [1]				59.71 ± 1.04	13.46 ± 0.60	11.94 ± 0.96	10.77 ± 0.21
	Zaodamei	(Zhang et al., 2015) [1]				59.01 ± 3.44	10.64 ± 1.78	9.21 ± 1.28	10.64 ± 0.20
	ZaoJia	(Chen et al., 2020) [2]				40.00 – 50.00	5.00 – 10.00	5.00 – 10.00	
	Zaose	(Zhang et al., 2015) [1]				54.19 ± 1.68	13.56 ± 0.61	12.33 ± 0.62	9.65 ± 0.12
ME	–	(Ngurthankhumi et al., 2024) [3]	17.59 ± 2.31	10.55 ± 0.56	6.69 ± 1.67				12.03 ± 0.25
	–	(Rymbai et al., 2023) [4]	4.27 ± 0.08	2.93 ± 0.03					5.83 ± 0.30
MN	–	(Rymbai et al., 2023) [4]	6.83 ± 0.05	3.08 ± 0.10					6.76 ± 0.43
MF	–	(Spínola et al., 2014) [5]							14.87

Table S6. Organic acid and vitamin content.

Species of *Myrica* L.	Sub-species I Variety	Reference	Ascorbic acid (mg/100 g)	L-Ascorbic acid (L-AA) (mg/100 g)	Vitamin E (mg/100 g)	Citric Acid (mg/g)	Malic Acid (mg/ g)
MR	Biqi	(Zhang et al., 2015) [1]				9.17 ± 0.2	0.68 ± 0.01
	Ciji	(Zhang et al., 2015) [1]				10.14 ± 0.09	0.46 ± 0.04
	Ding'ao	(Zhang et al., 2015) [1]				7.84 ± 0.07	1.04 ± 0.00
	Dongkui	(Zhang et al., 2015) [1]				10.51 ± 0.13	0.47 ± 0.03

		(Chen et al., 2020) [2]	4.20				
	Dayexidi	(Zhang et al., 2015) [1]				8.27 ± 0.1	1.16 ± 0.04
	Fenhong	(Zhang et al., 2015) [1]				9.49 ± 0.15	0.39 ± 0.03
	Muye	(Chen et al., 2020) [2]	2.12				
	Muyezhon g	(Zhang et al., 2015) [1]				12.72 ± 0.08	0.50 ± 0.00
	Shenhongz h-ong	(Zhang et al., 2015) [1]				10.55 ± 0.15	0.55 ± 0.01
	Shuijing	(Zhang et al., 2015) [1]				22.06 ± 0.23	0.12 ± 0.00
	Shuimei	(Zhang et al., 2015) [1]				11.11 ± 0.21	0.88 ± 0.03
	Tanmei	(Zhang et al., 2015) [1]				10.64 ± 0.29	0.48 ± 0.00
	Wandao	(Zhang et al., 2015) [1]				8.45 ± 0.06	1.04 ± 0.01
	Wumei	(Zhang et al., 2015) [1]				10.5 ± 0.59	0.04 ± 0.00
	Wuzi	(Zhang et al., 2015) [1]				10.94 ± 0.17	0.43 ± 0.01
	Xiaoyexidi	(Zhang et al., 2015) [1]				8.05 ± 0.10	0.75 ± 0.00
	Zaodamei	(Zhang et al., 2015) [1]				10.43 ± 0.06	0.71 ± 0.03
	ZaoJia	(Chen et al., 2020) [2]	1.79				
	Zaose	(Zhang et al., 2015) [1]				9.53 ± 0.17	1.05 ± 0.00
ME	–	(Ngurthankhumi et al., 2024) [3]	60.68 ± 4.65		23.27 ± 0.61		
	–	(Rymbai et al., 2023) [4]	22.50 – 30.00				
MN	–	(Rymbai et al., 2023) [4]	7.50 – 15.00				
MF	–	(Spínola et al., 2014) [5]		48.42 ± 1.93			

Table S7. Mineral content.

**Elements**	**Species of *Myrica* L.**	
	**ME** (Ngurthankhumi et al., 2024) [3]	**MF** * (Llorent-Martinez et al., 2017) [6] **(µg/g)**
Aluminium (Al)		80.00 ± 50.00
Antimony (Sb)		ND
Ardennite (As)		ND
Barium (Ba)		0.50 ± 0.30
Cadmium (Cd)		ND
Calcium (Ca)	79.93 ± 10.67 [b]	900.00 ±100.00
Chromium (Cr)		0.60 ± 0.50
Cobalt (Co)	0.17 ± 0.02 [b]	0.20 ± 0.10
Copper (Cu)	1.09 ± 0.01 [b]	1.60 ± 0.50
Iron (Fe)	13.23 ± 0.29 [b]	110.00 ± 50.00
Lead (Pb)		ND
Magnesium (Mg)	311.55 ± 7.27 [b]	550.00 ± 100.00
Manganese (Mn)	27.66 ± 1.04 [b]	9.00 ± 3.00
Mercury (Hg)		ND
Molybdenum Mo)		0.11 ±0.05
Nickel (Ni)		1.60 ±0.60
Nitrogen (N)	6.85 ± 0.19 [a]	
Phosphorus (P)	0.10 ± 0.01 [a]	330.00 ± 50.00
Potassium (K)	1.61 ± 0.03 [a]	4400.00 ± 500.00
Selenium (Se)		ND
Silver (Ag)		ND
Sodium (Na)	48.13 ± 2.75 [b]	2000.00 ± 400.00

Thallium (Tl)		ND
Tin (Sn)		ND
Titanium Mineral (Ti)		20.00 ± 10.00
Vanadium (V)		0.30 ± 0.20
Zinc (Zn)	3.46 ± 0.07 [b]	4.40 ± 0.50

[a] % | [b] mg/100 g | ND = Not Detected

* Digested fruit

Table S8. Total phenolic, total flavonoids, and total flavonols content determined by spectrophotometry.

Species of *Myrica* L.	Sub-species | Variety | Location	Maturation stage	Ref.	Total phenolic	Total flavonoids	Total flavonols (mg QE/g)
MR	Biqi	–	(Zhang et al., 2015) [1]	2.53 [a]	1.91 [c]	
	Ciji	–	(Zhang et al., 2015) [1]	2.15 [a]	1.48 [c]	
	Ding'ao	–	(Zhang et al., 2015) [1]	1.95 [a]	1.26 [c]	
	Dongkui	–	(Zhang et al., 2015) [1]	1.47 [a]	1.05 [c]	
	Dayexidi	–	(Zhang et al., 2015) [1]	1.91 [a]	1.25 [c]	
	Fenhong	–	(Zhang et al., 2015) [1]	1.33 [a]	1.01 [c]	
	Muyezhong	–	(Zhang et al., 2015) [1]	2.31 [a]	1.80 [c]	
	Shenhong-zhong	–	(Zhang et al., 2015) [1]	1.35 [a]	1.12 [c]	
	Shuijing	–	(Zhang et al., 2015) [1]	1.31 [a]	0.85 [c]	
	Shuimei	–	(Zhang et al., 2015) [1]	1.55 [a]	0.88 [c]	

	Tanmei	–	(Zhang et al., 2015) [1]	2.07 [a]	1.82 [c]	
	Wandao	–	(Zhang et al., 2015) [1]	2.38 [a]	1.60 [c]	
	Wumei	–	(Zhang et al., 2015) [1]	1.66 [a]	1.29 [c]	
	Wuzi	–	(Zhang et al., 2015) [1]	2.04 [a]	1.29 [c]	
	Xiaoyexidi	–	(Zhang et al., 2015) [1]	1.70 [a]	1.25 [c]	
	Zaodamei	–	(Zhang et al., 2015) [1]	1.84 [a]	1.24 [c]	
	Zaose	–	(Zhang et al., 2015) [1]	1.35 [a]	0.85 [c]	
	–	–	(Xia et al., 2023) [7]	4.90 [a]		
	–	10 days after pollination	(Ren et al., 2019) [8]		50.00 – 60.00 [d]	
	–	20 days after pollination	(Ren et al., 2019) [8]		50.00 – 60.00 [d]	
	–	30 days after pollination	(Ren et al., 2019) [8]		40.00 – 50.00 [d]	
	–	40 days after pollination	(Ren et al., 2019) [8]		30.00 – 40.00 [d]	
	–	50 days after pollination	(Ren et al., 2019) [8]		20.00 – 30.00 [d]	
ME	–	–	(Rymbai et al., 2023) [4]	10.00 – 15.00 [a]	4.50 – 6.00 [e]	1.00 – 2.00
MN	–	–	(Rymbai et al., 2023) [4]	10.00 – 15.00 [a]	3.00 – 4.50 [e]	1.00 – 2.00

MF	–	–	(Spínola et al., 2014) [5]	52.30 [b*]	42.10 [f]	
	Machico	–	(Spínola et al., 2019) [9]	44.86 ± 1.04 [b]	8.02 ± 0.39 [f]	
	Faial	–	(Spínola et al., 2019) [9]	43.04 ± 1.50 [b]	7.73 ± 0.18 [f]	
	Arco de São Jorge	–	(Spínola et al., 2019) [9]	59.09 ± 2.81 [b]	8.51 ± 0.29 [f]	
	Boaventura	–	(Spínola et al., 2019) [9]	62.94 ± 1.93 [b]	9.24 ± 0.43 [f]	
	Seixal	–	(Spínola et al., 2019) [9]	50.21 ± 2.69 [b]	7.47 ± 0.23 [f]	
	Ribeira da Janela	–	(Spínola et al., 2019) [9]	47.20 ± 1.89 [b]	7.66 ± 0.18 [f]	
	Porto Moniz	–	(Spínola et al., 2019) [9]	51.18 ± 0.84 [b]	6.68 ± 0.24 [f]	
	Terceira	–	(Spínola et al., 2019) [9]	53.86 ± 1.92 [b]	10.74 ± 0.52 [f]	

[a] mg gallic acid equivalent (GAE)/g | [b] mg GAE/g dried extract (DE) | [c] mg rutin equivalent (RE)/g fresh weight (FW) | [d] mg/g dry weight (DW) | [e] mg quercetin equivalent (QE)/g FW | [f] mg RE/g DE * Corrected TPC value (subtracted L-AA contribution)

Table S9. Total individual phenolic, total phenolic, total flavonols, total flavanols, and total flavones content determined by HPLC.

Species of *Myrica* L.	Sub-species | Variety | Location	Reference	Total individual phenolic	Total phenolic acids (mg/100 g DW)	Total flavonols	Total flavanols	Total flavones
MF	–	(Spínola et al., 2014) [5]	820.11 ± 46.71 [a]	45.73 ± 0.97	268.81 ± 9.43 [a]	37.80 ± 1.22 [a]	82.69 ± 4.79 [a]
	Machico	(Spínola et al., 2019) [9]	35.20 ± 0.66 [b]		4.34 ± 0.08 [b]	0.40 ± 0.01 [b]	0.25 ± 0.01 [b]
	Faial	(Spínola et al., 2019) [9]	37.88 ± 0.77 [b]		3.44 ± 0.15 [b]	2.29 ± 0.07 [b]	
	Arco de São Jorge	(Spínola et al., 2019) [9]	42.74 ± 0.90 [b]		3.61 ± 0.08 [b]	0.66 ± 0.02 [b]	

	Boaventura	(Spínola et al., 2019) [9]	43.63 ± 0.78 [b]		2.93 ± 0.05 [b]	0.74 ± 0.02 [b]	
	Seixal	(Spínola et al., 2019) [9]	38.12 ± 0.92 [b]		3.57 ± 0.07 [b]	0.39 ± 0.01 [b]	
	Ribeira da Janela	(Spínola et al., 2019) [9]	34.52 ± 0.57 [b]		4.57 ± 0.13 [b]	0.80 ± 0.09 [b]	
	Porto Moniz	(Spínola et al., 2019) [9]	37.88 ± 0.85 [b]		3.98 ± 0.08 [b]	0.86 ± 0.02 [b]	0.11 ± 0.01 [b]
	Terceira	(Spínola et al., 2019) [9]	40.48 ± 0.25 [b]		6.26 ± 0.20 [b]	0.62 ± 0.01 [b]	0.21 ± 0.01 [b]

[a] mg/100 g DW | [b] mg/g DE

Table S10. Total anthocyanins and total carotenoids content determined by spectrophotometry.

**Species of *Myrica* L.**	**Sub-species | Variety**	**Maturation stage**	**Reference**	**Total anthocyanins (mg/g)**	**Total carotenoids (mg/g)**
MR	DongKui	Veraison	(Chen et al., 2020) [2]	0.17	7.00 – 8.00
		Mature	(Chen et al., 2020) [2]		5.00 – 7.00
	Muye	Veraison	(Chen et al., 2020) [2]	0.30	8.00 – 10.00
		Mature	(Chen et al., 2020) [2]		5.00 – 7.00
	ZaoJia	Veraison	(Chen et al., 2020) [2]	0.28	10.00 – 12.00
		Mature	(Chen et al., 2020) [2]		10.00 – 12.00
	–		(Yun et al., 2019) [10]	273.00	
ME	–		(Ngurthankhumi et al., 2024) [3]	3.10	0.01
	–		(Rymbai et al., 2023) [4]	0.70 – 1.00	0.10 – 0.12

MN	–		(Rymbai et al., 2023) [4]	1.50 – 1.70	0.13 – 0.15

Table S11. Total anthocyanins, total ellagitannins, total hydroxycinnamic acids, and total hydroxybenzoic acids content determined by HPLC.

Species of *Myrica* L.	Sub-species ∣ Variety ∣ Location	Reference	Total anthocyanins	Total ellagitannins (mg/g DE)	Total hydroxycinnamic acids (mg/g DE)	Total hydroxybenzoic acids (mg/g DE)
MF	–	(Spínola et al., 2014) [5]	385.09 ± 6.61 [a]			
	Machico	(Spínola et al., 2019) [9]	27.24 ± 0.10 [b]	0.83 ± 0.02	0.40 ± 0.01	1.75 ± 0.05
	Faial	(Spínola et al., 2019) [9]	28.51 ± 0.54 [b]	0.77 ± 0.01	1.12 ± 0.03	1.75 ± 0.03
	Arco de São Jorge	(Spínola et al., 2019) [9]	36.13 ± 0.27 [b]	0.82 ± 0.01	0.51 ± 0.01	1.90 ± 0.03
	Boaventura	(Spínola et al., 2019) [9]	35.11 ± 0.21 [b]	1.14 ± 0.02	0.95 ± 0.023	1.87 ± 0.04
	Seixal	(Spínola et al., 2019) [9]	30.21 ± 0.07 [b]	1.35 ± 0.03	0.59 ± 0.02	2.01 ± 0.05
	Ribeira da Janela	(Spínola et al., 2019) [9]	25.64 ± 0.32 [b]	1.09 ± 0.04	0.69 ± 0.01	1.73 ± 0.02
	Porto Moniz	(Spínola et al., 2019) [9]	29.75 ± 0.69 [b]	1.04 ± 0.03	0.86 ± 0.02	1.28 ± 0.01
	Terceira	(Spínola et al., 2019) [9]	29.32 ± 0.13 [b]	1.24 ± 0.02	0.87 ± 0.01	1.96 ± 0.07

[a] mg/100 g DW ∣ [b] mg/g DE

Table S12. Hydroxycinnamic acids (Caffeoylisocitrate, 5-*O*-caffeoylquininc acid, Caffeic acid-*O*-hexoside and Dihydro-Co-*O*-hexoside).

Species of *Myrica* L.	Sub-species ǀ Variety ǀ Location	Reference	Caffeoylisocitrate (mg/g DE)	5-*O*-caffeoylquininc acid (mg/g DE)	Caffeic acid-*O*-hexoside (mg/100 g DW)	Dihydro-Co-*O*-hexoside (mg/g DE)
MF	–	(Spínola et al., 2014) [5]			45.73 ± 0.97	
	Machico	(Spínola et al., 2019) [9]				
	Faial	(Spínola et al., 2019) [9]	0.41 ± 0.01			
	Arco de São Jorge	(Spínola et al., 2019) [9]				
	Boaventura	(Spínola et al., 2019) [9]	0.12 ± 0.01	0.29 ± 0.01		
	Seixal	(Spínola et al., 2019) [9]		0.21 ± 0.01		
	Ribeira da Janela	(Spínola et al., 2019) [9]	0.20 ± 0.01			
	Porto Moniz	(Spínola et al., 2019) [9]	0.33 ± 0.01			
	Terceira	(Spínola et al., 2019) [9]				0.08 ± 0.01

Table S13. Hydroxycinnamic acids (Benzoyl-*p*-tri-coumaroyl-2,7-anhydro-3-deoxy-2-octulopyranosonic acid, Coumaric acid-*O*-hexoside, *p*-coumaric acid and Ferulic acid).

**Species of *Myrica* L.**	**Sub-species ǀ Variety ǀ Location**	**Reference**	**Benzoyl-*p*-tri-coumaroyl-2,7-anhydro-3-deoxy-2-octulopyranosonic acid (mg/g DE)**	**Coumaric acid-*O*-hexoside (mg/g DE)**	***p*-coumaric acid (μg/g FW)**	**Ferulic acid (μg/g FW)**
MR	–	(Xia et al., 2023) [7]			0.73	0.61
MF	Machico	(Spínola et al., 2019) [9]	0.39 ± 0.01	0.01 ± 0.01		
	Faial	(Spínola et al., 2019) [9]	0.69 ± 0.02	0.02 ± 0.01		
	Arco de São Jorge	(Spínola et al., 2019) [9]	0.51 ± 0.01			
	Boaventura	(Spínola et al., 2019) [9]	0.54 ± 0.01			
	Seixal	(Spínola et al., 2019) [9]	0.38 ± 0.02			
	Ribeira da Janela	(Spínola et al., 2019) [9]	0.48 ± 0.01	0.01 ± 0.01		
	Porto Moniz	(Spínola et al., 2019) [9]	0.53 ± 0.01			
	Terceira	(Spínola et al., 2019) [9]	0.79 ± 0.01			

Table S14. Hydroxybenzoic acids (Galloyl-*O*-hexoside, Galloylquinic acid, Digalloyl-*O*-hexoside and Trigalloyl glucose).

**Species of *Myrica* L.**	**Sub-species I Variety I Location**	**Reference**	**Galloyl-*O*-hexoside** (mg/g DE)	**Galloylquinic acid** (mg/g DE)	**Digalloyl-*O*-hexoside** (mg/g DE)	**Trigalloyl glucose** (mg/g DE)
MF	Machico	(Spínola et al., 2019) [9]	1.36		0.39 ± 0.01	
	Faial	(Spínola et al., 2019) [9]	1.24		0.27 ± 0.08	0.24 ± 0.01
	Arco de São Jorge	(Spínola et al., 2019) [9]	1.50	0.20 ± 0.01	0.20 ± 0.01	
	Boaventura	(Spínola et al., 2019) [9]	1.61		0.20 ± 0.01	0.06 ± 0.01
	Seixal	(Spínola et al., 2019) [9]	1.74		0.27 ± 0.01	
	Ribeira da Janela	(Spínola et al., 2019) [9]	1.52		0.21 ± 0.01	
	Porto Moniz	(Spínola et al., 2019) [9]	1.18		0.10 ± 0.01	
	Terceira	(Spínola et al., 2019) [9]	1.22		0.69 ± 0.01	

Table S15. Hydroxybenzoic acids (Methyl gallate derivative, Gallic acid, *p*-hydroxybenzoic acid and Protocatechuic acid).

**Species of *Myrica* L.**	**Sub-species I Variety I Location**	**Reference**	**Methyl gallate derivative** (mg/g DE)	**Gallic acid** (µg/g FW)	**p-hydroxybenzoic acid** (µg/g FW)	**Protocatechuic acid** (µg/g FW)
MR	–	(Xia et al., 2023) [7]		18.83	1.05	0.75
MF	–	(Spínola et al., 2014) [5]				
	Terceira	(Spínola et al., 2019) [9]	0.05 ± 0.01			

Table S16. Flavonols (Myricetin, Myricetin-*O*-(*O*-galloyl)hexoside, Myricetin-*O*-hexoside, Myricetin-*O*-deoxyhexoside, Myricetin-*O*-(*O*-galloyl)deoxyhexoside and Myricetin-*O*-(galloyl)hexoside).

**Species of *Myrica* L.**	**Sub-species ǀ Variety ǀ Location**	**Maturation stage**	**Reference**	**Myricetin** (mg/g)	**Myricetin-*O*-(*O*-galloyl)hexoside**	**Myricetin-*O*-hexoside**	**Myricetin-*O*-deoxyhexoside** (mg/100 g DW)	**Myricetin-*O*-(*O*-galloyl) deoxyhexoside** (mg/100 g DW)	**Myricetin-*O*-(galloyl) hexoside** (mg/g DE)
MR	DongKui	Veraison	(Chen et al., 2020) [2]	0.07					
		Mature	(Chen et al., 2020) [2]	0.06					
	Muye	Veraison	(Chen et al., 2020) [2]	0.26					
		Mature	(Chen et al., 2020) [2]	1.27					
	ZaoJia	Veraison	(Chen et al., 2020) [2]	0.10					
		Mature	(Chen et al., 2020) [2]	0.88					
	–	–	(Xia et al., 2023) [7]	0.002	ND				
MF	–	–	(Spínola et al., 2014) [5]			80.98 ± 2.65 [a]	44.20 ± 1.23	59.51 ± 2.64	
	Machico	–	(Spínola et al., 2019) [9]			0.22 ± 0.01 [b]			0.14 ± 0.01
	Faial	–	(Spínola et al., 2019) [9]			0.16 ± 0.01 [b]			0.21 ± 0.01

	Arco de São Jorge	–	(Spínola et al., 2019) [9]			0.26 ± 0.01 [b]			0.14 ± 0.01
	Boaventura	–	(Spínola et al., 2019) [9]			0.25 ± 0.01 [b]			0.09 ± 0.01
	Seixal	–	(Spínola et al., 2019) [9]			0.29 ± 0.01 [b]			0.10 ± 0.01
	Ribeira da Janela	–	(Spínola et al., 2019) [9]			0.24 ± 0.01 [b]			0.12 ± 0.01
	Porto Moniz	–	(Spínola et al., 2019) [9]			0.12 ± 0.01 [b]			0.09 ± 0.01
	Terceira	–	(Spínola et al., 2019) [9]			0.25 ± 0.01 [b]			0.12 ± 0.01

[a] mg/100 g DW | [b] mg/g DE | ND = Not Detected

Table S17. Flavonols (Dimethyl-myricetin-*O*-pentoside, Myricetin-*O*-(galloyl)deoxyhexoside, Myricitrin, Myricetin-*O*-pentoside, Myricetin-3-*O*-rhamnoside and Myricetin deoxyhexoside-gallate).

**Species of *Myrica* L.**	**Sub-species | Variety | Location**	**Reference**	**Dimethyl-myricetin-*O*-pentoside** (mg/g DE)	**Myricetin-*O*-(galloyl)deoxyhexoside** (mg/g DE)	**Myricitrin** (mg/g DE)	**Myricetin-*O*-pentoside** (mg/g DE)	**Myricetin-3-*O*-rhamnoside** (µg/g FW)	**Myricetin deoxyhexoside-gallate** (µg/g FW)
MR	Biqi	(Zhang et al., 2015) [1]					50.33 ± 2.88	1.87 ± 0.18
	Ciji	(Zhang et al., 2015) [1]					18.38 ± 1.45	1.08 ± 0.10
	Ding'ao	(Zhang et al., 2015) [1]					61.50 ± 3.72	1.21 ± 0.06
	Dongkui	(Zhang et al., 2015) [1]					28.09 ± 1.84	1.79 ± 0.08
	Dayexidi	(Zhang et al., 2015) [1]					20.27 ± 0.33	1.14 ± 0.09
	Fenhong	(Zhang et al., 2015) [1]					14.49 ± 0.61	1.10 ± 0.08

	Muyezhong	(Zhang et al., 2015) [1]					40.40 ± 1.52	2.00 ± 0.19
	Shenhong-zhong	(Zhang et al., 2015) [1]					19.73 ± 0.63	1.13 ± 0.07
	Shuijing	(Zhang et al., 2015) [1]					36.04 ± 1.33	3.50 ± 0.18
	Shuimei	(Zhang et al., 2015) [1]					39.15 ± 2.86	1.70 ± 0.10
	Tanmei	(Zhang et al., 2015) [1]					27.85 ± 1.47	1.24 ± 0.08
	Wandao	(Zhang et al., 2015) [1]					40.28 ± 3.64	4.37 ± 0.38
	Wumei	(Zhang et al., 2015) [1]					31.37 ± 1.91	5.49 ± 0.15
	Wuzi	(Zhang et al., 2015) [1]					38.53 ± 1.55	1.32 ± 0.09
	Xiaoyexidi	(Zhang et al., 2015) [1]					48.74 ± 0.28	2.55 ± 0.15
	Zaodamei	(Zhang et al., 2015) [1]					44.10 ± 2.52	0.96 ± 0.05
	Zaose	(Zhang et al., 2015) [1]					10.73 ± 0.71	4.41 ± 0.28
MF	Machico	(Spínola et al., 2019) [9]		0.42 ± 0.01	1.36 ± 0.02			
	Faial	(Spínola et al., 2019) [9]		0.38 ± 0.01	1.09 ± 0.02	0.49 ± 0.01		
	Arco de São Jorge	(Spínola et al., 2019) [9]		0.35 ± 0.01	1.13 ± 0.03			
	Boaventura	(Spínola et al., 2019) [9]			1.20 ± 0.02			
	Seixal	(Spínola et al., 2019) [9]		0.30 ± 0.02	1.53 ± 0.02			
	Ribeira da Janela	(Spínola et al., 2019) [9]		0.38 ± 0.01	1.37 ± 0.03			
	Porto Moniz	(Spínola et al., 2019) [9]	0.25 ± 0.01		1.38 ± 0.05	0.11 ± 0.01		
	Terceira	(Spínola et al., 2019) [9]		0.19 ± 0.01	1.23 ± 0.02	0.18 ± 0.01		

Table S18. Flavonols (Kaempferol-*O*-hexoside, Kaempferol-*O*-rhamnoside, Kaemferol-3-*O*-galactoside, Kaemferol-3-*O*-glucoside, Kaempferol and Dihydro-kaempferol-*O*-hexoside).

**Species of *Myrica* L.**	**Sub-species ǀ Variety ǀ Location**	**Reference**	**Kaempferol-*O*-hexoside**	**Kaempferol-*O*-rhamnoside**	**Kaemferol-3-*O*-galactoside (μg/g FW)**	**Kaemferol-3-*O*-glucoside (μg/g FW)**	**Kaempferol (μg/g FW)**	**Dihydro-kaempferol-*O*-hexoside (mg/g DE)**
MR	Biqi	(Zhang et al., 2015) [1]			4.26 ± 0.33	4.32 ± 0.19		
	Ciji	(Zhang et al., 2015) [1]			2.11 ± 0.05	1.59 ± 0.07		
	Ding'ao	(Zhang et al., 2015) [1]			ND	0.32 ± 0.05		
	Dongkui	(Zhang et al., 2015) [1]			ND	0.69 ± 0.03		
	Dayexidi	(Zhang et al., 2015) [1]			0.63 ± 0.03	1.1 ± 0.04		
	Fenhong	(Zhang et al., 2015) [1]			ND	0.8 ± 0.02		
	Muyezhong	(Zhang et al., 2015) [1]			ND	1.09 ± 0.04		
	Shenhong-zhong	(Zhang et al., 2015) [1]			ND	0.98 ± 0.00		
	Shuijing	(Zhang et al., 2015) [1]			ND	1.71 ± 0.06		
	Shuimei	(Zhang et al., 2015) [1]			ND	0.71 ± 0.03		
	Tanmei	(Zhang et al., 2015) [1]			2.20 ± 0.08	2.42 ± 0.15		
	Wandao	(Zhang et al., 2015) [1]			1.19 ± 0.08	1.65 ± 0.13		
	Wumei	(Zhang et al., 2015) [1]			0.21 ± 0.01	3.91 ± 0.07		
	Wuzi	(Zhang et al., 2015) [1]			ND	0.26 ± 0.01		
	Xiaoyexidi	(Zhang et al., 2015) [1]			0.37 ± 0.04	0.35 ± 0.04		
	Zaodamei	(Zhang et al., 2015) [1]			1.34 ± 0.04	0.36 ± 0.03		

	Zaose	(Zhang et al., 2015) [1]			2.35 ± 0.01	0.25 ± 0.01		
	–	(Xia et al., 2023) [7]					0.87	
MF	–	(Spínola et al., 2014) [5]	8.44 ± 0.46 [a]	ND				
	Machico	(Spínola et al., 2019) [9]	0.75 [b]					
	Faial	(Spínola et al., 2019) [9]	0.26 ± 0.01 [b]					
	Arco de São Jorge	(Spínola et al., 2019) [9]	0.25 ± 0.02 [b]					
	Boaventura	(Spínola et al., 2019) [9]	0.52 [b]					
	Seixal	(Spínola et al., 2019) [9]	0.46 [b]					
	Ribeira da Janela	(Spínola et al., 2019) [9]	0.19 ± 0.01 [b]					
	Porto Moniz	(Spínola et al., 2019) [9]	0.41 [b]					
	Terceira	(Spínola et al., 2019) [9]	0.14 ± 0.01 [b]					0.29 ± 0.01

[a] mg/100 g DW | [b] mg/g DE | ND = Not detected

Table S19. Flavonols (Kaempferol-*O*-(galloyl)hexoside, Ellagic acid-*O*-pentoside, Quercetin-3-*O*-galactoside, Quercetin-3- *O*-glucoside, Quercetin-3-*O*-rhamnoside and Quercetin-*O*-hexoside).

**Species of *Myrica* L.**	**Sub-species ǀ Variety ǀ Location**	**Reference**	**Kaempferol-*O*-(galloyl)hexoside** (mg/g DE)	**Ellagic acid-*O*-pentoside** (mg/g DE)	**Quercetin-3-*O*-galactoside** (μg/g FW)	**Quercetin-3-*O*-glucoside** (μg/g FW)	**Quercetin-3-*O*-rhamnoside** (μg/g FW)	**Quercetin-*O*-hexoside** (mg/g DE)
MR	Biqi	(Zhang et al., 2015) [1]			74.47 ± 4.02	9.11 ± 0.46	49.70 ± 2.03	
	Ciji	(Zhang et al., 2015) [1]			35.07 ± 1.07	4.03 ± 0.23	20.15 ± 1.77	
	Ding'ao	(Zhang et al., 2015) [1]			0.19 ± 0.05	3.53 ± 0.09	51.73 ± 0.68	
	Dongkui	(Zhang et al., 2015) [1]			0.26 ± 0.02	0.85 ± 0.05	6.89 ± 0.3	
	Dayexidi	(Zhang et al., 2015) [1]			26.74 ± 0.45	5.83 ± 0.19	18.14 ± 0.29	
	Fenhong	(Zhang et al., 2015) [1]			0.25 ± 0.04	0.57 ± 0.05	8.37 ± 0.32	
	Muyezhong	(Zhang et al., 2015) [1]			0.67 ± 0.14	3.96 ± 0.25	30.38 ± 0.75	
	Shenhong-zhong	(Zhang et al., 2015) [1]			0.44 ± 0.04	0.79 ± 0.05	11.18 ± 0.19	
	Shuijing	(Zhang et al., 2015) [1]			0.16 ± 0.01	0.73 ± 0.09	6.94 ± 0.60	
	Shuimei	(Zhang et al., 2015) [1]			0.21 ± 0.01	0.98 ± 0.05	10.36 ± 0.43	
	Tanmei	(Zhang et al., 2015) [1]			39.93 ± 1.71	5.00 ± 0.1	31.16 ± 2.26	
	Wandao	(Zhang et al., 2015) [1]			0.97 ± 0.11	3.46 ± 0.32	18.52 ± 1.61	
	Wumei	(Zhang et al., 2015) [1]			0.48 ± 0.02	1.03 ± 0.08	13.82 ± 0.43	
	Wuzi	(Zhang et al., 2015) [1]			0.28 ± 0.01	1.77 ± 0.08	19.29 ± 0.68	
	Xiaoyexidi	(Zhang et al., 2015) [1]			16.52 ± 0.29	1.76 ± 0.13	13.68 ± 0.21	

	Zaodamei	(Zhang et al., 2015) [1]			11.69 ± 0.48	1.53 ± 0.15	14.82 ± 0.6	
	Zaose	(Zhang et al., 2015) [1]			5.89 ± 0.24	0.13 ± 0.01	3.31 ± 0.19	
MF	Machico	(Spínola et al., 2019) [9]	0.35 ± 0.01	0.16 ± 0.01				0.07 ± 0.01
	Faial	(Spínola et al., 2019) [9]	0.47 ± 0.03					
	Arco de São Jorge	(Spínola et al., 2019) [9]	0.43 ± 0.01	0.06 ± 0.01				0.18 ± 0.01
	Boaventura	(Spínola et al., 2019) [9]						0.12 ± 0.01
	Seixal	(Spínola et al., 2019) [9]	0.27 ± 0.01					0.45 ± 0.01
	Ribeira da Janela	(Spínola et al., 2019) [9]	0.82 ± 0.01					0.17 ± 0.01
	Porto Moniz	(Spínola et al., 2019) [9]		0.11 ± 0.01				0.07 ± 0.01
	Terceira	(Spínola et al., 2019) [9]	0.84 ± 0.01	0.22 ± 0.01				0.28 ± 0.01

Table S20. Flavonols (Quercetin, Quercetin-*O*-deoxyhexoside, Quercetin-*O*-(galloyl)hexoside, Quercetin-*O*-(galloyl)deoxyhexoside and Galloylquercetin-*O*-hexoside).

**Species of *Myrica* L.**	**Sub-species ∣ Variety ∣ Location**	**Maturation stage**	**Reference**	**Quercetin** (mg/g)	**Quercetin-*O*-deoxyhexoside**	**Quercetin-*O*-(galloyl)hexoside** (mg/g DE)	**Quercetin-*O*-(galloyl) deoxyhexoside** (mg/g DE)	**Galloylquercetin-*O*-hexoside** (mg/100 g DW)
MR	DongKui	Veraison	(Chen et al., 2020) [2]	0.66				
		Mature	(Chen et al., 2020) [2]	0.89				
	Muye	Veraison	(Chen et al., 2020) [2]	13.89				
		Mature	(Chen et al., 2020) [2]	8.78				

	ZaoJia	Veraison	(Chen et al., 2020) [2]	0.17				
		Mature	(Chen et al., 2020) [2]	0.76				
	–	–	(Xia et al., 2023) [7]	0.00079				
MF	–	–	(Spínola et al., 2014) [5]		33.95 ± 1.35 [a]			41.73 ± 2.06
	Machico	–	(Spínola et al., 2019) [9]			0.47	0.40 ± 0.01	
	Faial	–	(Spínola et al., 2019) [9]				0.38 ± 0.02	
	Arco de São Jorge	–	(Spínola et al., 2019) [9]			0.32	0.49	
	Boaventura	–	(Spínola et al., 2019) [9]		0.24 ± 0.01 [b]	0.25	0.27 ± 0.01	
	Seixal	–	(Spínola et al., 2019) [9]			0.17 ± 0.02		
	Ribeira da Janela	–	(Spínola et al., 2019) [9]			0.15 ± 0.01	1.13 ± 0.01	
	Porto Moniz	–	(Spínola et al., 2019) [9]		0.25 ± 0.01 [b]	0.19 ± 0.01	1.00	
	Terceira	–	(Spínola et al., 2019) [9]		0.27 ± 0.01 [b]	0.39	1.55	

[a] mg/100 g DW | [b] mg/g DE

Table S21. Flavanols (Gallo(epi)catechin, Gallocatechin dimer, Digalloyl(epi)gallocatechin dimer and (+)Catechin).

**Species of *Myrica* L.**	**Sub-species | Variety | Location**	**Reference**	**Gallo(epi)catechin (mg/g DE)**	**Gallocatechin dimer (mg/g DE)**	**Digalloyl(epi)gallocatechin dimer (mg/g DE)**	**(+)Catechin (mg/g DE)**
MF	Machico	(Spínola et al., 2019) [9]	0.20 ± 0.01			0.16 ± 0.01
	Faial	(Spínola et al., 2019) [9]	0.76 ± 0.03		0.50 ± 0.01	

	Arco de São Jorge	(Spínola et al., 2019) [9]	0.12 ± 0.01	0.16 ± 0.01	0.38 ± 0.01	
	Boaventura	(Spínola et al., 2019) [9]	0.11 ± 0.01	0.26 ± 0.01	0.32 ± 0.01	
	Ribeira da Janela	(Spínola et al., 2019) [9]	0.12 ± 0.07		0.37 ± 0.01	0.25 ± 0.01
	Porto Moniz	(Spínola et al., 2019) [9]	0.23 ± 0.01		0.37 ± 0.01	
	Terceira	(Spínola et al., 2019) [9]	0.34 ± 0.01			

Table S22. Flavanols ((epi)catechin), (epi)catechin-*O*-gallate, Gallo(epi)catechin-*O*-gallate and Digallocatechin derivative).

**Species of *Myrica* L.**	**Sub-species | Variety | Location**	**Reference**	**(epi)catechin**	**(epi)catechin-*O*-gallate** (mg/100 g DW)	**Gallo(epi)catechin-*O*-gallate** (mg/g DE)	**Digallocatechin derivative** (mg/g DE)
MF	–	(Spínola et al., 2014) [5]	ND	37.80 ± 1.22		
	Machico	(Spínola et al., 2019) [9]			0.04 ± 0.01	
	Faial	(Spínola et al., 2019) [9]			0.58	0.45 ± 0.01
	Boaventura	(Spínola et al., 2019) [9]			0.05 ± 0.01	
	Seixal	(Spínola et al., 2019) [9]			0.25 ± 0.01	0.14 ± 0.01
	Ribeira da Janela	(Spínola et al., 2019) [9]			0.06 ± 0.01	
	Porto Moniz	(Spínola et al., 2019) [9]			0.05 ± 0.01	
	Terceira	(Spínola et al., 2019) [9]				0.28 ± 0.01

ND = Not detected

Table S23. Flavones (Tricin-*O*-hexoside, Tricin-*O*-hexoside derivative and Luteolin-*O*-hexoside derivative).

Species of *Myrica* L.	Sub-species I Variety I Location	Reference	Tricin-*O*-hexoside (mg/100 g DW)	Tricin-*O*-hexoside derivative (mg/g DE)	Luteolin-*O*-hexoside derivative (mg/100 g DW)
MF	–	(Spínola et al., 2014) [5]	5.01 ± 0.94		77.68 ± 5.44
	Machico	(Spínola et al., 2019) [9]		0.25 ± 0.01	
	Porto Moniz	(Spínola et al., 2019) [9]		0.11 ± 0.01	
	Terceira	(Spínola et al., 2019) [9]		0.21 ± 0.01	

Table S24. Ellagitannins (Pedunculagin I, Casuarinin, Ellagitannin, Hexahydroxydiphenoyl-*O*-hexoside and Ellagic acid-*O*-pentoside).

Species of *Myrica* L.	Sub-species I Variety I Location	Reference	Pedunculagin I (mg/g DE)	Casuarinin (mg/g DE)	Ellagitannin (mg/g DE)	Hexahydroxydiphenoyl-*O*-hexoside (mg/g DE)	Ellagic acid-*O*-pentoside (mg/g DE)
MF	Machico	(Spínola et al., 2019) [9]	0.24	0.44	0.10 ± 0.01	0.05 ± 0.01	
	Faial	(Spínola et al., 2019) [9]	0.19 ± 0.01	0.44 ± 0.01		0.14 ± 0.01	
	Arco de São Jorge	(Spínola et al., 2019) [9]	0.39 ± 0.01		0.26 ± 0.01		0.17 ± 0.01
	Boaventura	(Spínola et al., 2019) [9]	0.63	0.30	0.21 ± 0.01		
	Seixal	(Spínola et al., 2019) [9]	0.61	0.20 ± 0.01		0.25 ± 0.01	0.29 ± 0.01
	Ribeira da Janela	(Spínola et al., 2019) [9]	0.66	0.21 ± 0.01	0.16 ± 0.01	0.06 ± 0.01	

	Porto Moniz	(Spínola et al., 2019) [9]	0.41	0.29	0.12 ± 0.01		0.22 ± 0.02
	Terceira	(Spínola et al., 2019) [9]	0.47 ± 0.01	0.23 ± 0.01	0.22 ± 0.01		0.32 ± 0.02

Table S25. Anthocyanins (Cyanidin-3-*O*-galactoside, Cyanidin-3-*O*-glucoside and Cyanidin-*O*-hexoside).

**Species of *Myrica* L.**	**Sub-species | Variety | Location**	**Reference**	**Cyanidin-3-*O*-galactoside** (µg/g FW)	**Cyanidin-3-*O*-glucoside**	**Cyanidin-*O*-hexoside**
MR	Biqi	(Zhang et al., 2015) [1]	13.71 ± 1.32	837.32 ± 36.95 [a]	
	Ciji	(Zhang et al., 2015) [1]	11.35 ± 0.39	636.49 ± 54.95 [a]	
	Ding'ao	(Zhang et al., 2015) [1]	14.26 ± 0.24	513.02 ± 8.18 [a]	
	Dongkui	(Zhang et al., 2015) [1]	9.50 ± 0.52	287.86 ± 9.68 [a]	
		(Chen et al., 2020) [2]		892.15 [d]	
	Dayexidi	(Zhang et al., 2015) [1]	10.72 ± 0.53	901.43 ± 20.97 [a]	
	Fenhong	(Zhang et al., 2015) [1]	8.75 ± 0.16	74.55 ± 1.59 [a]	
	Muye	(Chen et al., 2020) [2]		2046.04 [d]	
	Muyezhong	(Zhang et al., 2015) [1]	10.34 ± 0.71	582.63 ± 15.32 [a]	
	Shenhong-zhong	(Zhang et al., 2015) [1]	9.70 ± 0.68	143.71 ± 2.42 [a]	
	Shuijing	(Zhang et al., 2015) [1]	ND	9.34 ± 0.14 [a]	
	Shuimei	(Zhang et al., 2015) [1]	9.44 ± 0.82	187.21 ± 5.27 [a]	
	Tanmei	(Zhang et al., 2015) [1]	11.81 ± 0.22	829.20 ± 42.32 [a]	

	Wandao	(Zhang et al., 2015) [1]	14.98 ± 1.80	912.24 ± 84.84 [a]	
	Wumei	(Zhang et al., 2015) [1]	10.70 ± 0.15	294.31 ± 7.35 [a]	
	Wuzi	(Zhang et al., 2015) [1]	35.35 ± 2.92	537.98 ± 8.98 [a]	
	Xiaoyexidi	(Zhang et al., 2015) [1]	9.22 ± 0.41	391.54 ± 6.95 [a]	
	Zaodamei	(Zhang et al., 2015) [1]	23.20 ± 3.31	340.02 ± 20.9 [a]	
	ZaoJia	(Chen et al., 2020) [2]		1618.53 [d]	
	Zaose	(Zhang et al., 2015) [1]	9.90 ± 0.38	223.06 ± 8.71 [a]	
MF	–	(Spínola et al., 2014) [5]			368.57 ± 5.42 [c]
	Machico	(Spínola et al., 2019) [9]		24.47 ± 0.05 [b]	0.28 ± 0.01 [b]
	Faial	(Spínola et al., 2019) [9]		26.54 ± 0.46 [b]	
	Arco de São Jorge	(Spínola et al., 2019) [9]		33.19 ± 0.25 [b]	0.23 ± 0.01 [b]
	Boaventura	(Spínola et al., 2019) [9]		32.01 ± 0.18 [b]	0.15 ± 0.01 [b]
	Seixal	(Spínola et al., 2019) [9]		28.53 ± 0.10 [b]	
	Ribeira da Janela	(Spínola et al., 2019) [9]		23.73 ± 0.30 [b]	
	Porto Moniz	(Spínola et al., 2019) [9]		27.33 ± 0.61 [b]	0.34 ± 0.01 [b]
	Terceira	(Spínola et al., 2019) [9]		27.20 ± 0.05 [b]	

[a] μg/g FW | [b] mg/g DE | [c] mg/100 g DW | [d] μg/g | ND = Not Detected

Table S26. Anthocyanins (Cyanidin, Cyanidin-*O*-pentoside, Cyanidin-*O*-(acetyl)hexoside, Peonidin-3-*O*-glucoside and Peonidin).

Species of *Myrica* L.	Sub-species | Variety | Location	Reference	Cyanidin (ng/g)	Cyanidin-*O*-pentoside (mg/g DE)	Cyanidin-*O*-(acetyl)hexoside (mg/g DE)	Peonidin-3-*O*-glucoside (μg/g FW)	Peonidin (ng/g)
MR	Biqi	(Zhang et al., 2015) [1]				19.09 ± 0.91	
	Ciji	(Zhang et al., 2015) [1]				15.04 ± 1.43	
	Ding'ao	(Zhang et al., 2015) [1]				15.17 ± 0.83	
	Dongkui	(Zhang et al., 2015) [1]				10.94 ± 0.65	
		(Chen et al., 2020) [2]	0.00 – 2.00				0.00 – 2.00
	Dayexidi	(Zhang et al., 2015) [1]				17.17 ± 0.62	
	Fenhong	(Zhang et al., 2015) [1]				9.10 ± 0.16	
	Muye	(Chen et al., 2020) [2]	4.00 – 6.00				0.00 – 2.00
	Muyezhong	(Zhang et al., 2015) [1]				11.68 ± 1.79	
	Shenhong-zhong	(Zhang et al., 2015) [1]				10.70 ± 0.90	
	Shuijing	(Zhang et al., 2015) [1]				ND	
	Shuimei	(Zhang et al., 2015) [1]				9.69 ± 0.83	
	Tanmei	(Zhang et al., 2015) [1]				20.48 ± 1.46	
	Wandao	(Zhang et al., 2015) [1]				10.42 ± 0.78	
	Wumei	(Zhang et al., 2015) [1]				9.95 ± 0.12	
	Wuzi	(Zhang et al., 2015) [1]				13.63 ± 1.34	

	Xiaoyexidi	(Zhang et al., 2015) [1]				10.30 ± 0.89	
	Zaodamei	(Zhang et al., 2015) [1]				10.29 ± 1.31	
	ZaoJia	(Chen et al., 2020) [2]	4.00 – 6.00				0.00 – 2.00
	Zaose	(Zhang et al., 2015) [1]				10.65 ± 0.62	
MF	–	(Spínola et al., 2014) [5]					
	Machico	(Spínola et al., 2019) [9]			0.21 ± 0.01		
	Faial	(Spínola et al., 2019) [9]					
	Arco de São Jorge	(Spínola et al., 2019) [9]		0.17 ± 0.01	0.20 ± 0.01		
	Boaventura	(Spínola et al., 2019) [9]			0.30 ± 0.01		
	Seixal	(Spínola et al., 2019) [9]					
	Ribeira da Janela	(Spínola et al., 2019) [9]					
	Porto Moniz	(Spínola et al., 2019) [9]			0.33 ± 0.01		
	Terceira	(Spínola et al., 2019) [9]			0.21 ± 0.08		

[a] µg/g FW Ι [b] mg/g DE Ι [c] mg/100 g DW Ι ND = Not Detected

Table S27. Anthocyanins (Delphinidin-*O*-hexoside, Delphinidin-hexoside, Delphinidin, Pelargonidin and Pelargonidin-3-*O*-glucoside).

**Species of *Myrica* L.**	**Sub-species Ι Variety Ι Location**	**Reference**	**Delphinidin-*O*-hexoside**	**Delphinidin-hexoside** (µg/g FW)	**Delphinidin** (ng/g)	**Pelargonidin** (ng/g)	**Pelargonidin-3-*O*-glucoside** (µg/g FW)
MR	Biqi	(Zhang et al., 2015) [1]		11.10 ± 0.52			10.38 ± 0.56

	Ciji	(Zhang et al., 2015) [1]		9.86 ± 0.50			9.32 ± 0.36
	Ding'ao	(Zhang et al., 2015) [1]		11.46 ± 0.35			10.05 ± 0.52
	Dongkui	(Zhang et al., 2015) [1]		9.37 ± 0.49			9.30 ± 0.52
		(Chen et al., 2020) [2]			8.00 – 10.00	0.00 – 2.00	
	Dayexidi	(Zhang et al., 2015) [1]		10.41 ± 0.39			9.64 ± 0.27
	Fenhong	(Zhang et al., 2015) [1]		8.69 ± 0.13			8.65 ± 0.12
	Muye	(Chen et al., 2020) [2]			8.00 – 10.00	0.00 – 2.00	
	Muyezhong	(Zhang et al., 2015) [1]		10.22 ± 0.61			9.72 ± 0.59
	Shenhong-zhong	(Zhang et al., 2015) [1]		9.70 ± 0.67			9.56 ± 0.69
	Shuijing	(Zhang et al., 2015) [1]		ND			ND
	Shuimei	(Zhang et al., 2015) [1]		9.05 ± 0.74			9.47 ± 0.65
	Tanmei	(Zhang et al., 2015) [1]		10.67 ± 0.7			9.54 ± 0.52
	Wandao	(Zhang et al., 2015) [1]		9.60 ± 0.34			9.15 ± 0.53
	Wumei	(Zhang et al., 2015) [1]		9.35 ± 0.18			9.81 ± 0.21
	Wuzi	(Zhang et al., 2015) [1]		10.08 ± 0.48			9.78 ± 0.39
	Xiaoyexidi	(Zhang et al., 2015) [1]		8.95 ± 0.45			8.83 ± 0.35
	Zaodamei	(Zhang et al., 2015) [1]		8.67 ± 0.76			8.60 ± 0.69
	ZaoJia	(Chen et al., 2020) [2]			6.00 – 8.00	0.00 – 2.00	
	Zaose	(Zhang et al., 2015) [1]		9.20 ± 0.36			9.18 ± 0.23
MF	–	(Spínola et al., 2014) [5]	16.52 ± 0.36 [a]				
	Machico	(Spínola et al., 2019) [9]	2.28 [b]				

	Faial	(Spínola et al., 2019) [9]	1.97 [b]				
	Arco de São Jorge	(Spínola et al., 2019) [9]	2.34 [b]				
	Boaventura	(Spínola et al., 2019) [9]	2.65 [b]				
	Seixal	(Spínola et al., 2019) [9]	1.68 [b]				
	Ribeira da Janela	(Spínola et al., 2019) [9]	1.91 [b]				
	Porto Moniz	(Spínola et al., 2019) [9]	1.75 [b]				
	Terceira	(Spínola et al., 2019) [9]	1.91 [b]				

[a] mg/100 g DW | [b] mg/g DE | ND = Not Detected

Table S28. Chlorophyll and carotenoids (*α*- Carotenoid, β- Carotenoid and Lycopene).

**Species of *Myrica* L.**	**Sub-species | Variety**	**Maturation stage**	**Reference**	**Chlorophyll** (mg/g)	***α*- Carotenoid**	**β- Carotenoid** (μg/g)	**Lycopene**
MR	DongKui	–	(Chen et al., 2020) [2]		ND	0.0954	ND
		Veraison	(Chen et al., 2020) [2]	6.00 – 8.00			
		Mature	(Chen et al., 2020) [2]	4.00 – 6.00			
	Muye	–	(Chen et al., 2020) [2]		ND	0.3423	ND
		Veraison	(Chen et al., 2020) [2]	8.00 – 10.00			
		Mature	(Chen et al., 2020) [2]	4.00 – 6.00			
	ZaoJia	–	(Chen et al., 2020) [2]		ND	ND	ND
		Veraison	(Chen et al., 2020) [2]	2.00 – 4.00			

		Mature	(Chen et al., 2020) [2]	8.00 – 10.00 [a]			

ND = Not Detected

Table S29. Identification of components.

Compound	Retention time (min)	Relative content (%)	Molecular weight	(*m*/*z*)	MR	MF	Ref.
(epi)catechin	6.4			289 (-)		x	(Spínola et al., 2014) [5]
(epi)catechin-*O*-gallate	10.2			441 (-)		x	(Spínola et al., 2014) [5]
5,7-dihydroxy-6,8-dimethoxyflavone-7-*O*-glucuronide	30.4			535 (-)		x	(Spínola et al., 2014) [5]
Benzoyl-*p*-dicoumaryl-2,7-anhydro-3-deoxy-2-octulopyranosonic acid	15.3			633 (-)		x	(Spínola et al., 2014) [5]
Benzoyl-*p*-tricoumaryl-2,7-anhydro-3-deoxy-2-octulopyranosonic acid	31.9			779 (-)		x	(Spínola et al., 2014) [5]
Caffeic acid-*O*-hexoside	3.2			341 (-)		x	(Spínola et al., 2014) [5]
Conidendrin-*O*-hexoside	29.4			563 (-)		x	(Spínola et al., 2014) [5]
Cyanidin-3-glucoside	3.0			449 (+)		x	(Spínola et al., 2014) [5]
Cyanidin-3–*O*-galactoside	12.96		449	449.0 (+)<br>287.1 (+)	x		(Zhang et al., 2015) [1]
Cyanidin-3-*O*-glucoside	5.92	95.6		449 (+)	x		(Yun et al., 2019) [10]
	14.91		449	449.8 (+)<br>287.1 (+)	x		(Zhang et al., 2015) [1]

				288.1 (+)			
Delphinidin–3– hexoside	9.86		465	465.2 (+)<br>303.1 (+)	x		(Zhang et al., 2015) [1]
Delphinidin-3-*O*-glucose	1.85	3.7		465 (+)	x		(Yun et al., 2019) [10]
Delphinidin-*O*-hexoside	4.3			465 (+)		x	(Spínola et al., 2014) [5]
Gallo(epi)catechin-*O*-gallate	6.8			457 (-)		x	(Spínola et al., 2014) [5]
Galloyl-*O*-hexoside	3.4			331 (-)		x	(Spínola et al., 2014) [5]
Galloylquercetin-*O*-hexoside	10.3			615 (-)		x	(Spínola et al., 2014) [5]
Glucaric acid derivative	8.4			597 (-)		x	(Spínola et al., 2014) [5]
Kaempferol-3–*O*-galactoside	47.55		448	447.2 (-)<br>285.0 (-)<br>284.2 (-)	x		(Zhang et al., 2015) [1]
Kaempferol-3–*O*-glucoside	49.88		448	447.2 (-)<br>285.2 (-)<br>284.1 (-)	x		(Zhang et al., 2015) [1]
Kaempferol-*O*-hexoside	12.1			447 (-)		x	(Spínola et al., 2014) [5]
Kaempferol-*O*-rhamnoside	17.6			431 (-)		x	(Spínola et al., 2014) [5]
Luteolin-*O*-hexoside derivative	4.8			483 (-)		x	(Spínola et al., 2014) [5]
Myricetin deoxyhexoside–gallate	58.16		616	615.1 (-)<br>463.1 (-)<br>317.0 (-)	x		(Zhang et al., 2015) [1]
Myricetin-3–*O*-rhamnoside	42.17		464	463.1 (-)<br>316.0 (-)<br>317.0 (-)	x		(Zhang et al., 2015) [1]

Myricetin-*O*-(*O*-galloyl)deoxyhexoside	16.3			615 (-)		x	(Spínola et al., 2014) [5]
Myricetin-*O*-(*O*-galloyl)deoxyhexoside	17.3			615 (-)		x	(Spínola et al., 2014) [5]
Myricetin-*O*-(*O*-galloyl)hexoside	7.7			631 (-)		x	(Spínola et al., 2014) [5]
Myricetin-*O*-hexoside	7.5			479 (-)		x	(Spínola et al., 2014) [5]
Oxo-dihydroxy-octadecenoic acid	34.7			327 (-)		x	(Spínola et al., 2014) [5]
Pelargonidin-3-*O*-glucose	7.56	0.7		433 (+)	x		(Yun et al., 2019) [10]
Pelargonidin-3–*O*-glucoside	17.87		433	433.1 (+)<br>271.1 (+)<br>272.1 (+)	x		(Zhang et al., 2015) [1]
Peonidin-3–*O*-glucoside	19.58		463	463.0 (+)<br>300.1 (+)<br>301.1 (+)	x		(Zhang et al., 2015) [1]
Quercetin-3– *O*-glucoside	43.64		464	463.3 (-)<br>301.2 (-)<br>300.1 (-)	x		(Zhang et al., 2015) [1]
Quercetin-3–*O*-galactoside	42.94		464	463.3 (-)<br>301.1 (-)<br>300.1 (-)	x		(Zhang et al., 2015) [1]
Quercetin-3–*O*-rhamnoside	50.55		448	447.2 (-)<br>301.2 (-)<br>300.3 (-)	x		(Zhang et al., 2015) [1]
Quercetin-*O*-deoxyhexoside	13.4			447 (-)		x	(Spínola et al., 2014) [5]

Quinic acid	3.3			191 (-)		x	(Spínola et al., 2014) [5]
Quinic acid derivative	4.5			405 (-)		x	(Spínola et al., 2014) [5]
Roseoside	5.4			431 (-)		x	(Spínola et al., 2014) [5]
Tricin-*O*-hexoside	26.6			491 (-)		x	(Spínola et al., 2014) [5]
Tricin-*O*-hexoside	29.1			491 (-)		x	(Spínola et al., 2014) [5]

Leaves' phytochemical, physicochemical and nutritional composition

Table S30. Nutritional composition.

Species of *Myrica* L.	Sub-species ǀ Variety	Extraction ǀ Method	Ref.	Carbohydrates	Proteins & amino acids	Fixed oils & fats
ME	–	Aqueous	(Kabra et al., 2019b) [11]	Present	Absent	Absent
	–	Ethyl acetate	(Kabra et al., 2019b) [11]	Present	Absent	Absent
	–	Methanolic	(Kabra et al., 2019b) [11]	Present	Absent	Absent

Table S31. Total phenolics and total flavonoid content determined by spectrophotometry.

Species of *Myrica* L.	Sub-species ǀ Variety	Extraction ǀ Method	Ref.	Total phenolics	Total flavonoids
MR	–	–	(Zhang et al., 2016) [12]		920.78 ± 18.88 [b]
	–	*in vitro* digestion	(Zhang et al., 2016) [12]		669.18 ± 40.95 [b]
	–	–	(Zhang et al., 2018) [13]	378.28 ± 0.97 [a]	
ME	–	Aqueous	(Kabra et al., 2019b) [11]	Present	Present
	–		(Kabra et al., 2019a) [14]	62.38 ± 0.14 [d]	35.77 ± 0.14 [c]
	–	Ethyl acetate	(Kabra et al., 2019b) [11]	Present	Present
	–		(Kabra et al., 2019a) [14]	75.83 ± 0.19 [d]	46.83 ± 0.19 [c]
	–	Methanolic	(Kabra et al., 2019b) [11]	Present	Present
	–		(Kabra et al., 2019a) [14]	88.94 ± 0.24 [d]	67.44 ± 0.14 [c]

MF	–	–	(Spínola et al., 2014) [5]	248.00 [e]	127.20 [f]
	Machico	–	(Spínola et al., 2019) [9]	212.43 ± 3.68 [e]	57.91 ± 1.01 [f]
	Faial	–	(Spínola et al., 2019) [9]	226.49 ± 6.99 [e]	61.57 ± 2.04 [f]
	Arco de São Jorge	–	(Spínola et al., 2019) [9]	248.76 ± 5.18 [e]	63.89 ± 1.82 [f]
	Boaventura	–	(Spínola et al., 2019) [9]	251.5 ± 5.71 [e]	67.38 ± 1.50 [f]
	Seixal	–	(Spínola et al., 2019) [9]	232.64 ± 3.33 [e]	65.35 ± 1.39 [f]
	Ribeira da Janela	–	(Spínola et al., 2019) [9]	219.29 ± 3.97 [e]	54.89 ± 1.52 [f]
	Porto Moniz	–	(Spínola et al., 2019) [9]	239.25 ± 4.62 [e]	55.31 ± 2.57 [f]
	Terceira	–	(Spínola et al., 2019) [9]	257.76 ± 3.09 [e]	71.41 ± 1.70 [f]

[a] mg GAE/g DW | [b] mg RE/g DW | [c] mg/g QE | [d] mg/g GAE | [e] mg GAE/g DE | [f] mg RE/g DE

Table S32. Total individual phenolic, total phenolic acids, toral flavanols, total flavonols and total flavones content determined by HPLC.

**Species of *Myrica* L.**	**Sub-species | Variety | Location**	**Reference**	**Total individual phenolic**	**Total phenolic acids**	**Total flavanols**	**Total flavonols**	**Total flavones**
MF	–	(Spínola et al., 2014) [5]	1540.38 ± 87.76 [a]	7.53 ± 0.9 [a]	178.04 ± 4.33 [a]	1241.97 ± 10.79 [a]	26.38 ± 1.48 [a]
	Machico	(Spínola et al., 2019) [9]	87.48 ± 1.26 [b]		26.21 ± 0.67 [b]	32.78 ± 0.5 [b]	0.41 ± 0.01 [b]
	Faial	(Spínola et al., 2019) [9]	91.87 ± 1.46 [b]		26.50 ± 0.70 [b]	36.48 ± 1.53 [b]	1.06 ± 0.01 [b]
	Arco de São Jorge	(Spínola et al., 2019) [9]	93.15 ± 2.16 [b]		21.99 ± 0.61 [b]	41.99 ± 0.86 [b]	
	Boaventura	(Spínola et al., 2019) [9]	102.35 ± 2.00 [b]		28.08 ± 0.59 [b]	46.04 ± 1.13 [b]	0.87 ± 0.01 [b]

	Seixal	(Spínola et al., 2019) [9]	96.70 ± 1.25 [b]		24.18 ± 0.52 [b]	43.50 ± 0.36 [b]	0.43 ± 0.01 [b]
	Ribeira da Janela	(Spínola et al., 2019) [9]	83.45 ± 1.91 [b]		26.35 ± 0.39 [b]	33.02 ± 0.52 [b]	
	Porto Moniz	(Spínola et al., 2019) [9]	96.61 ± 2.08 [b]		28.04 ± 0.96 [b]	35.77 ± 0.64 [b]	
	Terceira	(Spínola et al., 2019) [9]	100.60 ± 1.49 [b]		30.78 ± 0.50 [b]	26.79 ± 0.63 [b]	0.64 ± 0.01 [b]

[a] mg/100 g DW | [b] mg/g DE

Table S33. Total hydroxycinnamic acids and total hydroxybenzoic acids and total ellagitannins content determined by HPLC.

**Species of *Myrica* L.**	**Sub-species | Variety | Location**	**Reference**	**Total hydroxycinnamic acids (mg/g DE)**	**Total hydroxybenzoic acids (mg/g DE)**	**Total ellagitannins**
MF	–	(Spínola et al., 2014) [5]			86.46 ± 6.75 [a]
	Machico	(Spínola et al., 2019) [9]	0.07 ± 0.01	7.10 ± 0.29	20.91 ± 0.27 [b]
	Faial	(Spínola et al., 2019) [9]	0.03 ± 0.01	6.13 ± 0.35	21.67 ± 0.86 [b]
	Arco de São Jorge	(Spínola et al., 2019) [9]	0.05 ± 0.01	6.69 ± 0.25	22.43 ± 0.28 [b]
	Boaventura	(Spínola et al., 2019) [9]	0.04 ± 0.01	4.49 ± 0.09	22.83 ± 1.05 [b]
	Seixal	(Spínola et al., 2019) [9]		5.38 ± 0.04	23.21 ± 0.59 [b]
	Ribeira da Janela	(Spínola et al., 2019) [9]	0.08 ± 0.01	5.07 ± 0.36	18.96 ± 0.64 [b]
	Porto Moniz	(Spínola et al., 2019) [9]	0.04 ± 0.01	5.51 ± 0.23	27.25 ± 0.79 [b]
	Terceira	(Spínola et al., 2019) [9]	0.24 ± 0.01	8.36 ± 0.50	33.79 ± 1.12 [b]

[a] mg/100 g DW | [b] mg/g DE

Table S34. Hydroxycinnamic acids (Coumaroyl-*O*-hexoside and Benzoyl-*p*-tri-coumaroyl-2,7-anhydro-3-deoxy-2-octulopyranosonic acid).

**Species of *Myrica* L.**	**Sub-species ǀ Variety ǀ Location**	**Reference**	**Coumaroyl-*O*-hexoside** (mg/g DE)	**Benzoyl-*p*-tri-coumaroyl-2,7-anhydro-3-deoxy-2-octulopyranosonic acid** (mg/g DE)
MF	Machico	(Spínola et al., 2019) [9]		0.07 ± 0.01
	Faial	(Spínola et al., 2019) [9]		0.03 ± 0.01
	Arco de São Jorge	(Spínola et al., 2019) [9]		0.05 ± 0.01
	Boaventura	(Spínola et al., 2019) [9]		0.04 ± 0.01
	Ribeira da Janela	(Spínola et al., 2019) [9]		0.08 ± 0.01
	Porto Moniz	(Spínola et al., 2019) [9]		0.04 ± 0.01
	Terceira	(Spínola et al., 2019) [9]	0.21 ± 0.01	0.03 ± 0.01

Table S35. Hydroxybenzoic acids (Galloyl-*O*-hexoside, Galloylquinic acid, Gallic acid, Trigalloyl-*O*-hexoside, Tetragalloyl-*O*-hexoside and Protocatechuic acid-*O*-pentoside).

**Species of *Myrica* L.**	**Sub-species ǀ Variety ǀ Location**	**Reference**	**Galloyl-*O*-hexoside** (mg/g DE)	**Galloylquinic acid** (mg/g DE)	**Gallic acid** (mg/g DE)	**Trigalloyl-*O*-hexoside** (mg/g DE)	**Tetragalloyl-*O*-hexoside** (mg/g DE)	**Protocatechuic acid-*O*-pentoside** (mg/100 g DW)
MF	–	(Spínola et al., 2014) [5]						7.53 ± 0.9
	Machico	(Spínola et al., 2019) [9]	3.13 ± 0.06	2.08 ± 0.09	1.67 ± 0.03	0.22 ± 0.01		
	Faial	(Spínola et al., 2019) [9]	3.25 ± 0.10	2.65 ± 0.06		0.23 ± 0.01		

	Arco de São Jorge	(Spínola et al., 2019) [9]	3.58 ± 0.04	2.39 ± 0.05		0.25 ± 0.01	0.47 ± 0.02	
	Boaventura	(Spínola et al., 2019) [9]	2.12 ± 0.03	1.42 ± 0.01	0.79 ± 0.02	0.16 ± 0.01		
	Seixal	(Spínola et al., 2019) [9]	2.76 ± 0.01	1.61 ± 0.01	0.83 ± 0.01	0.18 ± 0.01		
	Ribeira da Janela	(Spínola et al., 2019) [9]	1.99 ± 0.08	2.02 ± 0.04	0.94 ± 0.18	0.12 ± 0.01		
	Porto Moniz	(Spínola et al., 2019) [9]	2.97 ± 0.05	1.76 ± 0.07	0.56 ± 0.02	0.10 ± 0.01	0.12 ± 0.01	
	Terceira	(Spínola et al., 2019) [9]	3.45 ± 0.29	3.19 ± 0.02		0.49 ± 0.03	1.13 ± 0.03	

Table S36. Flavonols (Myricitrin, Myricetin-*O*-hexoside, Myricetin-*O*-(*O*-galloyl)hexoside, Myricetin-*O*-deoxyhexoside and Dimethyl-myricetin-*O*-pentoside).

**Species of *Myrica* L.**	**Sub-species ǀ Variety ǀ Location**	**Extraction ǀ Method**	**Reference**	**Myricitrin**	**Myricetin-*O*-hexoside**	**Myricetin-*O*-(*O*-galloyl)hexoside (mg/100 g DW)**	**Myricetin-*O*-deoxyhexoside (mg/100 g DW)**	**Dimethyl-myricetin-*O*-pentoside (mg/g DE)**
MR	–	–	(Zhang et al., 2016) [12]	184.36 ± 5.96 [a]				
	–	Digested	(Zhang et al., 2016) [12]	36.29 ± 0.35 [a]				
MF	–	–	(Spínola et al., 2014) [5]		62.79 ± 2.15 [c]	53.59 ± 1.59	770.35 ± 11.31	
	Machico	–	(Spínola et al., 2019) [9]	15.91 ± 0.03 [b]	3.11 ± 0.09 [b]			0.32 ± 0.01
	Faial	–	(Spínola et al., 2019) [9]	21.57 ± 0.53 [b]	2.43 ± 0.18 [b]			
	Arco de São Jorge	–	(Spínola et al., 2019) [9]	19.59 ± 0.09 [b]	3.58 ± 0.16 [b]			
	Boaventura	–	(Spínola et al., 2019) [9]	18.47 ± 0.38 [b]	5.39 ± 0.17 [b]			0.19 ± 0.01

	Seixal	–	(Spínola et al., 2019) [9]	23.55 ± 0.11 [b]	3.10 ± 0.01 [b]			
	Ribeira da Janela	–	(Spínola et al., 2019) [9]	17.59 ± 0.09 [b]	1.75 ± 0.06 [b]			
	Porto Moniz	–	(Spínola et al., 2019) [9]	21.66 ± 0.28 [b]	1.17 ± 0.01 [b]			0.25 ± 0.01
	Terceira	–	(Spínola et al., 2019) [9]	13.27 ± 0.31 [b]	1.22 ± 0.04 [b]			

[a] mg myricitrin equivalent (MYE)/g DW | [b] mg/g DE | [c] mg/100 g DW

Table S37. Flavonols (Myricetin-*O*-(*O*-galloyl)deoxyhexoside, Myricetin-*O*-(galloyl)hexoside, Myricetin-*O*-(galloyl)deoxyhexoside and Myricetin-*O*-pentoside).

**Species of *Myrica* L.**	**Sub-species ǀ Variety ǀ Location**	**Reference**	**Myricetin-*O*-(*O*-galloyl)deoxyhexoside** (mg/100 g DW)	**Myricetin-*O*-(galloyl)hexoside** (mg/g DE)	**Myricetin-*O*-(galloyl)deoxyhexoside** (mg/g DE)	**Myricetin-*O*-pentoside** (mg/g DE)
MF	–	(Spínola et al., 2014) [5]	233.98 ± 6.55			
	Machico	(Spínola et al., 2019) [9]		3.90	3.00 ± 0.08	
	Faial	(Spínola et al., 2019) [9]		2.84 ± 0.06	2.89 ± 0.13	
	Arco de São Jorge	(Spínola et al., 2019) [9]		7.47	2.32 ± 0.07	
	Boaventura	(Spínola et al., 2019) [9]		9.95	1.17 ± 0.02	
	Seixal	(Spínola et al., 2019) [9]		6.09 ± 0.08	3.73 ± 0.04	
	Ribeira da Janela	(Spínola et al., 2019) [9]		3.80	2.92 ± 0.03	
	Porto Moniz	(Spínola et al., 2019) [9]		2.66	2.26 ± 0.06	0.57 ± 0.01

	Terceira	(Spínola et al., 2019) [9]		4.19 ± 0.03	3.24 ± 0.11	

Table S38. Flavonols (Kaempferol-*O*-rutinoside, Kaempferol-*O*-hexoside, Kaempferol-*O*-(*O*-galloyl)hexoside, Kaempferol-*O*-rhamnoside, Kaempferol-*O*-(galloyl)hexoside and Kaempferol-*O*-deoxyhexoside).

**Species of *Myrica* L.**	**Sub-species ǀ Variety ǀ Location**	**Reference**	**Kaempferol-*O*-rutinoside (mg/100 g DW)**	**Kaempferol-*O*-hexoside**	**Kaempferol-*O*-(*O*-galloyl)hexoside (mg/100 g DW)**	**Kaempferol-*O*-rhamnoside (mg/100 g DW)**	**Kaempferol-*O*-(galloyl)hexoside (mg/g DE)**	**Kaempferol-*O*-deoxyhexoside (mg/g DE)**
MF	–	(Spínola et al., 2014) [5]	13.25 ± 0.85	16.65 ± 1.94 [a]	14.35 ± 0.79	26.27 ± 2.69		
	Machico	(Spínola et al., 2019) [9]		0.68 ± 0.03 [b]			0.37 ± 0.02	0.76 ± 0.01
	Faial	(Spínola et al., 2019) [9]		0.62 ± 0.01 [b]			0.51 ± 0.01	0.58 ± 0.01
	Arco de São Jorge	(Spínola et al., 2019) [9]		0.86 ± 0.02 [b]			0.83 ± 0.01	0.90 ± 0.03
	Boaventura	(Spínola et al., 2019) [9]		0.77 ± 0.03 [b]			0.43 ± 0.01	1.24 ± 0.04
	Seixal	(Spínola et al., 2019) [9]		0.71 ± 0.02 [b]			0.55 ± 0.01	0.93
	Ribeira da Janela	(Spínola et al., 2019) [9]		0.77 ± 0.01 [b]			0.33 ± 0.01	0.76 ± 0.03
	Porto Moniz	(Spínola et al., 2019) [9]		0.60 ± 0.02 [b]			0.55 ± 0.01	1.11 ± 0.04
	Terceira	(Spínola et al., 2019) [9]		0.44 ± 0.01 [b]			0.63 ± 0.01	1.17 ± 0.02

[a] mg/100 g DW ǀ [b] mg/g DE

Table S39. Flavonols (Quercetin 3-rhamnoside, Quercetin-*O*-deoxyhexoside, Quercetin-*O*-rutinoside and Galloylquercetin-*O*-hexoside).

Species of *Myrica* L.	Sub-species ǀ Variety ǀ Location	Extraction ǀ Method	Reference	Quercetin 3-rhamnoside (µg Q3E/g DW)	Quercetin-*O*-deoxyhexoside	Quercetin-*O*-rutinoside (mg/g DE)	Galloylquercetin-*O*-hexoside (mg/100 g DW)
MR	–	–	(Zhang et al., 2016) [12]	127.05 ± 0.42			
	–	Digested	(Zhang et al., 2016) [12]	55.79 ± 0.08			
MF	–		(Spínola et al., 2014) [5]		16.86 ± 0.86 [a]		33.88 ± 2.11
	Machico		(Spínola et al., 2019) [9]		0.60 ± 0.03 [b]	0.64 ± 0.02	
	Faial		(Spínola et al., 2019) [9]		0.79 ± 0.03 [b]	0.57 ± 0.01	
	Arco de São Jorge		(Spínola et al., 2019) [9]		1.15 ± 0.01 [b]		
	Boaventura		(Spínola et al., 2019) [9]		1.42 ± 0.03 [b]	0.23 ± 0.01	
	Seixal		(Spínola et al., 2019) [9]		0.56 ± 0.01 [b]	0.34 ± 0.01	
	Ribeira da Janela		(Spínola et al., 2019) [9]		0.91 ± 0.03 [b]	0.78 ± 0.02	
	Porto Moniz		(Spínola et al., 2019) [9]		0.88 ± 0.03 [b]	0.65 ± 0.01	
	Terceira		(Spínola et al., 2019) [9]		0.57 ± 0.02 [b]		

[a] mg/100 g DW ǀ [b] mg/g DE

Table S40. Flavonols (Quercetin-*O*-hexoside, Quercetin-*O*-(galloyl)hexoside, Quercetin-*O*-(galloyl)deoxyhexoside, Quercetin -*O*-(acetyl)deoxyhexoside and Quercetin).

Species of *Myrica* L.	Sub-species ǀ Variety ǀ Location	Reference	Quercetin-*O*-hexoside (mg/g DE)	Quercetin-*O*-(galloyl)hexoside (mg/g DE)	Quercetin-*O*-(galloyl)deoxyhexoside (mg/g DE)	Quercetin-*O*-(acetyl)deoxyhexoside (mg/g DE)	Quercetin (mg/g DE)
MF	Machico	(Spínola et al., 2019) [9]	0.20 ± 0.01	2.00 ± 0.06	1.29		
	Faial	(Spínola et al., 2019) [9]	0.28 ± 0.01	1.97 ± 0.07	1.43		
	Arco de São Jorge	(Spínola et al., 2019) [9]	0.12 ± 0.01	2.88 ± 0.05	2.06	0.23 ± 0.01	
	Boaventura	(Spínola et al., 2019) [9]	0.33 ± 0.01	2.66 ± 0.03	3.61 ± 0.12	0.17 ± 0.01	
	Seixal	(Spínola et al., 2019) [9]	0.19 ± 0.01	3.75 ± 0.01	0.33 ± 0.01		
	Ribeira da Janela	(Spínola et al., 2019) [9]	0.27 ± 0.01	1.83 ± 0.07	1.31		
	Porto Moniz	(Spínola et al., 2019) [9]	0.35 ± 0.01	1.52 ± 0.03	1.54		
	Terceira	(Spínola et al., 2019) [9]	0.70 ± 0.01	0.57 ± 0.02	0.13 ± 0.01	0.36 ± 0.01	0.16 ± 0.01

[a] mg/100 g DW ǀ [b] mg/g DE

Table S41. Flavanols (Galloyl-di(epi)-gallocatechin, (epi)catechin, Gallo(epi)catechin-*O*-gallate dimer and Gallocatechin).

Species of *Myrica* L.	Sub-species ǀ Variety ǀ Location	Reference	Galloyl-di(epi)-gallocatechin (mg/100 g DW)	(epi)catechin (mg/100 g DW)	Gallo(epi)catechin-*O*-gallate dimer (mg/100 g DW)	Gallocatechin (mg/g DE)
MF	–	(Spínola et al., 2014) [5]	43.42 ± 0.98	2.56 ± 0.41	132.06 ± 8.27	

	Machico	(Spínola et al., 2019) [9]				2.64
	Faial	(Spínola et al., 2019) [9]				0.26 ± 0.03
	Arco de São Jorge	(Spínola et al., 2019) [9]				2.56
	Seixal	(Spínola et al., 2019) [9]				1.64
	Ribeira da Janela	(Spínola et al., 2019) [9]				0.26 ± 0.03
	Porto Moniz	(Spínola et al., 2019) [9]				0.61 ± 0.01
	Terceira	(Spínola et al., 2019) [9]				0.31 ± 0.02

Table S42. Flavanols (Gallo(epi)catechin-Gallo(epi)catechin-*O*-gallate, Digalloyl(epi)gallocatechin dimer, Gallo(epi)catechin-*O*-gallate and Digallo(epi)catechin derivative).

**Species of *Myrica* L.**	**Sub-species ǀ Variety ǀ Location**	**Reference**	**Gallo(epi)catechin-Gallo(epi)catechin-*O*-gallate (mg/g DE)**	**Digalloyl(epi)gallocatechin dimer (mg/g DE)**	**Gallo(epi)catechin-*O*-gallate (mg/g DE)**	**Digallo(epi)catechin derivative (mg/g DE)**
MF	Machico	(Spínola et al., 2019) [9]	2.52 ± 0.01	9.61	9.41	2.03 ± 0.05
	Faial	(Spínola et al., 2019) [9]	3.63 ± 0.03	7.79	8.56	2.96 ± 0.12
	Arco de São Jorge	(Spínola et al., 2019) [9]	6.44 ± 0.04	6.55	6.54 ± 0.12	2.04 ± 0.09
	Boaventura	(Spínola et al., 2019) [9]	5.84 ± 0.03	10.63 ± 0.40	10.63	0.70 ± 0.02
	Seixal	(Spínola et al., 2019) [9]	5.68 ± 0.10	7.60 ± 0.07	7.77	2.77 ± 0.02
	Ribeira da Janela	(Spínola et al., 2019) [9]	4.69 ± 0.03	11.62	8.36	1.48 ± 0.11

	Porto Moniz	(Spínola et al., 2019) [9]	4.92 ± 0.12	13.70	5.82	2.99 ± 0.11
	Terceira	(Spínola et al., 2019) [9]	10.24 ± 0.28	15.82 ± 0.22	4.41 ± 0.12	

Table S43. Flavones (Tricin-*O*-hexoside and Tricin-*O*-hexoside derivative).

Species of *Myrica* L.	Sub-species ǀ Variety ǀ Location	Reference	**Tricin-*O*-hexoside** (mg/100 g DW)	**Tricin-*O*-hexoside derivative** (mg/g DE)
MF	–	(Spínola et al., 2014) [5]	26.38 ± 1.48	
	Machico	(Spínola et al., 2019) [9]		0.41 ± 0.01
	Faial	(Spínola et al., 2019) [9]		0.41 ± 0.01
	Boaventura	(Spínola et al., 2019) [9]		0.87 ± 0.01
	Seixal	(Spínola et al., 2019) [9]		0.43 ± 0.01
	Terceira	(Spínola et al., 2019) [9]		0.64 ± 0.01

Table S44. Ellagitannins (Galloyl-bis-hexahydroxydiphenoyl-*O*-hexoside, Hexahydroxydiphenoyl-*O*-hexoside, Casuarinin and Ellagic acid-*O*-pentoside).

Species of *Myrica* L.	Sub-species ǀ Variety ǀ Location	Reference	**Galloyl-bis-hexahydroxydiphenoyl -*O*-hexoside** (mg/100 g DW)	**Hexahydroxydiphenoyl-*O*-hexoside** (mg/g DE)	**Casuarinin** (mg/g DE)	**Ellagic acid-*O*-pentoside** (mg/g DE)
MF	–	(Spínola et al., 2014) [5]	86.46 ± 6.75			
	Machico	(Spínola et al., 2019) [9]		0.95 ± 0.02	8.78	
	Faial	(Spínola et al., 2019) [9]		0.93	9.07	

	Arco de São Jorge	(Spínola et al., 2019) [9]		1.60	7.04	0.89 ± 0.01
	Boaventura	(Spínola et al., 2019) [9]		1.86	8.67	
	Seixal	(Spínola et al., 2019) [9]		1.18 ± 0.06	7.63	
	Ribeira da Janela	(Spínola et al., 2019) [9]		0.83 ± 0.03	6.40	
	Porto Moniz	(Spínola et al., 2019) [9]		1.72	8.34	1.15 ± 0.01
	Terceira	(Spínola et al., 2019) [9]		2.59 ± 0.05	12.89	1.31 ± 0.07

Table S45. Ellagitannins (Pedunculagin I derivative, Pedunculagin I, Pedunculagin II, Ellagic acid derivative and Ellagitannin).

**Species of *Myrica* L.**	**Sub-species ǀ Variety ǀ Location**	**Reference**	**Pedunculagin I derivative (mg/g DE)**	**Pedunculagin I (mg/g DE)**	**Pedunculagin II (mg/g DE)**	**Ellagic acid derivative (mg/g DE)**	**Ellagitannin (mg/g DE)**
	Machico	(Spínola et al., 2019) [9]	3.62 ± 0.01	3.23 ± 0.13		2.89 ± 0.01	1.44 ± 0.01
	Faial	(Spínola et al., 2019) [9]		6.82		3.74 ± 0.13	1.11 ± 0.05
	Arco de São Jorge	(Spínola et al., 2019) [9]		7.81		2.63 ± 0.02	2.46 ± 0.01
	Boaventura	(Spínola et al., 2019) [9]		6.85		2.61 ± 0.14	2.84 ± 0.12
	Seixal	(Spínola et al., 2019) [9]		9.3		3.91 ± 0.11	1.19 ± 0.05
	Ribeira da Janela	(Spínola et al., 2019) [9]		7.12		2.58 ± 0.05	2.03 ± 0.02
	Porto Moniz	(Spínola et al., 2019) [9]	0.31 ± 0.01	10.54		4.52 ± 0.11	1.31 ± 0.04

	Terceira	(Spínola et al., 2019) [9]		12.38	0.31 ± 0.01	2.50 ± 0.01	1.17 ± 0.06

Table S46. Identification of components

Compound	Retention time (min)	Molecular weight	Measured mass (*m/z*)	MR	ME	MF	Reference
(2R)-3',4''-epoxy-2-hydroxy-1-(4-hydroxyphenyl)-7-(3-methoxyphenyl)heptan-3-one	30.58		343.15323 (+)	x			(Chen et al., 2017) [15]
(Epi)gallocatechin	9.08		305.06546 (-)<br>307.08047 (+)	x			(Chen et al., 2017) [15]
	13.08		305.06549 (-)<br>307.08041 (+)	x			(Chen et al., 2017) [15]
(Epi)gallocatechin gallate	18.40		457.07620 (-)	x			(Chen et al., 2017) [15]
	16.26		457.07562 (-)	x			(Chen et al., 2017) [15]
(Epi)gallocatechin-(epi)gallo-catechin	5.87		611.13873 (+)	x			(Chen et al., 2017) [15]
	8.52		609.12268 (-)<br>611.13922 (+)	x			(Chen et al., 2017) [15]
	9.81		611.13867 (+)	x			(Chen et al., 2017) [15]
	10.16		609.12299 (-)<br>611.13892 (+)	x			(Chen et al., 2017) [15]
(Epi)gallocatechin-(epi)gallo-catechin-(epi)gallocatechin	10.80		913.18127 (-)<br>915.19763 (+)	x			(Chen et al., 2017) [15]
(Epi)gallocatechin-(epi)gallo-catechingallate	10.20		761.13318 (-)	x			(Chen et al., 2017) [15]
	12.32		761.13318 (-)	x			(Chen et al., 2017) [15]

			763.1496 (+)				
(epi)gallocatechin+<br>(epi)gallocatechin-3-*O*-gallate	114.6	1675	1674.0 (-)<br>1521.6 (-)<br>1369.5 (-)	x			(Fu et al., 2014) [16]
(epi)gallocatechin+(epi)catechin-3-<br>*O*-gallate	31.1	744	1510.05 (-)<br>743.15 (-)<br>767.10 (+)<br>745.15 (+)<br>481.30 (+)<br>407.25 (+)	x			(Fu et al., 2014) [16]
(epi)gallocatechin+(epi)gallocatechi<br>n-3-*O*-gallate	26.9	762	761.3 (-)<br>591.3 (-)	x			(Fu et al., 2014) [16]
(epi)gallocatechin-3-*O*-gallate	15.2	458	915.15 (-)<br>571.10 (-)<br>479.10 (-)<br>457.05 (-)<br>939.20 (+)<br>707.35 (+)<br>561.10 (+)<br>481.05 (+)<br>459.10 (+)	x			(Fu et al., 2014) [16]
(Epi)gallocatechingallate-(epi)<br>gallocatechin gallate	14.65		913.14435 (-)<br>915.16028 (+)	x			(Chen et al., 2017) [15]
2 (epi)gallocatechin+<br>(epi)gallocatechin-3-*O*-gallate	45.6	1066	1163.3 (-)<br>1065.6 (-)	x			(Fu et al., 2014) [16]

			423.4 (-)				
	60.1	1218	1315.3 (-)<br>1217.7 (-)<br>423.3 (-)	x			(Fu et al., 2014) [16]
	106.9	1523	1522.0 (-)<br>1369.3 (-)	x			(Fu et al., 2014) [16]
2(epi)catechin-3-*O*-gallate, 2 (epi) catechin+(epi)gallocatechin	34.4	882	765.10 (-)<br>631.05 (-)<br>382.05 (-)<br>883.15 (+)<br>789.15 (+)<br>767.20 (+)<br>719.30 (+)<br>655.10 (+)<br>633.05 (+)<br>481.25 (+)<br>368.20 (+)	x			(Fu et al., 2014) [16]
	29.3	882	765.15 (-)<br>446.95 (-)<br>360.10 (-)<br>882.80 (+)<br>789.15 (+)<br>767.10 (+)<br>720.10 (+)<br>655.20 (+)	x			(Fu et al., 2014) [16]

			621.20 (+)<br>481.25 (+)				
2(epi)gallocatechin-3-*O*-gallate	35.5	914	913.5 (-)<br>743.2 (-)<br>423.4 (-)	x			(Fu et al., 2014) [16]
	28.6	912	911.20 (-)<br>489.20 (-)<br>455.25 (-)<br>935.15 (+)<br>913.15 (+)<br>508.05 (+)<br>333.15 (+)	x			(Fu et al., 2014) [16]
	30.3	912	911.25 (-)<br>489.25 (-)<br>455.30 (-)<br>935.15 (+)<br>913.15 (+)<br>508.25 (+)	x			(Fu et al., 2014) [16]
2-Acetyl-3,5,8-trihydroxy-1,4-naphthoquinone	19.79		247.02356 (-)	x			(Chen et al., 2017) [15]
2-Methyl-3-[2,3,5-trihydroxy-6-(hydroxymethyl)tetrahydro-2H-pyran-2-yl]-2-propenoic acid	21.54		247.08112 (-)	x			(Chen et al., 2017) [15]

3(epi)catechin-3-*O*-gallate + (epi)gallocatechin-3-*O*-gallate, 4(epi)gallocatechin-3-*O*-gallate	120.6	1827	1825.7 (-)<br>1673.4 (-)<br>1521.4 (-)<br>1369.3 (-)<br>1217.2 (-)<br>991.5 (-)	x			(Fu et al., 2014) [16]
3(epi)gallocatechin-3-*O*-gallate	25.3	1369	1367.90 (-)<br>922.55 (-)<br>911.50 (-)<br>455.25 (-)<br>1391.55 (+)<br>935.15 (+)<br>913.15 (+)<br>508.20 (+)<br>492.10 (+)	x			(Fu et al., 2014) [16]
3(epi)gallocatechin-3-*O*-gallate, 3 (epi)gallocatechin + (epi)gallocatechin-3-*O*-gallate	69.5	1369	1367.5 (-)<br>1217.4 (-)<br>1005.5 (-)<br>892.1 (-)	x			(Fu et al., 2014) [16]
	79.9	1371	1467.3 (-)<br>1369.8 (-)<br>423.4 (-)	x			(Fu et al., 2014) [16]
3-Hydroxy-β-ionone or its isomers	27.24		209.15315 (+)	x			(Chen et al., 2017) [15]
	28.15		209.15315 (+)	x			(Chen et al., 2017) [15]
4-Hydroxybenzoic acid	12.37		137.02245 (-)	x			(Chen et al., 2017) [15]

5,7-dihydroxy-6,8-dimethoxyflavone-7-*O*-glucuronide	30.4		535 (-)			x	(Spínola et al., 2014) [5]
7-oxo-N(8)-(D-ribityl)-7,8-dihydrolumazin or its isomers	32.99		313.07739 (-)	x			(Chen et al., 2017) [15]
	34.71		313.07730 (-)	x			(Chen et al., 2017) [15]
	37.24		313.07730 (-)	x			(Chen et al., 2017) [15]
	38.16		313.07733 (-)	x			(Chen et al., 2017) [15]
Acetylquercitrin	28.97		489.10226 (-)<br>491.11783 (+)	x			(Chen et al., 2017) [15]
	29.72		489.10217 (-)<br>491.11765 (+)	x			(Chen et al., 2017) [15]
Acetylstrophadogenin	32.38		461.21649 (-)	x			(Chen et al., 2017) [15]
Baicalein derivative	27.8		555 (-)			x	(Spínola et al., 2014) [5]
Benzoyl-*p*-dicoumaryl-2,7-anhydro-3-deoxy-2-octulopyranosonic acid	15.3		633 (-)			x	(Spínola et al., 2014) [5]
Benzoyl-*p*-tricoumaryl- 2,7-anhydro-3-deoxy-2-octulopyranosonic acid	31.9		779 (-)			x	(Spínola et al., 2014) [5]
Benzyl alcohol hexose pentose (formate adduct)	5.3		447 (-)			x	(Spínola et al., 2014) [5]
Butoxysuccinic acid or its isomer	19.59		189.07518 (-)	x			(Chen et al., 2017) [15]
	18.30		189.07513 (-)	x			(Chen et al., 2017) [15]
Byzantionoside A or its isomer	24.73		371.20563 (+)	x			(Chen et al., 2017) [15]

Caffeic acid derivative (formate adduct)	11.6		415 (-)			x	(Spínola et al., 2014) [5]
Caffeic acid	15.35		179.03316 (-)	x			(Chen et al., 2017) [15]
Cassioside or its isomers	24.59		415.19589 (-)	x			(Chen et al., 2017) [15]
Catechin	15.53		289.31		x<br>Relative high amount in MAE		(Kabra et al., 2019a) [14]
Chinensisol or its isomers	18.31		257.06320 (-)	x			(Chen et al., 2017) [15]
Citric acid	2.70		191.01940 (-)	x			(Chen et al., 2017) [15]
Conidendrin-*O*-hexoside	29.4		563 (-)			x	(Spínola et al., 2014) [5]
Coumaric acid	19.08		163.03812 (-)<br>165.05438 (+)	x			(Chen et al., 2017) [15]
Coumaric acid glucoside	13.92		325.09158 (-)	x			(Chen et al., 2017) [15]
Damascenone or its isomers	28.14		191.14258 (+)	x			(Chen et al., 2017) [15]
Dichotomitin-*O*-hexoside	22.0		565 (-)			x	(Spínola et al., 2014) [5]
Dihydroxybenzoic acid	7.55		153.01755 (-)	x			(Chen et al., 2017) [15]
	11.88		153.01730 (-)	x			(Chen et al., 2017) [15]
	12.75		153.01746 (-)	x			(Chen et al., 2017) [15]
Dihydroxybenzoic acid xyloside	11.41		285.06036 (-)	x			(Chen et al., 2017) [15]
Dimethoxy-dihydroxyxanthone	23.54		287.05478 (-)	x			(Chen et al., 2017) [15]

Epigallocatechin 3-O-gallate	8.55		458.37		x<br>Relative medium amount in MAE		(Kabra et al., 2019a) [14]
Epigynoside C	40.73		523.32910 (-)	x			(Chen et al., 2017) [15]
Ferulic acid-*O*-hexoside derivative	17.4		481 (-)			x	(Spínola et al., 2014) [5]
Eucommiol	25.40		187.09589 (-)	x			(Chen et al., 2017) [15]
Eudesmol acetate	11.13		266.43		x<br>Relative high amount in MAE		(Kabra et al., 2019a) [14]
Ferulic acid	20.75		193.04893 (-)	x			(Chen et al., 2017) [15]
Fulgidic acid or its isomers	32.10		327.21631 (-)	x			(Chen et al., 2017) [15]
Gallic acid	95.78		171.25		x<br>Detected in EAE		(Kabra et al., 2019a) [14]
	5.50		169.01230 (-)<br>171.0284 (+)	x			(Chen et al., 2017) [15]
Gallic acid derivative	4.3		467 (-)			x	(Spínola et al., 2014) [5]
Gallo(epi)catechin	4.8		305 (-)			x	(Spínola et al., 2014) [5]

Gallo(epi)catechin-*O*-gallate	6.8		457 (-)			x	(Spínola et al., 2014) [5]
Gallo(epi)catechin-*O*-gallate dimer	7.0		915 [2M − H]⁻			x	(Spínola et al., 2014) [5]
Galloyl(epi)gallocatechin dimer	4.7		761 (-)			x	(Spínola et al., 2014) [5]
Galloyl-bis-HHDP-*O*-hexoside (Casuarinin)	5.0		935 (-)			x	(Spínola et al., 2014) [5]
Galloylquercetin-*O*- hexoside	10.3		615 (-)			x	(Spínola et al., 2014) [5]
Geranyl rutinoside or its isomers	29.00		461.23779 (-)	x			(Chen et al., 2017) [15]
Glucosylhexanoylglucose	16.32		439.17987 (-)	x			(Chen et al., 2017) [15]
	17.30		439.18048 (-)	x			(Chen et al., 2017) [15]
	19.21		439.18030 (-)	x			(Chen et al., 2017) [15]
	20.12		439.18048 (-)	x			(Chen et al., 2017) [15]
Glucosyloctenylglucose	11.34		451.21686 (-)	x			(Chen et al., 2017) [15]
	15.37		451.21683 (-)	x			(Chen et al., 2017) [15]
Glutamylleucine(Glu-Leu)	11.72		259.12866 (-) 261.14349 (+)	x			(Chen et al., 2017) [15]
	11.03		259.12885 (-) 261.14377 (+)	x			(Chen et al., 2017) [15]
Glutamyl-valine (Glu-Val)	22.12		247.12993 (+)	x			(Chen et al., 2017) [15]
Glyceromannoheptoyl-mannose	1.74		371.11780 (-)	x			(Chen et al., 2017) [15]

Hamigeromycin B or its isomer	21.54		391.13852 (-)	x			(Chen et al., 2017) [15]
	22.22		391.13831 (-)	x			(Chen et al., 2017) [15]
Hexosylglycerol	3.40		253.09167 (-)	x			(Chen et al., 2017) [15]
Hydroxybenzoic acid	22.47		137.02240 (-)	x			(Chen et al., 2017) [15]
Hydroxythreonine	2.48		136.06157 (+)	x			(Chen et al., 2017) [15]
Kaempferol derivative	29.8		583 (-)			x	(Spínola et al., 2014) [5]
	38.5		515 (-)			x	(Spínola et al., 2014) [5]
Kaempferol rhamnoside	29.21		431.09653 (-)	x			(Chen et al., 2017) [15]
Kaempferol-*O*-(*O*-galloyl)hexoside	13.2		599 (-)			x	(Spínola et al., 2014) [5]
Kaempferol-*O*-hexoside	12.1		447 (-)			x	(Spínola et al., 2014) [5]
Kaempferol-*O*-rhamnoside	17.6		431 (-)			x	(Spínola et al., 2014) [5]
Kaempferol-*O*-rutinoside	11.0		593 (-)			x	(Spínola et al., 2014) [5]
	30.9		677 (-)			x	(Spínola et al., 2014) [5]
Kotanin or its isomers	31.09		437.12561 (-)<br>439.14136 (+)	x			(Chen et al., 2017) [15]
Lacinilene C or its isomers	26.83		247.13225 (+)	x			(Chen et al., 2017) [15]
Lactiflorin (formate adduct)	25.7		507 (-)			x	(Spínola et al., 2014) [5]

L-epicatechin	16.69		289.07062 (-)<br>291.08566 (+)	x			(Chen et al., 2017) [15]
Lupeol	41.70		424.40		x<br>Detected in AQE		(Kabra et al., 2019a) [14]
Maculalactone A	24.46		355.17169 (+)	x			(Chen et al., 2017) [15]
Manadic acid B or its isomers	31.67		325.20068 (-)	x			(Chen et al., 2017) [15]
Megastigma-diglucoside	23.35		547.23737 (-)	x			(Chen et al., 2017) [15]
Methoxyhydroxythymol	19.99		197.11684 (+)	x			(Chen et al., 2017) [15]
Methylgallic acid	12.83		183.02815 (-)	x			(Chen et al., 2017) [15]
Monascumic acid or its isomers	18.52		214.10707 (-)	x			(Chen et al., 2017) [15]
Monascumic acid or its isomers	17.52		214.10701 (-)	x			(Chen et al., 2017) [15]
Myresculoside	77.58		465.45		x<br>Detected in AQE		(Kabra et al., 2019a) [14]
Myricanol	27.24		359.42		x<br>Detected in MAE		(Kabra et al., 2019a) [14]
	32.80		359.18423 (+)	x			(Chen et al., 2017) [15]
Myricanone	17.38		355.26		x<br>Relative medium amount in MAE		(Kabra et al., 2019a) [14]

Myricetin deoxyhexoside	24.92	464	463<br>927.1	x			(Zhang et al., 2016) [12]
	31.9	464	927.20 (-)<br>531.10 (-)<br>463.05 (-)<br>951.20 (+)<br>716.50 (+)<br>487.10 (+)<br>465.10 (+)<br>319.00 (+)	x			(Fu et al., 2014) [16]
Myricetin deoxyhexoside-gallate	26.01	616	615<br>1231.2	x			(Zhang et al., 2016) [12]
	11.6	616	615.4 (-)<br>317.3 (-)	x			(Fu et al., 2014) [16]
	37.6	616	1231.20 (-)<br>615.10 (-)<br>944.55 (+)<br>639.15 (+)<br>617.15 (+)	x			(Fu et al., 2014) [16]
	37.7	616	615.1 (-)<br>639.50 (+)<br>588.40 (+)<br>566.50 (+)	x			(Fu et al., 2014) [16]
Myricetin hexoside	22.54	480	479<br>959.1	x			(Zhang et al., 2016) [12]

Myricetin-3-*O*-(2″-*O*-galloyl)-β-D-galactopyranoside	22.52	632	631<br>1263.1	x			(Zhang et al., 2016) [12]
Myricetin-*O*-(*O*-galloyl)deoxyhexoside	16.3		615 (-)			x	(Spínola et al., 2014) [5]
	17.3		615 (-)			x	(Spínola et al., 2014) [5]
Myricetin-*O*-(*O*-galloyl)hexoside	7.7		631 (-)			x	(Spínola et al., 2014) [5]
Myricetin-*O*-deoxyhexoside	9.7		463 (-)			x	(Spínola et al., 2014) [5]
Myricetin-*O*-hexoside	7.5		479 (-)			x	(Spínola et al., 2014) [5]
Myricetinylmyricitrin	19.03		779.10736 (-)	x			(Chen et al., 2017) [15]
	20.52		779.10767 (-)	x			(Chen et al., 2017) [15]
Myricitrin	24.25	464	463<br>927.1	x			(Zhang et al., 2016) [12]
	23.66		463.08621 (-)	x			(Chen et al., 2017) [15]
Myricitrin gallate	26.34		615.09674 (-)<br>617.1131 (+)	x			(Chen et al., 2017) [15]
	27.05		615.09650 (-)<br>617.11261 (+)	x			(Chen et al., 2017) [15]
Myricitrinyl-(epi)gallo-catechin	13.30		767.14398 (-)	x			(Chen et al., 2017) [15]
	16.77		767.14398 (-)	x			(Chen et al., 2017) [15]
	19.17		767.14374 (-)	x			(Chen et al., 2017) [15]

	20.38		767.14392 (-)	x			(Chen et al., 2017) [15]
Myricitrinyl-myricitrin	16.07		925.16467 (-)<br>927.18079 (+)	x			(Chen et al., 2017) [15]
Myricitrinylmyritrin gallate	20.96		1077.17590 (-)	x			(Chen et al., 2017) [15]
	24.06		1077.17590 (-)	x			(Chen et al., 2017) [15]
	19.22		1077.17542 (-)	x			(Chen et al., 2017) [15]
Myricitrinyl-quercetin-arabinoside	19.98		897.17059 (-)	x			(Chen et al., 2017) [15]
	20.52		897.17053 (-)	x			(Chen et al., 2017) [15]
N-(1-Deoxyfructopyranos-1-yl) isoleucylaspartic acid or its isomers	19.77		409.18253 (+)	x			(Chen et al., 2017) [15]
	17.05		409.18228 (+)	x			(Chen et al., 2017) [15]
	16.42		409.1821 (+)	x			(Chen et al., 2017) [15]
n-hexadecanol	12.94		241.40		x<br>Relative medium amount in MAE		(Kabra et al., 2019a) [14]
n-octadecanol	11.13		269.08<br>269.34		x<br>Relative high amount in MAE		(Kabra et al., 2019a) [14]
n-pentadecanol	11.13		227.31<br>227.98		x<br>Relative high		(Kabra et al., 2019a) [14]

					amount in MAE		
O-Acetylshanzhiside methyl ester or its isomers	14.84		447.14896 (-)	x			(Chen et al., 2017) [15]
O-Acetylshanzhiside methyl ester or its isomers	15.80		447.14896 (-)	x			(Chen et al., 2017) [15]
Oleanolic acid	8.85		456.71		x Relative medium amount in MAE		(Kabra et al., 2019a) [14]
Oleuropein	14.1		539 (-)			x	(Spínola et al., 2014) [5]
Palmitic acid	12.15		257.21		x Relative medium amount in MAE		(Kabra et al., 2019a) [14]
p-coumaric acid	15.53		163.22 163.78		x Relative high amount in MAE		(Kabra et al., 2019a) [14]
Pectolinaringenin glucuronoside	13.02		489.10223 (-)	x			(Chen et al., 2017) [15]
Pentylurofuransaeure	29.49		267.12286 (-)	x			(Chen et al., 2017) [15]
Phenylalanine	6.62		166.08583 (+)	x			(Chen et al., 2017) [15]

Phylligenin-*O*-hexoside (formate adduct)	12.5		579 (-)			x	(Spínola et al., 2014) [5]
Plucheoside C or its isomers	13.57		443.19055 (-)	x			(Chen et al., 2017) [15]
Propylshanzhiside methyl ester or its isomers	19.65		431.19061 (-)	x			(Chen et al., 2017) [15]
	16.19		431.19016 (-)	x			(Chen et al., 2017) [15]
	16.88		431.19049 (-)	x			(Chen et al., 2017) [15]
Protocatechuic acid	8.92		153.01740 (-)	x			(Chen et al., 2017) [15]
Protocathechuic acid-*O*-pentoside	5.9		285 (-)			x	(Spínola et al., 2014) [5]
Punarnavoside	28.12		527.18799 (+)	x			(Chen et al., 2017) [15]
Quercetin	25.81		299.03		x Relative medium amount in MAE		(Kabra et al., 2019a) [14]
Quercetin 3-rhamnoside	26.52	448	447 895.1	x			(Zhang et al., 2016) [12]
Quercetin-*O*-(*O*-galloyl)deoxyhexoside	23.8		599 (-)			x	(Spínola et al., 2014) [5]
Quercetin-*O*-acetylrhamnoside	21.7		489 (-)			x	(Spínola et al., 2014) [5]
Quercetin-*O*-deoxyhexoside	13.4		447 (-)			x	(Spínola et al., 2014) [5]
Quercitrin	26.88		447.09131 (-) 449.10699 (+)	x			(Chen et al., 2017) [15]

Quercitrinyl-(epi)gallocatechin methyl gallate	26.81		917.17169 (-)<br>919.18896 (+)	x			(Chen et al., 2017) [15]
Quercitrinylquercetingalactoside	22.06		895.15479 (-)	x			(Chen et al., 2017) [15]
Quinic acid	3.3		191 (-)			x	(Spínola et al., 2014) [5]
	1.87		191.05421 (-)	x			(Chen et al., 2017) [15]
Quinic acid derivative	3.1		533 (-)			x	(Spínola et al., 2014) [5]
Quinic acid dimer	3.8		383 $[2M-H]^-$			x	(Spínola et al., 2014) [5]
Secoisolariciresinol glucoside	23.69		525.22955 (+)	x			(Chen et al., 2017) [15]
	24.23		525.22931 (+)	x			(Chen et al., 2017) [15]
Sinapic acid-*O*-hexoside derivative	5.6		421 (-)			x	(Spínola et al., 2014) [5]
	16.5		511 (-)			x	(Spínola et al., 2014) [5]
Stigmasterol	12.15		415.52		x<br>Relative high amount in MAE		(Kabra et al., 2019a) [14]
Stophanthobiose or its isomers	13.83		293.12311 (-)	x			(Chen et al., 2017) [15]
	14.35		293.12323 (-)	x			(Chen et al., 2017) [15]
Tricin-*O*-hexoside	26.6		491 (-)			x	(Spínola et al., 2014) [5]

	27.3		491 (-)			x	(Spínola et al., 2014) [5]
	29.1		491 (-)			x	(Spínola et al., 2014) [5]
Trimethoxybenzoic acid methyl ester	28.04		225.07535 (-)	x			(Chen et al., 2017) [15]
Tryptophan	10.88		205.09671 (+)	x			(Chen et al., 2017) [15]
Tyrosine	4.03		182.08076 (+)	x			(Chen et al., 2017) [15]
Undecanetricarboxylic acid	26.16		287.14893 (-)	x			(Chen et al., 2017) [15]
Unidentified	2.04		407.11517 (+)	x			(Chen et al., 2017) [15]
Unidentified	2.66		247.05724 (+)	x			(Chen et al., 2017) [15]
Unidentified	3.00		405.02664 (-) 407.04236 (+)	x			(Chen et al., 2017) [15]
Unidentified	3.43		247.05724 (+)	x			(Chen et al., 2017) [15]
Unidentified	5.65		779.14288 (-)	x			(Chen et al., 2017) [15]
Unidentified	5.75		215.05214 (+)	x			(Chen et al., 2017) [15]
Unidentified	6.02		267.08325 (+)	x			(Chen et al., 2017) [15]
Unidentified	6.85		287.10944 (+)	x			(Chen et al., 2017) [15]
Unidentified	7.40		319.04367 (+)	x			(Chen et al., 2017) [15]
Unidentified	9.32		355.09918 (+)	x			(Chen et al., 2017) [15]
Unidentified	9.77		267.15613 (+)	x			(Chen et al., 2017) [15]
Unidentified	10.10		259.11459 (+)	x			(Chen et al., 2017) [15]
Unidentified	10.15		405.04822 (-)	x			(Chen et al., 2017) [15]

Unidentified	10.52		259.11469 (+)	x			(Chen et al., 2017) [15]
Unidentified	10.79		407.09415 (+)	x			(Chen et al., 2017) [15]
Unidentified	11.46		429.20868 (+)	x			(Chen et al., 2017) [15]
Unidentified	11.67		259.11459 (+)	x			(Chen et al., 2017) [15]
Unidentified	12.05		405.04803 (-)	x			(Chen et al., 2017) [15]
Unidentified	12.44		209.15317 (+)	x			(Chen et al., 2017) [15]
Unidentified	12.44		267.15585 (+)	x			(Chen et al., 2017) [15]
Unidentified	12.69		413.16501 (-)	x			(Chen et al., 2017) [15]
Unidentified	12.79		391.15674 (+)	x			(Chen et al., 2017) [15]
Unidentified	13.42		229.10657 (+)	x			(Chen et al., 2017) [15]
Unidentified	13.55		284.02228 (-)	x			(Chen et al., 2017) [15]
Unidentified	13.69		429.20895 (+)	x			(Chen et al., 2017) [15]
Unidentified	13.78		467.18823 (+)	x			(Chen et al., 2017) [15]
Unidentified	14.97		399.00098 (-)	x			(Chen et al., 2017) [15]
Unidentified	15.13		361.14612 (+)	x			(Chen et al., 2017) [15]
Unidentified	15.13		425.14111 (+)	x			(Chen et al., 2017) [15]
Unidentified	16.05		425.1409 (+)	x			(Chen et al., 2017) [15]
Unidentified	16.65		261.00641 (-)	x			(Chen et al., 2017) [15]
Unidentified	16.95		399.00122 (-)	x			(Chen et al., 2017) [15]
Unidentified	18.07		269.13837 (-)	x			(Chen et al., 2017) [15]
Unidentified	18.19		449.28891 (+)	x			(Chen et al., 2017) [15]
Unidentified	18.54		217.10669 (-)	x			(Chen et al., 2017) [15]

Unidentified	19.19		138.01768 (-)	x			(Chen et al., 2017) [15]
Unidentified	19.33		307.11456 (+)	x			(Chen et al., 2017) [15]
Unidentified	23.66		387.06375 (-)	x			(Chen et al., 2017) [15]
Unidentified	24.25		445.14612 (+)	x			(Chen et al., 2017) [15]
Unidentified	24.97		527.24567 (+)	x			(Chen et al., 2017) [15]
Unidentified	25.26		300.99777 (-)	x			(Chen et al., 2017) [15]
Unidentified	26.38		311.14572 (+)	x			(Chen et al., 2017) [15]
Unidentified	26.84		287.12463 (+)	x			(Chen et al., 2017) [15]
Unidentified	29.72		507.20621 (-)	x			(Chen et al., 2017) [15]
Unidentified	30.15		485.19852 (+)	x			(Chen et al., 2017) [15]
Unidentified	30.95		407.11530 (-)	x			(Chen et al., 2017) [15]
Unidentified	31.09		897.24121 (-)	x			(Chen et al., 2017) [15]
Unidentified	32.34		237.0786 (+)	x			(Chen et al., 2017) [15]
Unidentified	32.45		297.04584 (-)	x			(Chen et al., 2017) [15]
Unidentified	32.55		239.05843 (-)	x			(Chen et al., 2017) [15]
Unidentified	32.55		285.06400 (-)	x			(Chen et al., 2017) [15]
Unidentified	32.71		359.08258 (-)	x			(Chen et al., 2017) [15]
Unidentified	32.93		274.27322 (+)	x			(Chen et al., 2017) [15]
Unidentified	32.98		461.21652 (-)	x			(Chen et al., 2017) [15]
Unidentified	35.71		433.10092 (-)	x			(Chen et al., 2017) [15]
Unidentified	35.84		411.09274 (+)	x			(Chen et al., 2017) [15]
Unidentified	35.91		165.03900 (-)	x			(Chen et al., 2017) [15]

Unidentified	36.17		279.15833 (+)	x			(Chen et al., 2017) [15]
Unidentified	36.79		371.10034 (+)	x			(Chen et al., 2017) [15]
Unidentified	36.81		507.11948 (-)	x			(Chen et al., 2017) [15]
Unidentified	36.81		223.02686 (-)	x			(Chen et al., 2017) [15]
Unidentified	36.81		165.03899 (-)	x			(Chen et al., 2017) [15]
Unidentified	36.95		297.04584 (-)	x			(Chen et al., 2017) [15]
Unidentified	37.31		581.13831 (-)	x			(Chen et al., 2017) [15]
Unidentified	38.22		655.15680 (-)	x			(Chen et al., 2017) [15]
Unidentified	38.76		729.17535 (-)	x			(Chen et al., 2017) [15]
Unidentified	38.81		707.16736 (+)	x			(Chen et al., 2017) [15]
Unidentified	39.18		803.19385 (-)	x			(Chen et al., 2017) [15]
Unidentified	39.50		877.21265 (-)	x			(Chen et al., 2017) [15]
Unidentified	39.50		951.23090 (-)	x			(Chen et al., 2017) [15]
Unknown	3.0		683 $[2M-H]^-$			x	(Spínola et al., 2014) [5]
Unknown	18.5		549 (-)			x	(Spínola et al., 2014) [5]
Unknown	32.8		695 (-)			x	(Spínola et al., 2014) [5]
Unknown	33.6		673 (-)			x	(Spínola et al., 2014) [5]
Unknown	34.8		515 (-)			x	(Spínola et al., 2014) [5]
Urauchimycin C	24.79		409.16147 (+)	x			(Chen et al., 2017) [15]

Veratroyl ethylene glycol or its iso mers	6.02		227.09087 (+)	x			(Chen et al., 2017) [15]
β-caryophyllene	41.68		205.10		x<br>Detected in AQE		(Kabra et al., 2019a) [14]
β-sitosterol	12.15		415.52<br>416.45		x<br>Relative high amount in MAE		(Kabra et al., 2019a) [14]

MAE = Methanolic extract | EAE = Ethyl acetate extract | AQE = Aqueous extract

References

1. Zhang, X.; Huang, H.; Zhang, Q.; Fan, F.; Xu, C.; Sun, C.; Li, X.; Chen, K. Phytochemical Characterization of Chinese Bayberry (*Myrica rubra* Sieb. et Zucc.) of 17 Cultivars and Their Antioxidant Properties. *International Journal of Molecular Sciences* **2015**, *16*, 12467-12481, doi:10.3390/ijms160612467.
2. Chen, F.; Wang, Y.; Ni, H.; Yan, B. The 3at gene determines fruit color of *Myrica rubra* (*Myricaceae*). *Genetics and Molecular Research* **2020**, *19*, 1-18, doi:10.4238/gmr18371.
3. Ngurthankhumi, R.; Hazarika, T.K.; Lalruatsangi, E.; Lalnunsangi, T.; Lalhmangaihzuali, H.P. Bioactive constituents and health promoting compounds of few wild edible fruits of North-East India. *International Journal of Food Properties* **2024**, *27*, 927-950, doi:10.1080/10942912.2024.2374498.
4. Rymbai, H.; Verma, V.K.; Talang, H.; Assumi, S.R.; Devi, M.B.; Sangma, R.H.C.H.; Biam, K.P.; Chanu, L.J.; Makdoh, B.; Singh, A.R.; et al. Biochemical and antioxidant activity of wild edible fruits of the eastern Himalaya, India. *Frontiers in Nutrition* **2023**, *10*, doi:10.3389/fnut.2023.1039965.
5. Spínola, V.; Llorent-Martínez, E.J.; Gouveia, S.; Castilho, P.C. *Myrica faya*: A new source of antioxidant phytochemicals. *Journal of Agricultural and Food Chemistry* **2014**, *62*, 9722-9735, doi:10.1021/jf503540s.
6. Llorent-Martinez, E.J.; Spinola, V.; Castilho, P.C. Evaluation of the inorganic content of six underused wild berries from Portugal: Potential new sources of essential minerals. *Journal of Food Composition and Analysis* **2017**, *59*, 153-160, doi:10.1016/j.jfca.2017.02.016.
7. Xia, W.; Gong, E.R.; Lin, Y.Y.; Zheng, B.S.; Yang, W.H.; Li, T.; Zhang, S.; Li, P.; Liu, R.H. Wild pink bayberry free phenolic extract induces mitochondria-dependent apoptosis and G0/G1 cell cycle arrest through p38/MAPK and PI3K/Akt pathway in MDA-MB-231 cancer cells. *Food Science and Human Wellness* **2023**, *12*, 1510-1518, doi:10.1016/j.fshw.2023.02.014.
8. Ren, H.; Yu, H.; Zhang, S.; Liang, S.; Zheng, X.; Zhang, S.; Yao, P.; Zheng, H.; Qi, X. Genome sequencing provides insights into the evolution and antioxidant activity of Chinese bayberry. *BMC Genomics* **2019**, *20*, 458, doi:10.1186/s12864-019-5818-7.
9. Spínola, V.; Llorent-Martínez, E.J.; Castilho, P.C. Polyphenols of *Myrica faya* inhibit key enzymes linked to type II diabetes and obesity and formation of advanced glycation end-products (i*n vitro*): Potential role in the prevention of diabetic complications. *Food Research International* **2019**, *116*, 1229-1238, doi:10.1016/j.foodres.2018.10.010.
10. Yun, D.W.; Cai, H.H.; Liu, Y.P.; Xiao, L.X.; Song, J.F.; Liu, J. Development of active and intelligent films based on cassava starch and Chinese bayberry (*Myrica rubra* Sieb. et Zucc.) anthocyanins. *RSC Advances* **2019**, *9*, 30905-30916, doi:10.1039/c9ra06628d.
11. Kabra, A.; Sharma, R.; Singla, S.; Kabra, R.; Baghel, U.S. Pharmacognostic characterization of *Myrica esculenta* leaves. *Journal of Ayurveda and Integrative Medicine* **2019**, *10*, 18-24, doi:10.1016/j.jaim.2017.07.012.
12. Zhang, Y.; Chen, S.; Wei, C.; Gong, H.; Li, L.; Ye, X. Chemical and Cellular Assays Combined with In Vitro Digestion to Determine the Antioxidant Activity of Flavonoids from Chinese Bayberry (*Myrica rubra* Sieb. et Zucc.) Leaves. *PLoS One* **2016**, *11*, e0167484, doi:10.1371/journal.pone.0167484.
13. Zhang, Y.; Chen, S.; Wei, C.; Rankin, G.O.; Rojanasakul, Y.; Ren, N.; Ye, X.; Chen, Y.C. Dietary Compound Proanthocyanidins from Chinese bayberry (*Myrica rubra* Sieb. et Zucc.) leaves inhibit angiogenesis and regulate cell cycle of cisplatin-resistant ovarian cancer cells via targeting Akt pathway. *Journal of functional foods* **2018**, *40*, 573-581, doi:10.1016/j.jff.2017.11.045.
14. Kabra, A.; Sharma, R.; Hano, C.; Kabra, R.; Martins, N.; Baghel, U.S. Phytochemical Composition, Antioxidant, and Antimicrobial Attributes of Different Solvent Extracts

from *Myrica esculenta* Buch.-Ham. ex. D. Don Leaves. *Biomolecules* **2019**, *9*, doi:10.3390/biom9080357.

15. Chen, P.; Lin, X.; Yang, C.H.; Tang, X.; Chang, Y.W.; Zheng, W.; Luo, L.; Xu, C.; Chen, Y.H. Study on Chemical Profile and Neuroprotective Activity of *Myrica rubra* Leaf Extract. *Molecules* **2017**, *22*, doi:10.3390/molecules22071226.
16. Fu, Y.; Qiao, L.; Cao, Y.; Zhou, X.; Liu, Y.; Ye, X. Structural elucidation and antioxidant activities of proanthocyanidins from Chinese bayberry (*Myrica rubra* Sieb. et Zucc.) leaves. *PLoS One* **2014**, *9*, e96162, doi:10.1371/journal.pone.0096162.
